# Schistosomiasis mansoni and alcohol abuse comorbidity: Prevalence and risk factors among adults in Makenene, Cameroon

**DOI:** 10.1371/journal.pntd.0013687

**Published:** 2026-07-06

**Authors:** Emmanuelle S. Yimgoua, Christian M. Kenfack, Nestor G. Feussom, Joseph B. Fassi-Kadji, Emilienne T. Nkondo, Ulrich M. Femoe, Louis-Albert Tchuem Tchuente, Hermine B. Jatsa

**Affiliations:** 1 Department of Animal Biology and Physiology, Faculty of Science, Laboratory of Animal Physiology and Therapeutic Research, University of Yaoundé I, Yaoundé, Cameroon; 2 Centre for Schistosomiasis and Parasitology, Yaoundé, Cameroon; 3 Laboratory of Parasitology and Ecology, Department of Animal Biology and Physiology, Faculty of Science, University of Yaoundé I, Yaoundé, Cameroon; Translational Research and Development Foundation (TREND Foundation), CAMEROON

## Abstract

**Background:**

Alcohol consumption in Cameroon is approximately twice the African average, and Makenene is a hotspot of *Schistosoma mansoni* infection in the country. Considering that both schistosomiasis mansoni and alcohol abuse can induce liver pathology, this study aimed to investigate the prevalence and risk factors of *S. mansoni* and alcohol abuse comorbidity in Makenene, Cameroon.

**Methodology/Principal findings:**

A cross-sectional survey was conducted in Makenene, including 431 adults from four communities. *Schistosoma mansoni* was diagnosed using Kato-Katz (KK) and point-of-care circulating cathodic antigen (POC-CCA) tests. Participants were considered *S. mansoni*-positive if either test was positive. The Alcohol Use Disorders Identification Test (AUDIT) was used to determine the alcohol dependence score (ADS). Sociodemographic, behavioral, and environmental factors were also recorded. The prevalence of *S. mansoni* was 19.6% (95% CI: 15.7–24.0) by KK and 66.1% (95% CI: 58.8–73.3) by POC-CCA, yielding a prevalence of 34.6% (95% CI: 30.2 – 39.0) in the study population. The prevalence of alcohol abuse was 54.3% (95% CI: 49.4–58.7). *S. mansoni* infection and alcohol abuse comorbidity prevalence was 17.4% (95% CI: 13.9 – 21.1). Independent predictors of this comorbidity included the community of residence, with the risk being nearly fivefold higher in Baloua (AOR = 4.99; 95% CI: 2.36–10.56; p < 0.001) and over twofold higher in Carrière (AOR = 2.37; 95% CI: 1.19–4.70; p = 0.013). Furthermore, regular consumption of palm wine (AOR = 2.72; 95% CI: 1.39–5.34; p = 0.003) and beer (AOR = 2.00; 95% CI: 1.06–3.80; p = 0.032), as well as, being aged 18–39 years (AOR = 1.88; 95% CI: 1.02–3.46; p = 0.042), were also identified as significant independent factors.

**Conclusion/significance:**

This study confirms *S. mansoni* endemicity in Makenene and reveals a high prevalence of alcohol misuse, highlighting the comorbidity. These findings call for integrated interventions targeting both schistosomiasis transmission and harmful alcohol use.

## Author summary

This study presents, for the first time, documented data on the comorbidity between *Schistosoma mansoni* infection and alcohol abuse in a rural area of Cameroon. Through parasitological, sociodemographic, and behavioral approaches, the study revealed the co-occurrence of schistosomiasis and alcohol abuse comorbidity in Makenene. In the adult population, the prevalence of *S. mansoni* infection was 34.6%, alcohol abuse was 54.3%, and 17.4% of participants exhibited the comorbidity of both conditions. The risk factors for the comorbidity included younger age (18–39 years), living in the nearest riverine communities of Baloua and Carrière, and frequent consumption of palm wine and beer. The concomitance of intestinal schistosomiasis and alcohol abuse, two major health problems, highlights the urgency of investigating the reciprocal influence of each condition on the clinical severity of the other. Developing targeted interventions, such as integrating alcohol abuse screening and awareness of moderate alcohol consumption into mass drug administration campaigns, could help simultaneously reduce the burden of schistosomiasis and alcohol abuse in semi-rural and rural areas.

## Introduction

Schistosomiasis is one of the most prevalent neglected tropical diseases, caused by parasitic trematodes of the genus *Schistosoma* [1]. Populations are exposed through agricultural, domestic, occupational, or recreational activities that involve contact with freshwater contaminated by cercariae. Cercariae are free-swimming larvae released by freshwater snails, which serve as intermediate hosts after becoming infected by water contaminated with human excreta containing parasite eggs [1,2]. In 2022, preventive chemotherapy targeting school-age children and high-risk adults was required for schistosomiasis in 50 countries for a total of 264.3 million people, including Cameroon [3]. Endemic areas are concentrated in tropical and subtropical regions, particularly among impoverished communities lacking access to safe water and adequate sanitation [1,3,4]. Globally, more than 700 million people are at risk of infection, and approximately 230 million are currently infected. The disease burden is especially high in sub-Saharan Africa, which accounts for 85% of cases and the majority of severe forms, with an estimated 200,000 deaths annually [5].

In Cameroon, the National Programme for the Control of Schistosomiasis and Intestinal Helminthiasis (PNLSHI) estimated that over 2 million people were infected in 2020 [6]. Schistosomiasis occurs in all ten regions of the country, with three human-infecting species identified: *Schistosoma haematobium, Schistosoma mansoni,* and *Schistosoma guineensis*. The Centre region reports the highest prevalence of *S. mansoni* infection (9.49%), with certain hotspots showing much higher rates, such as Makenene, where prevalence was 41% in 2010 [7] and 40,7% in 2019 [8]. Control strategies of schistosomiasis rely primarily on large-scale praziquantel administration to school-age children, supported by health education, vector control, improved sanitation, safe water supply, and environmental management of transmission sites, all of which are critical to achieve sustainable interruption of transmission [1–4].

*Schistosoma mansoni* infection is exacerbated by associated comorbidities. Among these, alcohol abuse is of particular concern due to its potential synergistic impact on hepatic pathology, since the liver is the main target organ in both conditions [9–11]. Classified among the most widely used psychoactive substances worldwide, alcohol is consumed by 43% of the global population, with alcohol use disorders affecting more than 600 million people [12,13]. In Sub-Saharan Africa, alcohol consumption is deeply rooted in social and cultural practices, characterized by the widespread availability of both industrial and artisanal beverages. Several studies conducted in rural areas have reported significant levels of alcohol consumption among adults, particularly in males and within economically active populations [14,15]. In 2019, alcohol consumption accounted for 2.6 million deaths, representing 4.7% of global mortality [13]. Cameroon ranked fourth among 49 African countries assessed in 2019 for per capita alcohol consumption. The average of 10.1 liters of pure alcohol per person aged 15 years and older was more than double the African average of 4.5 L/person/year [13]. Given that alcohol is a widely consumed substance in Cameroon, we hypothesize that there is a significant prevalence of co-occurrence between *Schistosoma mansoni* infection and harmful alcohol use within the studied population, and that this comorbidity is closely associated with specific sociodemographic factors and local risk behaviors.

Alcohol misuse is the leading cause of liver disease, driving progressive damage including steatosis, alcoholic hepatitis, fibrosis, cirrhosis, and hepatocellular carcinoma [10,11]. Like chronic alcoholism, *S. mansoni* infection severely compromises liver function. At the chronic stage, granulomatous inflammation induced by parasite eggs leads to periportal fibrosis, often associated with splenomegaly and portal hypertension [9,16]. Concomitant alcohol intoxication may exacerbate schistosomiasis-related hepatic damage, amplifying clinical and pathophysiological consequences at the systemic level. Recent studies based on animal models further confirm that the association between alcohol consumption and *Schistosoma mansoni* infection may exert synergistic deleterious effects on target organs. In particular, this comorbidity has been associated with an exacerbation of hepatic lesions [17], while ethanol exposure has also been reported as an aggravating factor for schistosomiasis-induced renal damage [18].

However, despite this pathophysiological convergence, epidemiological data on the coexistence of schistosomiasis and alcohol abuse in humans remain extremely scarce. Most studies conducted in schistosomiasis-endemic areas examine these two conditions separately, without exploring their overlap within the same populations or the associated risk factors. Therefore, it appears necessary to provide new scientific insight into the interaction between these two major public health issues. An approach that integrates alcohol consumption habits within endemic populations and identifies the underlying risk behaviors contributing to the persistence of schistosomiasis transmission would thus support schistosomiasis elimination efforts.

Based on this rationale, the present study aimed to determine the prevalence of schistosomiasis and alcohol abuse comorbidity among adults in an intestinal schistosomiasis-endemic area of Makenene, Cameroon, as well as the risk factors associated with this comorbidity.

## Results

### Socio-environmental characteristics of the study population

A total of 431 participants were included in the study, consisting of 199 women (46.2%) and 232 men (53.8%), yielding a sex ratio of 1.17. The population was predominantly composed of farmers and livestock breeders (61.7%), with the majority residing within 500 m of the main schistosomiasis transmission site (70.5%). Regarding educational attainment, most participants had reached, but not necessarily completed, secondary-level education, reflecting a generally low-to-moderate level of schooling (54.3%). The age distribution showed that nearly half of the participants (45.5%) were young adults aged 18–39 years. The overall median age of the study population was 42.0 years (IQR: 30.0 – 54.0), ranging from 18 to 90 years (Table 1).

**Table 1:** Socio-environmental description of the population.

Variables	Modalities	Sample size (N = 431)	Frequency (%)
**Community**	Baloua	115	26.7
	Carrière	131	30.4
	Mock Centre	103	23.9
	Mock Sud	82	19.0
**Sex**	Male	232	53.8
	Female	199	46.2
**Age groups (years)**	18 – 39	196	45.5
	40 – 59	162	37.6
	≥ 60	73	16.9
**Level of education**	None	35	8.1
	Primary	146	33.9
	Secondary	234	54.3
	Higher	16	3.7
**Main occupation**	Farmer/Breeder	266	61.7
	Housewife	61	14.2
	Technician	33	7.7
	Student	22	5.1
	Salesperson	21	4.9
	Unemployed/Retired	10	2.3
	Other	18	4.2
**Distance from transmission site (meters)**	< 500	304	70.5
	500 – 1000	76	17.6
	> 1000	51	11.8

“Other” indicated the following occupations: medical staff, teaching staff, motorcycle taxi drivers, management staff, clergy, and veterinarians.

### Prevalence and severity of *Schistosoma mansoni* infection

#### Prevalence and severity of infection in the overall study population.

Prevalence estimates varied according to the diagnostic method and the sample analyzed. Among participants who provided urine samples, the POC-CCA detected 109 positive cases out of 165 examined, corresponding to a prevalence of 66.1% (95% CI: 58.8–73.3). In contrast, the KK method, applied to a larger group of stool samples, identified 75 positive cases among 383 participants, yielding a prevalence of 19.6% (95% CI: 15.7–24.0). Among participants, 35 were positive for both diagnostic methods.

To generate an overall estimate for the entire cohort (N = 431), a participant was considered infected if at least one of the two diagnostic methods was positive. Based on this criterion, 149 participants were classified as infected with *Schistosoma mansoni*, resulting in an overall prevalence of 34.6% (95% CI: 29.7–39.0).

The severity of infection, assessed based on the mean egg load among participants diagnosed positive by the KK method (n = 75), was 100.3 EPG (95% CI: 78.5 – 128.3), corresponding to a moderate infection intensity in the overall infected population; however, values ranged from 24 to 4 440 EPG. According to the WHO stratification, infection was classified as light in 57.3% of cases, moderate in 33.3%, and heavy in 9.3% (Table 2).

**Table 2:** Infection intensity of *Schistosoma mansoni*-positive participants.

	Low intensity	Moderate intensity	Heavy intensity	Overall intensity
**n**_**kk**_ **(%)**	43 (57.3%)	25 (33.3%)	7 (9.3)	75
**Mean EPG**	48.3	189.6	918.5	100.3
**95% CI**	41.5 – 56.4	163.5 – 219.8	446.1 – 1891.2	78.5 – 128.3
***p***	< 0.001			

nₖₖ: Schistosomiasis cases diagnosed by Kato-Katz; EPG: Egg per gram of stool; Mean EPG is the geometric mean; 95% CI: 95% confidence interval associated with the mean of infection intensity; Low intensity = < 100 EPG; Moderate intensity = 100–400 EPG; Heavy intensity = > 400 EPG.

#### Prevalence and severity of *Schistosoma mansoni* infection according to socio-demographic characteristics.

At the community level, Baloua had the highest prevalence of 50.4% (95% CI: 41.0–59.3), followed by Carrière with 33.6% (95% CI: 25.7–42.4). Lower prevalence were observed in Mock Sud (28.0%; 95% CI: 18.7–38.7) and Mock Centre (23.3%; 95% CI: 15.5–31.3). A significant difference was observed between those two communities and Baloua (*p* < 0.001). The parasite burden, which reflects infection severity, was 88.4 EPG (95% CI: 52.0 – 150.2) and 90.9 EPG (95% CI: 57.3 – 144.2) in Carrière and Mock Centre, respectively, reflecting a light infection intensity. A moderate infection intensity was recorded in Baloua (109.4 EPG (95% CI: 73.0 – 163.7) and Mock Sud (146.4 EPG (95% CI: 43.3 – 495.6). However, no significant difference was observed at the community level (*p* = 0.608) (Table 3).

**Table 3:** Distribution and severity of *Schistosoma mansoni* infection according to sociodemographic criteria.

Variable	Prevalence of *S. mansoni*				Intensity of infection			
	N = 431	n (%)	95% CI	*p*	n_kk_ = 75	Mean EPG	95% CI	*p*
**Community**								
Baloua	115	58 (50.4) ^**ms, mc**^	41.0 - 59.3	< 0.001	33	109.4	73.0 – 163.7	0.608
Carrière	131	44 (33.6)	25.7 - 42.4		21	88.4	52.0 – 150.2	
Mock Centre	103	24 (23.3) ^**b**^	15.5 - 31.3		17	90.9	57.3 – 144.2	
Mock Sud	82	23 (28.0) ^**b**^	18.7 - 38.7		4	146.4	43.3 – 495.6	
**Sex**								
Male	232	72 (31.0)	25.0 – 37.1	0.096	36	71.7	50.9 – 101.1	0.003
Female	199	77 (38.7)	31.7 – 46.2		39	136.8	97.8 – 191.3	
**Age groups (years)**								
18 – 39	196	87 (44.4) ^**β,γ**^	37.8 – 51.4	< 0.001	36	113.8	75.5 – 171.4	0.673
40 – 59	162	46 (28.4) ^**α**^	21.0 – 35.4		28	92.1	63.9 – 132.6	
≥ 60	73	16 (21.9) ^**α**^	12.9 – 31.4		11	82.8	45.0 – 152.3	

N: Total sample size; n: Schistosomiasis cases; nₖₖ: Schistosomiasis cases diagnosed by Kato-Katz; EPG: Egg per gram of stool; Mean EPG is the geometric mean; 95% CI: 95% confidence interval associated with the prevalence or the mean of infection intensity.

b: significantly different from Baloua; c: significantly different from Carrière; mc: significantly different from Mock Centre; ms: significantly different from Mock Sud. α: significantly different from 18–39 years; β: significantly different from 40–59 years; γ: significantly different from ≥ 60 years.

By sex, prevalence tended to be higher among women (38.7%) than men (31.0%), although this difference did not reach statistical significance (*p* = 0.096). Likewise, a significant difference in parasite burden was recorded between sexes (*p* = 0.003). In men, the parasite burden fell into the light infection intensity category (71.7 EPG (95% CI: 50.9 – 101.1)), while for women, it corresponded to moderate infection intensity (136.8 EPG (95% CI: 97.8 – 191.3)) (Table 3).

Across age groups, prevalence decreased significantly with increasing age (*p* < 0.001). Participants aged 18–39 years had the highest prevalence of 44.4% (95% CI: 37.8–51.4), compared to 28.4% (95% CI: 21.0–35.4) for the 40–59 years and 21.9% (95% CI: 12.9–31.4) for participants aged ≥ 60 years. Similarly, mean egg counts decreased with age, from 113.8 EPG (95% CI: 75.5 – 171.4) for the 18–39 years participants to 82.8 EPG (95% CI: 45.0 – 152.3) among those aged ≥ 60 years (*p* = 0.673). Thus, only the 18–39-year-old age group had moderate infection intensity, with the other age groups falling into the light intensity category (Table 3).

### Prevalence of alcohol consumption levels

#### Prevalence of alcohol consumption levels in the general population.

The distribution of participants according to their level of alcohol consumption (Table 4) revealed a markedly unequal pattern within the study population (*p* < 0.001). The majority of the participants, 42.9% (95% CI: 38.3–47.3), were classified in the “low-risk use” category (AUDIT = 1–6). The “harmful use” category (AUDIT = 7–12) was represented by 28.8% (95% CI: 24.6–32.9) of the participants, while 25.5% (95% CI: 21.6–29.7) of the participants fell in the “alcohol dependence” category (AUDIT ≥ 13). In contrast, the non-consumers of alcohol, the “non-use” category (AUDIT = 0), represented 2.8% (95% CI: 1.4–4.4) (Table 4). The median AUDIT score among all alcohol consumers (AUDIT ≥ 1, n = 419) was 8.0 (IQR: 3.0 – 13.0), with scores ranging from 1 to 30. Consistently, it increased significantly across categories (*p* < 0.001): 3.0 (IQR: 1.0 – 4.0) for low-risk use, 9.0 (IQR: 8.0 – 11.0) for harmful use, and 18.0 (IQR: 14.0 – 22.0) for alcohol dependence (Table 4).

**Table 4:** Levels of alcohol consumption in the general population.

Variables	Prevalence of alcohol consumption levels		*p*	AUDIT	*p*
	n (%)	95% CI		Median (IQR)	
**Alcohol consumption level**					
Non-use (AUDIT = 0)	12 (2.8)	1.4 – 4.4	< 0.001	0	< 0.001
Low-risk use (AUDIT = 1–6)	185 (42.9)	38.3 – 47.3		3.0 (1.0 – 4.0)	
Harmful use (AUDIT = 7–12)	124 (28.8)	24.6 – 32.9		9.0 (8.0 – 11.0)	
Alcohol dependence (AUDIT ≥ 13)	110 (25.5)	21.6 – 29.7		18.0 (14.0 – 22.0)	
**Use of alcohol**					
Non-abuse (AUDIT ≤ 6)	197 (45.7)	40.1 – 50.1	0.074	2.0 (1.0 – 4.0)	< 0.001
Abuse (AUDIT ≥ 7)	234 (54.3)	49.4 – 58.7		12.0 (9.0 – 17.3)	

AUDIT: Alcohol Use Disorder Identification Test score; it represents the median score of the participants in each group. IQR: Interquartile range.

n (%): number of participants (n) and prevalence (%); 95% CI: 95% confidence interval associated with the prevalence.

Overall, the prevalence of alcohol abuse (AUDIT ≥ 7) in the study population was 54.3% (95% CI: 49.4–58.7), whereas it was 45.7% (95% CI: 40.1–50.1) for the non-abuse of alcohol (AUDIT ≤ 6). However, a significant difference in terms of alcohol consumption intensity was recorded between these groups (*p* < 0.001), with median AUDIT scores of 12.0 (IQR: 9.0 – 17.3) among alcohol abuse consumers compared to 2.0 (IQR: 1.0 – 4.0) among non-abuse alcohol consumers (Table 4).

#### Prevalence of alcohol abuse consumption according to socio-demographic criteria.

Community-level analysis revealed significant differences (*p* < 0.001) in both the prevalence and alcohol consumption intensity. The Carrière and Mock Sud communities displayed the highest prevalence rates of alcohol abuse, 69.5% (95% CI: 61.1–77.1) and 67.1% (95% CI: 56.1–76.8), respectively, while Baloua and Mock Centre exhibited much lower prevalence, around 40%. This disparity was also reflected in the median alcohol consumption intensity (AUDIT score), which was higher in Carrière (10.0 (IQR: 4.0 – 17.0)) and Mock Sud (9.0 (IQR: 5.8 - 13.0)), corresponding to alcohol abuse (AUDIT ≥ 7). In contrast, lower scores in Baloua (5.0 (IQR: 2.0 – 11.0)) and Mock Centre (4.0 (IQR: 2.0 – 10.0)), consistent with non-abuse alcohol consumption (AUDIT ≤ 6) (Table 5).

**Table 5:** Association between alcohol consumption levels and sociodemographic criteria.

Variable	N = 431	Prevalence of “alcohol abuse”		*p*	AUDIT	*p*
		n (%)	95% CI		Median (IQR)	
**Community**						
Baloua	115	47 (40.9) ^**c, ms**^	32.2 – 50.4	< 0.001	5.0 (2.0 – 11.0) ^**c,ms**^	< 0.001
Carrière	131	91 (69.5) ^**b**^	61.1 – 77.1		10.0 (4.0 – 17.0) ^**b,mc**^	
Mock Centre	103	41 (39.8) ^**c, ms**^	31.1 – 49.5		4.0 (2.0 – 10.0) ^**c,ms**^	
Mock Sud	82	55 (67.1) ^**b, mc**^	56.1 – 76.8		9.0 (5.8 - 13.0) ^**b,mc**^	
**Sex**						
Male	232	161 (69.4)	63.8 – 75.3	< 0.001	11.0 (5.0 – 16.0)	< 0.001
Female	199	73 (36.7)	30.0 – 43.3		4.0 (2.0 – 9.0)	
**Age groups (years)**						
18 – 39	196	98 (50.0) ^**β**^	42.9 – 57.1	0.032	6.5 (2.0 – 12.0)	0.056
40 – 59	162	101 (62.3) ^**α, γ**^	54.3 – 69.8		9.0 (4.0 – 13.0)	
≥ 60	73	35 (47.9) ^**β**^	37.0 – 60.3		6.0 (3.0 – 12.5)	

N: total sample size; n (%): cases of alcohol abuse (n) and prevalence (%); 95% CI: 95% confidence interval associated with prevalence; AUDIT: Alcohol Use Disorder Identification Test score; it represents the median score of the participants in each group. IQR: interquartile range.

b: significantly different from Baloua; c: significantly different from Carrière; mc: significantly different from Mock Centre; ms: significantly different from Mock Sud. α: significantly different from 18–39 years; β: significantly different from 40–59 years; γ: significantly different from ≥ 60 years

Concerning sex, the prevalence of alcohol abuse consumption was higher in men (69.4%) than in women (36.7%) (*p* < 0.001), with an AUDIT score of 11.0 (IQR: 5.0 – 16.0) among men and 4.0 (IQR: 2.0 – 9.0) for women, categorizing men as alcohol abusers and women as non-abusers (Table 5).

Depending on age groups, the prevalence of alcohol abuse was higher among participants aged 40–59 years (62.3% (95% CI: 54.3–69.8)) than among others (*p* = 0.032). The AUDIT score did not differ significantly (*p* = 0.056) but was confirmed as “alcohol abuse” in the 40–59 age group and “non-alcohol abuse” in other age groups (Table 5).

#### Prevalence of schistosomiasis and alcohol abuse comorbidity.

Schistosomiasis and alcohol abuse comorbidity was defined as the concomitant presence of *S. mansoni* infection and alcohol abuse consumption (AUDIT ≥ 7) within the same individual. Prevalence of the comorbidity in the general population

As shown in Table 6, the overall prevalence of schistosomiasis–alcohol comorbidity was 17.4% (95% CI: 13.9–21.1). The mean parasitic burden was 95.4 EPG (95% CI: 66.8 – 136.4) within co-morbid participants, corresponding to a light *S. mansoni* infection intensity. The AUDIT score among those participants was 12.0 (IQR: 9.0 – 16.0), reflecting harmful drinking.

**Table 6:** Prevalence of schistosomiasis mansoni and alcohol abuse comorbidity in the global population and according to sociodemographic criteria.

Variable		Prevalence of comorbidity			Intensity of alcohol abuse		Severity of *S. mansoni* infection		
	N	n (%)	95% CI	*p*	AUDIT (IQR)	*p*	n_kk_	Mean EPG (95% CI)	*p*
**Community**									
Baloua	115	27 (23.5)^**mc**^	16.5 – 31.3	0.001	12.0 (8.0 – 15.0)	0.426	16	134.0 (69.4 – 258.9)	0.103
Carrière	131	31 (23.7)^**mc**^	16.0 – 31.3		12.0 (9.0 – 21.0)		15	71.1 (38.9 – 129.8)	
Mock Centre	103	8 (7.8)^**b,c**^	2.9 – 13.6		9.0 (8.0 – 12.3)		6	64.0 (35.1 – 116.6)	
Mock Sud	82	9 (11.0)	4.9 – 18.3		11.0 (9.0 – 16.5)		2	190.5 (38.6 – 940.3)	
**Sex**									
Male	232	47 (20.3)	15.1 – 25.4	0.091	12.0 (9.0 – 18.0)	0.232	21	72.6 (43.4 – 121.3)	0.020
Female	199	28 (14.1)	9.5 – 19.1		9.5 (9.0 – 14.8)		18	131.3 (79.5 – 216.8)	
**Age groups (years)**									
18 - 39	196	43 (21.9) ^**γ**^	16.5 – 27.8	0.026	12.0 (9.0 – 20.0)	0.143	18	119.2 (63.9 – 222.2)	0.483
40 - 59	162	26 (16.0)	10.2 – 22.1		9.5 (8.0 – 15.0)		16	83.4 (49.9 – 139.6)	
≥ 60	73	6 (8.2)^**α**^	2.5 – 14.7		9.0 (7.0 – 17.5)		5	65.9 (20.1 – 215.6)	
**Global population**	431	75 (17.4)	13.9 – 21.1	–	12.0 (9.0 – 16.0)	–	39	95.4 (66.8 – 136.4)	–

N: total sample size; n (%): cases of schistosomiasis and alcohol abuse comorbidity (n) and prevalence (%); 95% CI: 95% confidence interval associated with the prevalence; AUDIT: Alcohol Use Disorder Identification Test score; it represents the median score of the participants in each group; IQR: Interquartile range; nₖₖ: schistosomiasis mansoni cases diagnosed by Kato-Katz; EPG: Egg per gram of stool.

b: significantly different from Baloua; c: significantly different from Carrière; mc: significantly different from Mock Centre.

α: significantly different from the 18–39 years; γ: significantly different from the ≥ 60 years.

#### Prevalence of the comorbidity according to communities.

At the community level, a significant variation of the comorbidity prevalence was recorded across communities (*p* = 0.001). Baloua and Carrière displayed higher prevalence of 23.5% (95% CI: 16.5–31.3) and 23.7% (95% CI: 16.0–31.3), respectively, whereas Mock Centre and Mock Sud exhibited lower prevalence of 7.8% (95% CI: 2.9–13.6) and 11.0% (95% CI: 4.9–18.3), respectively. Regarding the intensity of alcohol consumption, no significant difference was observed between communities (*p* = 0.426), and the AUDIT score indicated harmful drinking among co-morbid participants from any communities. No significant variability of *S. mansoni* infection intensity was recorded across communities (*p* = 0.103). However, a moderate intensity of infection at Mock Sud (190.5 EPG (95% CI: 38.6 – 940.3)) and Baloua (134.0 EPG 95% CI: 69.4 – 258.9)) and a light intensity of infection at Carrière (71.1 EPG (95% CI: 38.9 – 129.8)) and Mock Centre (64.0 EPG (95% CI: 35.1 – 116.6)) (Table 6).

#### Prevalence of the comorbidity according to sex.

The prevalence of schistosomiasis mansoni and alcohol abuse comorbidity was slightly higher among men, 20.3% (95% CI: 15.1–25.4), compared to 14.1% (95% CI: 9.5–19.1) among women, without a statistically significant variation (*p* = 0.091). Although the intensity of alcohol consumption did not differ significantly between sexes (*p* = 0.232), men had an AUDIT score of 12.0 (IQR: 9.0 – 18.0) compared to 9.5 (IQR: 9.0 – 14.8) for women, both indicating harmful drinking. Regarding the severity of *S. mansoni* infection, women displayed moderate intensity of infection (131.3 EPG (95% CI: 79.5 – 216.8)) compared to light intensity of infection for men (72.6 EPG (95% CI: 43.4 – 121.3)) (*p* = 0.020) (Table 6).

#### Prevalence of the comorbidity according to age groups.

The prevalence of schistosomiasis–alcohol comorbidity significantly decreased with age (*p* = 0.026). Participants aged 18–39 years showed the highest prevalence at 21.9% (95% CI: 16.5–27.8), followed by those aged 40–59 years at 16.0% (95% CI: 10.2–22.1), while the ≥ 60 years group had the lowest prevalence at 8.2% (95% CI: 2.5–14.7). Alcohol consumption levels were generally similar across age groups (*p* = 0.143), although slightly higher among young adults (12.0 (IQR: 9.0 – 20.0)), compared to middle-aged (9.5 (IQR: 8.0 – 15.0)) and older adults (9.0 (IQR: 7.0 – 17.5)), with all three groups reflecting harmful drinking behaviors. Regarding infection severity, a moderate intensity of infection (119.2 EPG (95% CI: 63.9 – 222.2)) was observed in the 18–39 years age group, while a light intensity of infection (65.9 EPG (95% CI: 20.1 – 215.6)) was recorded in older age groups (Table 6).

#### Interaction between *Schistosoma mansoni* infection intensity and alcohol use status.

No significant association was established between alcohol consumption level and *S. mansoni* infection intensity. Among infected participants, the mean egg count in non-alcohol abusers (106.0 EPG, 95% CI: 74.4 – 150.9) was not statistically different from that of alcohol abusers (95.4 EPG, 95% CI: 66.8 – 136.4) (p = 0.488). Likewise, the severity of alcohol consumption did not differ significantly according to *S. mansoni* infection status. The median AUDIT score was 8 (IQR: 3.0 – 13.0) among uninfected participants and 7 (IQR: 2.5 – 12.0) among infected participants (p = 0.170).

### Factors associated with *Schistosoma mansoni* infection

#### Sociodemographic factors of *Schistosoma mansoni* infection.

Among the sociodemographic factors analyzed, young adults (18 – 39 years) were 2.8 times more at risk of *S. mansoni* infection (OR = 2.84, 95% CI: 1.52 – 5.29, *p* = 0.001). Similarly, residents of the Baloua neighborhood had a 2.5-fold higher risk (OR = 2.51, 95% CI: 1.62 – 3.90, *p* < 0.001), while living within 500 meters of a transmission site was associated with a twofold increase in risk (OR = 2.09, 95% CI: 1.03 – 4.24, *p* = 0.041). In contrast, living in Mock Centre was significantly associated with a lower risk of infection (OR = 0.49, 95% CI: 0.29 – 0.82, *p* = 0.006) (Table 7).

**Table 7:** Association between sociodemographic factors and *Schistosoma mansoni* infection.

Variables	*S. mansoni* positive	*S. mansoni* negative	OR (95% CI)	χ²; *p*
	N = 149; n (%)	N = 282; n (%)		
**Sex**				
Male	72 (31.0)	160 (69.0)	0.71 (0.47-1.06)	2.778; 0.096
Female	77 (38.7)	122 (61.3)	1	
**Age groups (years)**				
18-39	87 (44.4)	109 (55.6)	2.84 (1.52-5.29)	10.843; 0.001
40-59	46 (28.4)	116 (71.6)	1.41 (0.73-2.70)	1.081; 0.298
≥ 60	16 (21.9)	57 (78.1)	1	
**Level of education**				
None/primary	61 (33.7)	120 (66.3)	0.93 (0.62-1.40)	0.104; 0.747
Secondary/higher	88 (35.2)	162 (64.8)	1	
**Main occupation** ^1^				
Farmer/Breeder	98 (36.8)	168 (63.2)	1.30 (0.86-1.97)	1.585; 0.208
Salesperson	7 (33.3)	14 (66.7)	0.94 (0.37-2.39)	0.015; 0.903
Housewife	19 (31.1)	42 (68.9)	0.83 (0.46-1.49)	0.368; 0.544
Student	7 (31.8)	15 (68.2)	0.87 (0.35-2.20)	0.078; 0.780
Technician	10 (30.3)	23 (69.7)	0.81 (0.37-1.75)	0.288; 0.592
Unemployed/Retired	2 (20.0)	8 (80.0)	0.46 (0.09-2.22)	0.961; 0.327
**Community** ^**2**^				
Baloua	58 (50.4)	57 (49.6)	2.51 (1.62-3.90)	17.452; < 0.001
Carrière	44 (33.6)	87 (66.4)	0.93 (0.60-1.44)	0.080; 0.777
Mock-Centre	24 (23.3)	79 (76.7)	0.49 (0.29-0.82)	7.600; 0.006
Mock-Sud	23 (28.0)	59 (72.0)	0.69 (0.40-1.17)	1.904; 0.168
**Household–transmission site distance (m)**				
< 500	111 (36.5)	193 (63.5)	2.09 (1.03-4.24)	4.186; 0.041
500 – 1000	27 (35.5)	49 (64.5)	2.00 (0.88-5.53)	2.785; 0.095
> 1000	11 (21.6)	40 (78.4)	1	

1 For each occupation, the five others were considered as reference.

2 For each community, the three others were considered as the reference.

#### Behavioral factors associated with *Schistosoma mansoni* infection.

Among the lifestyle-related factors examined, the regular performance of household chores at the transmission site was the only variable significantly associated with *S. mansoni* infection, doubling the risk of infection (OR = 2.09, 95% CI: 1.37 – 3.19, *p* = 0.001) (Table 8).

**Table 8:** Association between behavioral characteristics and *Schistosoma mansoni* infection.

Variables	*S. mansoni* positive	*S. mansoni* negative	OR (95% CI)	χ²; *p*
	N = 149; n (%)	N = 282; n (%)		
**Alcohol abuse**				
Yes	75 (32.1)	159 (67.9)	0.78 (0.52-1.16)	1.437; 0.231
No	74 (37.6)	123 (62.4)	1	
**Knowledge of schistosomiasis**				
Yes	133 (35.4)	243 (64.6)	1.33 (0.71-2.47)	0.837; 0.360
No	16 (29.1)	39 (70.9)	1	
**Previous treatment with PZQ**				
Never	92 (37.4)	154 (62.6)	1	
More than 12 months	42 (32.8)	86 (67.2)	0.81 (0.52-1.28)	0.769; 0.381
Less than 12 months	15 (26.3)	42 (73.7)	0.59 (0.31-1.13)	2.455; 0.117
**Housework practices at the transmission site**				
Never	63 (28.0)	162 (72.0)	1	
Irregularly	12 (29.3)	29 (70.7)	1.06 (0.51-2.21)	0.027; 0.868
Regularly	74 (44.8)	91 (55.2)	2.09 (1.37-3.19)	11.689; 0.001
**Regular fishing practice**				
Yes	11 (42.3)	15 (57.7)	1.41 (0.63-3.17)	0.732; 0.392
No	138 (34.1)	267 (65.9)	1	
**Itching (water contact)**				
Never	88 (34.2)	169 (65.8)	1	
Irregularly	46 (33.8)	90 (66.2)	0.98 (0.63-1.52)	0.006; 0.934
Regularly	15 (39.5)	23 (60.5)	1.25 (0.62-2.52)	0.398; 0.528
**Skin rash (water contact)**				
Never	117 (36.2)	206 (63.8)	1	
Irregularly	27 (27.0)	73 (73.0)	0.65 (0.39-1.07)	2.869; 0.090
Regularly	5 (62.5)	3 (37.5)	2.93 (0.68-12.50)	2.119; 0.145

#### Multivariate analysis of *Schistosoma mansoni* infection and its associated factors.

The binary logistic regression model revealed three factors independently associated with a higher risk of *S. mansoni* infection. Young adults (18–39 years) had a twofold higher risk of infection (AOR = 2.01, 95% CI: 1.31 – 3.08, *p* = 0.001), while residing in the Baloua neighborhood was linked to a 2.6-fold increase in risk (AOR = 2.64, 95% CI: 1.67 – 4.16, *p* < 0.001). Additionally, regular performance of household chores at the transmission site increased the likelihood of infection by 1.8 times (AOR = 1.83, 95% CI: 1.19–2.82, *p* = 0.006) (Table 9).

**Table 9:** Factors independently associated with *Schistosoma mansoni* infection.

Variables		Crude odds ratio		Adjusted odds ratio	
	*S. mansoni* positive, n (%)	COR (95% CI)	*p*	AOR (95% CI)	*p*
**Age groups (years)** ^1^					
18-39 ans	87 (44.4)	2.03 (1.32-3.11)	0.001	2.01 (1.31-3.08)	0.001
**Community** ^2^					
Baloua	58 (50.4)	2.49 (1.53-4.08)	< 0.001	2.64 (1.67-4.16)	< 0.001
Mock-Centre	24 (23.3)	0.77 (0.44-1.35)	0.372		
**Household–transmission site distance (m)** ^3^					
< 500 m	111 (36.5)	1.26 (0.77-2.05)	0.352	/	/
**Housework practices at the transmission site** ^4^					
Regularly	74 (44.8)	1.70 (1.09-2.65)	0.019	1.83 (1.19-2.82)	0.006

^1^The other two age groups (40–59 years, ≥ 60 years) were considered as the reference.

^2^For each community, the three others (Carriere, Mock Sud, Baloua or Mock Centre) were considered as the reference.

^3^The other two distance groups (500–1000 m, > 1000 m) were considered as the reference.

^4^Irregular practice of household chores at the transmission site was considered as the reference.

### Factors associated with alcohol abuse

#### Sociodemographic factors associated with alcohol abuse.

Among the sociodemographic factors analyzed, men were found to be at significantly higher risk of alcohol abuse, with a risk 3.9 times greater than that of women (OR = 3.91, 95% CI: 2.62 – 5.84, *p* < 0.001). Concerning age groups, only middle adults aged 40–59 years showed a significantly increased risk, being approximately 1.7 times more likely to alcohol abuse compared to other age categories (OR = 1.69, 95% CI: 1.13 – 2.52, *p* = 0.009) (Table 10).

**Table 10:** Association between sociodemographic factors and alcohol abuse.

Variables	Alcohol abuse	Non-abuse of alcohol	OR (95% CI)	χ²; *p*
	N = 234; n (%)	N = 197; n (%)		
**Sex**				
Male	161 (69.4)	71 (30.6)	3.91 (2.62 – 5.84)	46.193; < 0.001
Female	73 (36.7)	126 (63.3)	1	
**Age groups (years)** ^^1^^				
18-39	98 (50.0)	98 (50.0)	0.72 (0.49 – 1.06)	2.669; 0.102
40-59	101 (62.3)	61 (37.7)	1.69 (1.13 – 2.52)	6.274; 0.009
≥ 60	35 (47.9)	38 (52.1)	0.73 (0.44 – 1.21)	1.427; 0.232
**Level of education**				
None/primary	94 (51.9)	87 (48.1)	0.84 (0.57 -1.24)	0.698; 0.403
Secondary/higher	140 (56.0)	110 (44.0)	1	
**Main occupation** ^2^				
Farmer/Breeder	158 (59.4)	108 (40.6)	1.71 (1.15 – 2.53)	7.300; < 0.007
Salesperson	11 (52.4)	10 (47.6)	0.92 (0.38 – 2.22)	0.033; 0.857
Housewife	21 (34.4)	40 (65.6)	0.38 (0.22 – 0.68)	11.301; < 0.001
Student	6 (27.3)	16 (72.7)	0.29 (0.11 – 0.77)	6.820; < 0.009
Technician	24 (72.7)	9 (27.3)	2.38 (1.08 – 5.26)	4.894; 0.027
Unemployed/Retired	3 (30.0)	7 (70.0)	0.35 (0.09 – 1.38)	2.434; 0.119
**Community** ^3^				
Baloua	47 (40.9)	68 (59.1)	0.47 (0.30 – 0.73)	11.388; < 0.001
Carrière	91 (69.5)	40 (30.5)	2.49 (1.61 – 3.86)	17.461; < 0.001
Mock-Centre	41 (39.8)	62 (60.2)	0.46 (0.2 – 0.72)	11.446; < 0.001
Mock-Sud	55 (67.1)	27 (32.9)	1.93 (1.16 – 3.20)	6.666; < 0.001

^1^For each age group, the other two age groups were considered as the reference.

^2^For each occupation, the five others were considered as a reference.

^3^For each community, the other three were considered as the reference.

Educational level did not appear to influence alcohol abuse (OR = 0.84, 95% CI: 0.57 – 1.24, *p* = 0.403). In contrast, occupational status had a significant impact: farmers and livestock breeders were 1.7 times more likely to experience alcohol abuse (OR = 1.71, 95% CI: 1.15 – 2.53, *p* = 0.007), and technicians had a 2.3-fold higher risk (OR = 2.38, 95% CI: 1.08 – 5.26, *p* = 0.027). Conversely, housewives showed an approximately 60% lower risk (OR = 0.38, 95% CI: 0.22 – 0.68, *p* < 0.001), while students showed a reduction in risk of alcohol abuse exceeding 70% (OR = 0.29, 95% CI: 0.11 – 0.77, *p* = 0.009) (Table 10).

Moreover, the community of residence was significantly associated with alcohol abuse. Participants living in Carrière and Mock-Sud had 2.5-fold (OR = 2.49, 95% CI: 1.61 – 3.86, *p* < 0.001) and 1.9-fold (OR = 1.93, 95% CI: 1.16 – 3.20, *p* < 0.001) higher risks, respectively, compared to residents of other communities. Conversely, those residing in Baloua and Mock-Centre showed more than a 50% reduction in risk (Baloua: OR = 0.47, 95% CI: 0.30 – 0.73, *p* < 0.001; Mock-Centre: OR = 0.46, 95% CI: 0.29 – 0.72, *p* < 0.001) (Table 10).

#### Consumption of alcoholic beverage types and risk of alcohol abuse.

All types of alcoholic beverages analyzed were significantly associated with alcohol abuse, indicating that regular consumption, regardless of the beverage type, constitutes a high-risk behavior. Nonetheless, certain beverages showed a strong association with alcohol abuse. Adulterated whisky exhibited the highest association, increasing the risk of abuse by 37.9 times (OR = 37.93, 95% CI: 5.16 – 278.80, *p* < 0.001), followed by beer and palm wine, which raised the risk by 9.1-fold (OR = 9.14, 95% CI: 5.54 – 15.10, *p* < 0.001) and 5.7-fold (OR = 5.74, 95% CI: 3.67 – 8.98, *p* < 0.001), respectively. The traditional beverage “cha” was also linked to alcohol abuse, although to a lesser degree, with a 2.3-fold increase in risk (OR = 2.32, 95% CI: 1.19 – 4.54, *p* = 0.011). Moreover, regular consumption of wine, spirits, and “odontol” was consistently associated with abuse, as all regular consumers were affected; however, the small sample size for these beverages limits the strength of this finding (Table 11).

**Table 11:** Association between the consumption of alcoholic beverages and alcohol abuse.

Variables	Alcohol abuse	Non-abuse of alcohol	OR (95% CI)	χ²; *p*
	N = 185; n (%)	N = 234; n (%)		
**Adulterated whisky**				
Regularly	40 (97.6)	1 (2.4)	37.93 (5.16 – 278.80)	32.070; < 0.001
Irregularly	194 (51.3)	184 (48.7)	1	
**Palm wine**				
Regularly	136 (79.1)	36 (20.9)	5.74 (3.67 – 8.98)	63.812; < 0.001
Irregularly	98 (39.7)	149 (60.3)	1	
**Beer**				
Regularly	135 (84.9)	24 (15.1)	9.14 (5.54 – 15.10)	87.745; < 0.001
Irregularly	99 (38.1)	161 (61.9)	1	
“**Cha**”				
Regularly	35 (72.9)	13 (27.1)	2.32 (1.19 – 4.54)	6.406; 0.011
Irregularly	199 (53.6)	172 (46.4)	1	
**Wine**				
Regularly	8 (100.0)	0 (0.0)	1.81 (1.66 – 1.98)	6.448; 0.011
Irregularly	226 (55.0)	185 (45.0)	1	
**Spirits**				
Regularly	9 (100.0)	0 (0.0)	1.82 (1.66 – 1.98)	7.272; 0.007
Irregularly	225 (54.9)	185 (45.1)	1	
“**Odontol**”				
Regularly	12 (100.0)	0 (0.0)	1.83 (1.67 – 2.00)	9.767; 0.002
Irregularly	222 (54.5)	185 (45.5)	1	

Palm wine, “Cha”, and “Odontol” are traditional beverages. “Cha” is a maize turbid beer, and “Odontol” is made from palm wine, sugar, and a tree bark.

#### Drinking motivations associated with alcohol abuse.

Participants who reported drinking to “enjoy their evening” or to “make social activities more fun” had a 4.3-fold (OR = 4.31, 95% CI: 2.85 – 6.50, *p* < 0.001) and 4.5-fold (OR = 4.50, 95% CI: 2.98 – 6.80, *p* < 0.001) higher risk of alcohol abuse, respectively. Motivations such as “fitting in with peers” and “seeking appreciation” were even more pronounced, increasing the risk by 5.3-fold (OR = 5.32, 95% CI: 2.88 – 9.81, *p* < 0.001) and 6.4-fold (OR = 6.40, 95% CI: 2.20 – 18.55, *p* < 0.001). Similarly, drinking to “cope with nervousness or depression,” to “lift mood when feeling low,” or to “forget worries” significantly raised the likelihood of abuse, with risks increased by 3.9-fold (OR = 3.89, 95% CI: 1.57 – 9.63, *p* = 0.002), 10.3-fold (OR = 10.31, 95% CI: 3.11 – 34.15, *p* < 0.001), and 5.7-fold (OR = 5.65, 95% CI: 1.93 – 16.51, *p* < 0.001), respectively. In contrast, drinking for the “pleasure of drunkenness” was not significantly associated with alcohol abuse (OR = 2.04, 95% CI: 0.77 – 5.37, *p* = 0.140) (Table 12).

**Table 12:** Association between drinking motivations and alcohol abuse.

Variables	Alcohol abuse	Non-abuse of alcohol	OR (95% CI)	χ²; *p*
	N = 185; n (%)	N = 234; n (%)		
**Enjoying the evening**				
Regularly	159 (72.3)	61 (27.7)	4.31 (2.85 – 6.50)	50.683; < 0.001
Irregularly	75 (37.7)	124 (62.3)	1	
**Making social activities more fun**				
Regularly	160 (72.7)	60 (27.3)	4.50 (2.98 – 6.80)	53.527; < 0.001
Irregularly	74 (37.2)	125 (62.8)	1	
**Fitting in with peers**				
Regularly	71 (83.5)	14 (16.5)	5.32 (2.88 – 9.81)	33.138; < 0.001
Irregularly	163 (48.8)	171 (51.2)	1	
**Seeking appreciation**				
Regularly	29 (87.9)	4 (12.1)	6.40 (2.20 – 18.55)	14.905; < 0.001
Irregularly	205 (53.1)	181 (46.9)	1	
**Coping with nervousness or depression**				
Regularly	27 (81.8)	6 (18.2)	3.89 (1.57 – 9.63)	9.798; 0.002
Irregularly	207 (53.6)	179 (46.4)	1	
**Lifting mood when feeling low**				
Regularly	34 (91.9)	3 (8.1)	10.31 (3.11 – 34.15)	21.383; < 0.001
Irregularly	200 (52.4)	182 (47.6)	1	
**Forgetting worries**				
Regularly	26 (86.7)	4 (13.3)	5.65 (1.93 – 16.51)	12.447; < 0.001
Irregularly	208 (53.5)	181 (46.5)	1	
**Pleasure of drunkenness**				
Regularly	15 (71.4)	6 (28.6)	2.04 (0.77 – 5.37)	2.177; 0.140
Irregularly	219 (55.0)	179 (45.0)	1	

The grouped analysis of drinking motivations highlights the dominant influence of psychological, social, and conformity factors in driving alcohol abuse. Coping motives, related to managing stress or regulating negative emotions, had the strongest effect, increasing the risk of abuse by 6.28 times (OR = 6.28, 95% CI: 3.28 – 12.00, *p* < 0.001). Conformity motives followed, as drinking to respond to social pressure or a desire for acceptance raised the risk of abuse by 5.90 times (OR = 5.90, 95% CI: 3.39 – 10.26, *p* < 0.001). Social motives, involving drinking to enhance or facilitate social interactions, were also significant, multiplying the risk of abuse by 4.15 (OR = 4.15, 95% CI: 2.74 – 6.29, *p* < 0.001). In contrast, enhancement motives, oriented toward seeking positive sensations or amplifying pleasure, were not significantly associated with alcohol abuse (OR = 2.04, 95% CI: 0.77 – 5.37, *p* = 0.140) (Table 13).

**Table 13:** Association between types of motivation and alcohol abuse.

Variables	Alcohol abuse	Non-abuse of alcohol	OR (95% CI)	χ²; *p*
	N = 185; n (%)	N = 234; n (%)		
**Conformity motives**				
Regularly	91 (83.5)	18 (16.5)	5.90 (3.39 – 10.26)	45.642; < 0.001
Irregularly	143 (46.1)	167 (53.9)	1	
**Social motives**				
Regularly	174 (69.6)	76 (30.4)	4.15 (2.74 – 6.29)	47.543; < 0.001
Irregularly	60 (35.5)	109 (64.5)	1	
**Coping motives**				
Regularly	71 (85.5)	12 (14.5)	6.28 (3.28 – 12.00)	37.013; < 0.001
Irregularly	163 (48.5)	173 (51.3)	1	
**Enhancement motives**				
Regularly	15 (71.4)	6 (28.6)	2.04 (0.77 – 5.37)	2.177; 0.140
Irregularly	219 (55.0)	179 (45.0)	1	

Motives were classified according to Cooper’s four-factor model.

#### Multivariate analysis of alcohol abuse and its associated factors.

Among sociodemographic characteristics, being male was a significant predictor, with a 2.4-fold increased risk of abuse (AOR = 2.48, 95% CI: 1.39 – 4.41, *p* = 0.002). Conversely, living in Mock-Centre had a significantly lower risk, approximately 80% less than other communities (AOR = 0.22, 95% CI: 0.09 – 0.51, *p* < 0.001), while no significant associations were observed for other localities after adjustment (Table 14).

**Table 14:** Factors independently associated with alcohol abuse.

Variables		Crude odds ratio		Adjusted odds ratio	
	Alcohol abuse n (%)	COR (95% CI)	*p*	AOR (95% CI)	*p*
**Sex**					
Male	161 (69.4)	2.48 (1.39 – 4.44)	0.002	2.48 (1.39 -4.41)	0.002
**Age groups (years)** ^1^					
40-59 years	101 (62.3)	1.70 (0.95 – 3.04)	0.074	1.70 (0.96 – 3.03)	0.068
**Main occupation** ^2^					
Farmer/Breeder	158 (59.4)	0.76 (0.32 – 1.81)	0.545	0.78 (0.37 – 1.64)	0.517
Housewife	21 (34.4)	1.27 (0.44 – 3.63)	0.652	1.30 (0.49 – 3.41)	0.594
Student	6 (27.3)	0.94 (0.24 – 3.63)	0.935	/	/
Technician	24 (72.7)	1.60 (0.43 – 5.89)	0.473	1.65 (0.49 – 5.58)	0.415
**Community** ^3^					
Baloua	47 (40.9)	0.59 (0.26 – 1.31)	0.197	0.58 (0.26 – 1.29)	0.187
Carrière	91 (69.5)	0.90 (0.41 – 1.98)	0.804	0.90 (0.41 – 1.98)	0.803
Mock-Centre	41 (39.8)	0.22 (0.09 – 0.51)	< 0.001	0.22 (0.09 – 0.51)	< 0.001
**Adulterated whisky** ^4^					
Regularly	40 (97.6)	26.84 (2.76 – 261.17)	0.005	26.84 (2.77 – 230.20)	0.005
**Palm wine** ^4^					
Regularly	136 (79.1)	1.54 (0.82 – 2.90)	0.174	1.56 (0.84 – 2.91)	0.159
**Beer** ^4^					
Regularly	135 (84.9)	5.81 (3.05 – 11.90)	< 0.001	5.81 (3.04 – 11.07)	< 0.001
**“Cha**”^4^					
Regularly	35 (72.9)	1.10 (0.39 – 3.07)	0.851	/	/
**Conformity motives** ^5^					
Regularly	91 (83.5)	4.82 (2.40 – 9.68)	< 0.001	4.80 (2.39 – 9.63)	< 0.001
**Social motives** ^5^					
Regularly	174 (69.6)	2.33 (1.35 – 4.04)	0.002	2.33 (1.35 – 4.03)	0.002
**Coping motives** ^5^					
Regularly	71 (85.5)	3.06 (1.39 – 6.73)	0.005	3.05 (1.39 – 6.71)	0.005

“Cha”, a traditional beverage, is a maize turbid beer.

^1^The other two age groups (18 – 39 years and ≥ 60 years) were considered as the reference.

^2^For each main occupation, five others were considered as a reference.

^3^For each community, the other three were considered as the reference.

^4^Irregular consumption of the beverage of interest was considered as the reference.

^5^Irregular consumption associated with the motivation of interest was considered as the reference

Regarding drinking habits, the strongest determinant of alcohol abuse was the regular consumption of adulterated whisky, which increased the risk 26-fold (AOR = 26.84, 95% CI: 2.77–230.20, *p* = 0.005), followed by beer consumption (AOR = 5.81, 95% CI: 3.04 – 11.07, *p* < 0.001). Among motivational factors, conformity motives had a pronounced influence, raising the likelihood of abuse by 4.8 times (AOR = 4.80, 95% CI: 2.39 – 9.63, *p* < 0.001). Coping motives were also significantly associated, tripling the risk (AOR = 3.05, 95% CI: 1.39 – 6.71, *p* = 0.005), while social motives contributed to a lesser extent, increasing the risk by 2.3-fold (AOR = 2.33, 95% CI: 1.35–4.03, *p* = 0.002) (Table 14).

### Factors associated with *Schistosoma mansoni* infection and alcohol abuse comorbidity

Community of residence emerged as the strongest predictive factor. Residents of Baloua had nearly a fivefold higher risk of comorbidity compared to other communities (AOR = 4.99, 95% CI: 2.36 – 10.56, p < 0.001), followed by those living in Carrière, whose risk was 2.3 times higher (AOR = 2.37, 95% CI: 1.19 – 4.70, p = 0.013) (Table 15).

**Table 15:** Factors independently associated with *Schistosoma mansoni* infection and alcohol abuse comorbidity.

Variable		Crude odds ratio		Adjusted odds ratio	
	Comorbidity n (%)	COR (95% CI)	*p*	AOR (95% CI)	*p*
**Age groups (years)** ^1^					
18 – 39	43 (21.9)	1.91 (1.03 – 3.55)	0.039	1.88 (1.02 – 3.46)	0.042
> 60	6 (8.2)	0.63 (0.23 – 1.68)	0.359	0.63 (0.24 – 1.69)	0.366
**Main occupation** ^2^					
Farmer/Breeder	55 (20.7)	1.25 (0.65 – 2.38)	0.498	1.22 (0.65 – 2.32)	0.525
**Community** ^3^					
Baloua	27 (23.5)	5.50 (2.17 – 13.91)	< 0.001	4.99 (2.36 – 10.56)	< 0.001
Carrière	31 (23.7)	2.59 (1.10 – 6.08)	0.028	2.37 (1.19 – 4.70)	0.013
Mock Centre	8 (7.8)	1.20 (0.41 – 3.46)	0.736	/	/
**Household–transmission site distance (m)** ^4^					
< 500 m	60 (19.7)	1.22 (0.61 – 2.41)	0.566	1.19 (0.60 – 2.36)	0.601
**Housework practices at the transmission site** ^5^					
Never	31 (13.8)	0.62 (0.23 – 1.65)	0.344	0.71 (0.40 – 1.28)	0.264
Regularly	37 (22.4)	0.85 (0.31 – 2.28)	0.748	/	/
**Palm wine** ^6^					
Regularly	49 (28.5)	2.78 (1.41 – 5.49)	0.003	2.72 (1.39 – 5.34)	0.003
**Beer** ^6^					
Regularly	44 (27.7)	2.00 (1.05 – 3.79)	0.033	2.00 (1.06 – 3.80)	0.032
**Conformity motives** ^7^					
Regularly	29 (26.6)	1.59 (0.87 – 2.89)	0.124	1.59 (0.87 – 2.88)	0.126
**Coping motives** ^7^					
Regularly	22 (26.5)	1.25 (0.64 – 2.41)	0.503	1.25 (0.65 – 2.41)	0.491

^1^For each age group, the other two were considered as the reference.

^2^The other five occupations were considered as a reference.

^3^For each community, the other three were considered as the reference.

^4^The other two distance groups (≥ 500m) were considered as the reference.

^5^Each category of this variable was compared to the other two frequencies combined, which served as the reference group.

^6^Irregular consumption of the beverage of interest was considered as the reference.

^7^Irregular consumption associated with the type of interest motivation was considered as the reference.

Regarding drinking habits, regular consumption of palm wine was a major determinant, increasing the likelihood of comorbidity by 2.7 times (AOR = 2.72, 95% CI: 1.39 – 5.34, p = 0.003). Regular beer consumption was also significantly associated with comorbidity, with a twofold increased risk (AOR = 2.00, 95% CI: 1.06 – 3.80, p = 0.032) (Table 15).

With respect to demographic characteristics, age played a significant role: individuals aged 18–39 years had nearly twice the risk of comorbidity compared to other age groups (AOR = 1.88, 95% CI: 1.02 – 3.46, p = 0.042). In contrast, factors related to the main occupation, distance from the schistosomiasis transmission site, and alcohol consumption motives did not show statistically significant associations (Table 15).

## Discussion

This study documented, for the first time in a semi-rural area of Cameroon, the prevalence of comorbidity between *Schistosoma mansoni* infection and alcohol abuse. Through this original approach, it highlights an interaction that remains little explored in the literature. It provides novel insights into the epidemiological and behavioral dynamics of this dual public health concern.

The prevalence of schistosomiasis mansoni among adults in Makenene, assessed using the Kato-Katz (19.6%) and the POC-CCA (66.1%), resulted in an overall prevalence of 34.6%. The lower concordance between the overall prevalence and that of the POC-CCA sub-sample may reflect the fact that the POC-CCA sub-sample was not fully representative of the entire study population. Furthermore, the high sensitivity of the POC-CCA test may have allowed for the detection of low-intensity infections that were not identified by the KK technique.

The current study provides the first estimate of *S. mansoni* prevalence among adults in Makenene. According to WHO thresholds [1], the observed prevalence indicates a moderate level of endemicity in this population. Previous epidemiological investigations in Makenene have mainly focused on school-aged children and reported prevalence of 82% in 1985 [21], 41% in 2010 [7], and 40.7% in 2019 [8]. Despite the decline in prevalence in 2010, likely reflecting the impact of mass drug administration campaigns with praziquantel targeting school-aged children, a stagnation in prevalence has been observed since 2010. However, the persistence of substantial infection among adults, who are rarely included in preventive chemotherapy campaigns, suggests that this population may constitute an important reservoir sustaining transmission. These observations highlight the need to broaden the scope of current interventions by further integrating adult populations into control strategies, particularly in areas where exposure to transmission sites remains high, to progress toward the WHO targets for the 2021–2030 period aimed at eliminating schistosomiasis as a public health problem [26].

Analysis of *S. mansoni* infection prevalence across the four surveyed communities revealed rates of 23.3%, 28.0%, 33.6%, and 50.4% in Mock Centre, Mock Sud, Carrière, and Baloua, respectively, underscoring heterogeneous transmission dynamics along the same river system (Mock River). Although all communities exhibited active endemicity, these variations highlight the influence of community-specific risk factors. The moderate prevalences recorded in Mock Centre, Mock Sud, and Carrière suggest regular contact with contaminated waters without necessarily reaching conditions conducive to hyperendemic transmission [1]. The river serves as the primary conduit for both parasite eggs and the intermediate snail host (*Biomphalaria* spp.), thereby maintaining the persistence of infection in these areas [19–20]. Community disparities could be explained by several modulatory factors, including the intensity of fecal contamination [27], the distribution and density of vector snails [19–20], as well as human behaviors and aquatic activities that likely differ between communities, thereby shaping exposure risk [28–30]. The particularly high endemicity observed in Baloua, with a prevalence of 50.4%, may be attributed to a combination of ecological and socio-economic conditions unique to this community. Its swampy soils provide an ideal habitat for the intermediate snail hosts of *S. mansoni*. A high density of *Biomphalaria pfeifferi*, the intermediate host of *S. mansoni*, correlated with a considerable cercarial shedding rate has been previously reported in Baloua [19]. Moreover, the swampy soil and irrigated farmlands of the Baloua neighborhood, sustained by water from the Mock River, enable off-season farming, a common agricultural practice that serves as a major anthropogenic driver of hyperendemicity by increasing human–water contact.

Beyond community-level differences, sociodemographic analysis indicates that sex was not a significant factor of variation in our study population, aligning with findings from other investigations conducted in endemic African contexts [31–33] and even within the same study site among school-aged children [8]. This trend may be explained by the fact that men and women, although engaged in distinct daily tasks, ultimately share a comparable level of exposure to contaminated water sources. Women are frequently exposed during household chores such as laundry, dishwashing, and water collection, and occasionally through agricultural activities, whereas men are more frequently exposed through farming, fishing, or watering livestock. The reliance on natural water bodies thus represents both a daily necessity and a culturally embedded practice, minimizing gender-based differences and accounting for the relatively similar prevalence observed between sexes.

In contrast, age emerged as a high-risk factor for *S. mansoni* infection, with a clear declining trend in infection rates as age increased. This observation may be explained by reduced exposure over time. Young adults (18–39 years) are more actively involved in high-risk activities, either for income-generating purposes or recreational pursuits. Older individuals, on the other hand, often demonstrate more cautious behaviors, reinforced by progressive physical limitations, such as choosing safer water sources. In addition, repeated exposures to the parasite over a lifetime may lead to the development of partial acquired immunity, which can reduce parasite burden or decrease the risk of reinfection in adulthood. Similar patterns have been reported in several studies from sub-Saharan Africa, where prevalence declines after peaking in adolescence/early adulthood [34–36]. Occupational status also emerged as a key determinant of risk to *S. mansoni* infection, with farmers and breeders being particularly exposed due to their frequent and prolonged contact with contaminated water sources. As highlighted by several authors, irrigated agricultural practices substantially contribute to the persistence of schistosomiasis in rural areas of sub-Saharan Africa [28,30,36–38].

The present study also highlighted problematic alcohol consumption, with an overall prevalence of 54.3% for alcohol abuse, which combines harmful use of alcohol and alcohol dependence. This particularly high level fits within a concerning national context. According to the WHO, Cameroon ranks among the African countries with the highest per capita alcohol consumption, with rural areas appearing especially vulnerable due to abundant and poorly regulated availability [13]. In Makenene, the proliferation of drinking establishments, the wide availability of low-cost traditional drinks (palm wine, “cha,” and “odontol”), the scarcity of healthy recreational infrastructures and activities, as well as the absence of effective regulation on minimum drinking age and sales hours, all represent structural drivers of excessive alcohol consumption [39]. Illustratively, 16% of the participants in this study reported having started to drink alcoholic beverages more regularly between the ages of 10 and 15, and 40% between 16 and 20 years old. Given that the legal drinking age in Cameroon is 18 years, these data highlight the poor prevention and regulatory policies of abusive alcohol consumption. These findings are consistent with observations by Rabotata *et al*. [40] and Martín-Turrero *et al.* [41], who emphasize the major role of multiple points of sale and the financial accessibility of alcoholic beverages in driving alcohol abuse in resource-limited contexts.

Furthermore, all types of alcoholic beverages analyzed in this study were significantly associated with alcohol abuse, indicating that regular consumption, regardless of the beverage type, constitutes a high-risk behavior in this community. This finding suggests that the risk of alcohol abuse in this setting is not restricted to a specific beverage category but rather reflects broader patterns of frequent and socially normalized alcohol consumption. This study further revealed that social and conformity motives were strongly associated with alcohol abuse, confirming that drinking is often perceived less as an individual need than as a group norm [39,42,43]. However, the consumption of adulterated whisky stood out due to a particularly high risk of abuse, suggesting a strong addictive potential. This trend may be explained by the high alcohol content of these products, reaching approximately 43% for certain varieties marketed in Cameroon, as well as contextual factors such as their very low cost and wide availability, even in small retail shops. This combination enhances accessibility and exacerbates the risk of overconsumption within the community, aligning with findings by Ferreira-Borges *et al.* [14] and Staton *et al.* [44], who identified cheap local alcoholic beverages as major drivers of alcohol abuse in sub-Saharan Africa.

Disparities in alcohol consumption patterns by sex are a globally observed phenomenon, with men tending to consume larger quantities and at higher frequencies [13,42]. The findings of this study align with this trend and are consistent with WHO estimates for 2019 regarding sex-specific levels of alcohol consumption in Cameroon [13]. This pattern can be attributed to a combination of physiological, sociocultural, and psychological factors: men’s lower biological sensitivity to the harmful effects of alcohol may influence consumption behaviors [39,45]; social pressure to drink is more pronounced in public and economic spheres; and cultural norms often valorize alcohol as a marker of masculinity and conviviality [46,47]. Although women are less affected, they are not exempt from risk, particularly in rural contexts where collective celebrations (funerals, weddings, traditional festivals) foster consumption. However, the persistent stigma attached to female drinking may account for their lower apparent involvement [47–50].

The difference in the prevalence of alcohol abuse observed between communities is likely attributable to the higher density of drinking establishments and artisanal beverages outlets, as well as the greater affordability of these beverages in the Carrière and Mock-Sud neighborhoods, compared with Baloua and Mock-Centre.

Age also showed a notable influence, with middle-aged adults (40–59 years) presenting a higher likelihood of alcohol abuse with an AUDIT score ≥ 7. As observed in other African contexts [51], the higher prevalence observed in middle-aged adults suggests that a greater proportion of people in this age group exceed the threshold for hazardous drinking. This may reflect increased socioeconomic pressures, occupational stress, and social drinking practices commonly occurring during this stage of life. Indeed, data from this study suggest that occupational status is an indirect factor of exposure, with farmers being the most represented among regular drinkers. This tendency may be linked to the strenuous nature of agricultural work, which can drive some individuals to use alcohol as a means of relieving fatigue, enhancing sociability, or managing stress [39,42].

A joint analysis of the data revealed a notable comorbidity between *Schistosoma mansoni* infection and alcohol abuse, with a prevalence of 17.5%. This reflects a concerning public health situation [1]. The coexistence of these two conditions can be explained by the persistent schistosomiasis endemicity in these communities alongside high levels of alcohol consumption, particularly in its harmful forms. This dual burden further weakens already vulnerable populations, in line with the observations of Teixeira *et al*. [52], Pillai *et al*. [53], and Tharmalingam *et al.* [54] on the immunosuppressive effects of alcohol that may facilitate parasitic infections, as well as Belete *et al.* [15], Goma *et al.* [55], and Turyasiima *et al.* [56] on harmful alcohol consumption in sub-Saharan Africa.

The analysis of associated factors revealed that schistosomiasis and alcohol abuse comorbidity in Makenene did not simply result from the juxtaposition of two isolated issues, but rather reflected a synergy of spatial, demographic, and behavioral vulnerabilities. First, the place of residence emerged as the main determinant of this pathological coexistence. The neighborhoods of Baloua and Carrière displayed the highest risks of comorbidity (AORs of 4.99 and 2.37, respectively), illustrating an overlap between persistent parasitic transmission and local cultural norms that promote alcohol consumption. These inter-community disparities reflect the “persistent foci” described in several studies [57–58], where schistosomiasis remains endemic despite control efforts, and are also consistent with the findings of Sudhinaraset *et al*. [59], who highlighted the influence of social and cultural norms on alcohol consumption.

However, a comparative analysis between the communities of Baloua and Carrière provides an important nuance in understanding comorbidity in the locality of Makenene. Although these two neighborhoods presented comparable comorbidity prevalences (23.5% and 23.7%), the underlying epidemiological dynamics differed significantly. In Baloua, comorbidity appeared to be primarily driven by the hyperendemicity of schistosomiasis, with a prevalence reaching 50.4% and a significant association with infection risk (AOR = 2.64). Furthermore, the risk of alcohol abuse initially appeared lower compared with other communities in the univariate analysis (OR = 0.47). In this context, comorbidity resulted from particularly intense parasitic exposure, which could encompass, even in relatively limited proportions, individuals exhibiting abusive alcohol consumption behaviors. In contrast, in the Carrière neighborhood, the dynamics of comorbidity appeared to be more strongly influenced by behavioral factors. Although the prevalence of parasitic infection was moderate (33.6%), this community was characterized by a very high proportion of alcohol abuse, affecting nearly 70% of the adult population. In this context, the high prevalence of abusive alcohol consumption favored the co-occurrence of the two conditions, even in the presence of a lower parasitic risk than that observed in Baloua. These findings suggest that, despite a similar overall prevalence of comorbidity, public health interventions should be adapted to local realities, prioritizing strengthened transmission control measures in Baloua while reinforcing prevention strategies and interventions aimed at reducing harmful alcohol consumption in Carrière.

This spatial dynamic was reinforced by a specific demographic profile, in which young adults aged 18–39 years emerged as the most vulnerable group. This result appears consistent with the fact that this economically active age group combines increased occupational exposure to schistosomiasis transmission sites [35] with a social life conducive to alcohol consumption [14].

Furthermore, the results of this study highlighted certain behavioral practices that may increase the risk of comorbidity. Regularly performing household tasks at schistosomiasis transmission sites was significantly associated with an increased risk of *Schistosoma mansoni* infection, a finding consistent with observations reported by Tazebew et al. [28] and Ogweno et al. [29]. At the same time, regular consumption of certain beverages, particularly adulterated whisky, beer, and palm wine, was strongly associated with alcohol abuse. A remarkable aspect of this analysis concerned the role of adulterated whisky.

Despite the particularly high risk of abusive consumption associated with adulterated whisky, this type of drink was not significantly associated with the comorbidity of schistosomiasis and alcohol abuse in our study and was therefore not included in the final binary logistic regression model. This lack of association may be explained by the relatively small proportion of individuals reporting regular consumption of this type of beverage within the study population (9.8%). Thus, although this product has substantial addictive potential, the relatively limited number of regular consumers in the sample likely prevented the observation of a statistically significant overlap with individuals infected with *S. mansoni*. Conversely, due to their wide availability and their strong integration into daily consumption habits, palm wine (40.1%) and beer (37.0%) favored a greater overlap between the two conditions in this population. Therefore, the absence of an observed association between comorbidity and adulterated whisky consumption likely reflects not an absence of potential effect, but rather a limitation related to the distribution of consumption behaviors within the sample.

Motivations underlying alcohol consumption constitute an important element for indirectly interpreting the observed comorbidity. In our study, social and conformity-related aspects, such as seeking conviviality and enhanced social integration, were strongly associated with alcohol abuse, but did not emerge as significant determinants of comorbidity in the multivariate analysis. This observation suggests that these motives primarily drive excessive alcohol consumption, which may, under conditions of environmental exposure, contribute to the co-occurrence of *S. mansoni* infection. These findings, consistent with several other studies [42,43,46], corroborate the motivational models of Cooper [24] and Kuntsche *et al.* [60], which classify social and psychological motives as the main predictors of excessive consumption [44].

In addition to these identified risk factors, shared environmental and social exposures may also be considered. In some rural communities, routine water-related activities (domestic chores, bathing, fishing, etc.) occur alongside informal social gathering spaces located near transmission sites, where alcohol consumption is common. This spatial and social convergence of behaviors may simultaneously promote parasite exposure and abusive alcohol use, thereby contributing to shared risk profiles. The presence of these determinants suggests that schistosomiasis and alcohol abuse comorbidity is not merely the sum of two distinct problems but reflects shared social, cultural, and psychological vulnerabilities that amplify risks.

The coexistence of *Schistosoma mansoni* infection and alcohol abuse raises crucial questions regarding their clinical implications, particularly with respect to hepatic morbidity. While chronic *Schistosoma mansoni* infection induces peri-sinusoidal granulomatous inflammation and periportal fibrosis [9,16], alcohol overconsumption promotes hepatocellular injury, oxidative stress, and progressive fibrogenesis [10–11]. The concomitance of these two pathologies may therefore exert a synergistic effect on liver damage, potentially accelerating the progression of hepatic fibrosis and increasing the risk of severe hepatosplenic complications in endemic areas. Experimental studies have indeed reported aggravated hepatic lesions [17] and renal damage [18] in mice infected with *S. mansoni* and subjected to chronic alcohol exposure.

Furthermore, alcohol consumption may alter praziquantel bioavailability, potentially compromising treatment efficacy in comorbid individuals. Praziquantel is metabolized by several cytochrome P450 isoenzymes, including CYP3A4 and CYP1A2 [61]. The influence of alcohol on these enzymes could accelerate drug clearance, thereby reducing plasma concentrations below the therapeutic threshold [62]. Combined approaches, including chemoprevention, reduction of environmental risk factors, and the promotion of responsible drinking behaviors, are therefore essential to advance toward the elimination targets for schistosomiasis while mitigating the impact of alcohol abuse on population health [26].

Some limitations should be considered when interpreting our findings. First, the use of different diagnostic approaches for *S. mansoni* infection, POC-CCA for a minority of participants and KK for the majority, may have introduced variability in prevalence estimates. This methodological heterogeneity may have led to either an underestimation or an overestimation of *S. mansoni* infection. The use of a composite reference criterion (considering a participant infected if at least one of the two tests was positive), although commonly adopted in field studies to improve diagnostic sensitivity, remains imperfect. In particular, the POC-CCA test may occasionally yield false-positive results, which could have led to an overestimation of the prevalence of infection and consequently of the observed comorbidity. However, this approach remains widely used in the field to improve overall sensitivity by combining POC-CCA and KK. In addition, assessing alcohol consumption levels and risk factors for both conditions relied exclusively on self-reported data, which is subject to recall and social desirability bias, potentially underestimating the true extent of these factors and their contribution to the observed dynamics. Furthermore, the extrapolation of the findings from this study should be undertaken with caution, as the data are mainly representative of semi-rural areas with similar endemic and socio-economic characteristics. In addition, unmeasured factors, including socioeconomic status and access to healthcare, may limit the generalizability of these study results to different epidemiological, sociocultural, and economic contexts.

## Materials and methods

### Ethical statement

This study was approved by the Regional Committee of Research Ethics for Human Health of the Centre region, under the authority of the Ministry of Public Health of Cameroon, Cover Letter CE N° 213/CRERSHC/2022. Written informed consent was obtained from all participants prior to inclusion in the study. Authorization to conduct the study was also granted by the Ndikinimeki Health District, which oversees the Makenene health area (N° 005–21/AE/MSP/DRSPC/DSN). The field survey was carried out in the communities with the agreement of local administrative and traditional authorities. After a detailed explanation of the study objectives, procedures, and the potential risks and benefits related to the research and participants’ health, each adult residing in the target communities was free to decide whether to participate. Each participant signed an informed consent form. Data were anonymized during analysis to ensure participants’ confidentiality. The results of parasitological and immunological tests were delivered individually, and all *S. mansoni*-positive participants received, free of charge, a single dose of praziquantel (40 mg/kg body weight) in accordance with WHO recommendations [1]. Finally, all participants were sensitized to risk behaviors associated with schistosomiasis transmission and to the harmful effects of alcohol abuse.

### Study area

The study was conducted in Makenene, a semi-rural city in the Mbam and Inoubou Division, Centre Region of Cameroon. Located about 200 km northwest of Yaounde, Makenene covers an area of 885 km^2^ and has an estimated population of 16,000 inhabitants. Schistosomiasis transmission in this area is favored by a large hydrographic network that includes several rivers, notably the Mock, Makombe, Makongo, and Makenene rivers, as well as numerous streams that mainly flow into the Mock River and the Noun River, which borders the city. It also encompasses swampy areas, particularly in the Baloua neighborhood, characterized by persistent humidity throughout the year due to the proximity of the water table. The local population is primarily engaged in market gardening and off-season crop farming.

The Mock River is the principal site of schistosomiasis transmission, hosting a high density of intermediate host snails of *S. mansoni* (*Biomphalaria pfeifferi*), thereby sustaining the parasite’s life cycle [19,20]. *S. mansoni* infection has been reported in Makenene for more than three decades, with prevalence rates ranging from 82% in 1987 [21] to 41% in 2010 [7] and 40.7% in 2019 [8]. Among the sixteen villages constituting the geographical area of Makenene, three located along the Mock River were selected for this survey: Mock Centre, Mock Sud, and Carrière. Additionally, the Baloua neighborhood, although administratively situated within the village of Mock Centre, was investigated as a distinct, standalone study site due to its immediate proximity to the Mock River and its historically documented hyper-endemicity [8,19,20]. Consequently, the community-based component of this study evaluated four independent sites: Mock Centre, Mock Sud, Carrière, and Baloua.

In addition to these environmental characteristics, Makenene serves as a major commercial crossroads on National Road No. 4, linking the capital city, Yaounde, to the West Region. This geographical location fosters intense trade dynamics and a high availability of various alcoholic beverages, both industrial and artisanal. The local lifestyle, primarily centered on agricultural activities, is intertwined with alcohol consumption practices that are deeply embedded in the social and cultural context.

### Study design and population

A cross-sectional study was conducted at the community level in May 2022 in the Makenene health area. Although *Schistosoma mansoni* infection affects the entire population regardless of age or sex, this study was restricted to individuals aged 18 years and above. This specific criterion was dictated by the need to assess alcohol consumption in accordance with the legal and ethical framework of Cameroon, where the minimum legal age for alcohol consumption is 18 years.

In the absence of data on alcohol consumption in Makenene, the sample size calculation was based on the prevalence of schistosomiasis mansoni. The minimum sample size was calculated using the following formula: N = t^2^ *p *(1-p)/ m, where “N” is the sample size, “t” is the coefficient corresponding to a 95% confidence interval (1.96), “p” is the historical prevalence of intestinal schistosomiasis in Makenene estimated at 40.7% in 2019 [8], and “m” the margin of error set at 5%.

This method resulted in a minimum sample size of 371 participants, corresponding to an expected number of at least 93 individuals per community site. Participant recruitment was conducted through a door-to-door approach, starting from human-water contact points and moving toward the periphery.

### Questionnaire

A structured digital interview questionnaire was used to collect data on sociodemographic characteristics, alcohol consumption level, and behaviors associated with both schistosomiasis risk and alcohol abuse. The survey was uploaded to the Survey 123 platform and administered by field epidemiologists who were familiar with both the questionnaire and digital data collection procedures. Responses were monitored in real time, with field supervisors ensuring data completeness and accuracy.

#### Socio-environmental characteristics.

The socio-environmental variables analyzed included age, sex, educational level, primary occupation, community of residence, and the relative distance between the place of residence and the transmission site, given their potential role in exposure to both parasitic and behavioral risk factors.

#### Assessment of alcohol consumption level.

Alcohol consumption was evaluated using the Alcohol Use Disorders Identification Test (AUDIT), developed by the World Health Organization [22]. The responses to the AUDIT items are rated on a 4-point Likert scale ranging from 0 to 4, yielding a maximum possible score of 40. The quantitative score provided by this standardized instrument (AUDIT) reflects the level of alcohol consumption and allows the classification of participants into four categories: non-use (AUDIT score = 0), low-risk use (AUDIT score = 1–6), harmful use (AUDIT score = 7–12), and alcohol dependence (AUDIT score ≥ 13) [23]. Based on the potentially injurious effects of alcohol on health, a binary categorization was applied, distinguishing abusive consumption (AUDIT score ≥ 7) from non-abusive consumption (AUDIT score ≤ 6).

#### Determination of risk behaviors for *Schistosoma mansoni* infection.

Several lifestyle habits likely to facilitate the transmission of schistosomiasis were recorded, including performing household chores and fishing at the transmission site, as well as, their respective frequencies. Participants were also asked about their knowledge of the disease, previous treatment with praziquantel, and the occurrence of symptoms suggestive of infestation during contact with transmission sites (e.g., itching or skin rash upon water exposure).

#### Determination of factors associated with alcohol abuse.

Drinking habits and motivations related to alcohol consumption were examined as potential determinants of abuse. The analysis considered both the type of beverage consumed and the frequency of intake. Alcoholic beverages identified in this study included beer, wine (red or white), spirits, and adulterated whisky, which are industrial products. Among traditional beverages, palm wine and two local drinks, “cha” (a maize turbid beer) and “odontol” (made from palm wine, sugar, and a tree bark) were considered.

Alcohol consumption motives were assessed using a series of eight specific questions posed to participants. Each item aimed to identify a particular reason for drinking alcohol (e.g., seeking pleasure, coping with stress, or socializing). To facilitate structured analysis within a validated conceptual framework, these motives were then grouped according to Cooper’s four-factor model [24]. This model classifies motives into social motives (drinking to enhance or facilitate social interactions), coping motives (drinking to reduce negative emotions or deal with psychological difficulties), enhancement motives (drinking to seek positive sensations and increase pleasure), and conformity motives (drinking driven by social pressure or the need for acceptance in a group).

### Diagnosis of *Schistosoma mansoni*

#### Sample collection.

Following questionnaire administration, stool and urine samples were collected from each participant at their household. Participants were informed about the collection procedures and provided with two plastic containers to separately collect stool and urine. A unique identification code was affixed to each container and the corresponding survey form to ensure sample traceability.

**Fig 1:**
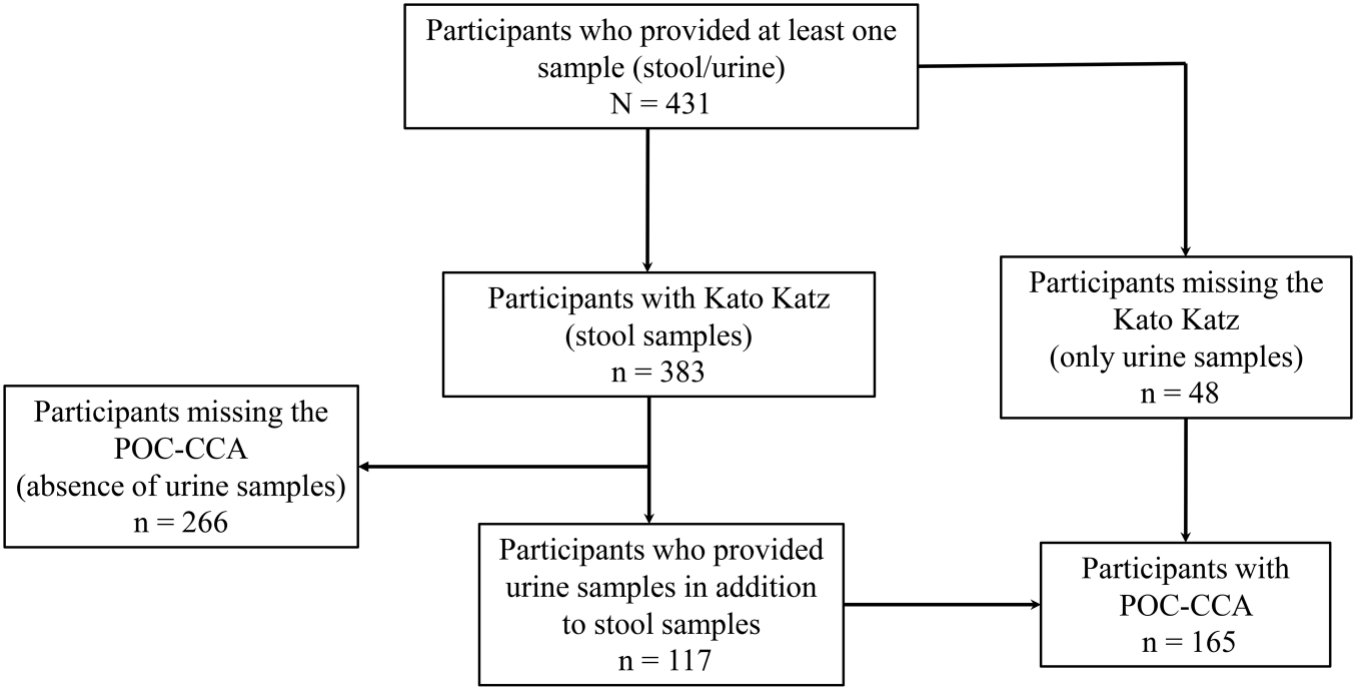
Flowchart of participant enrolment and sample collection for *Schistosoma mansoni* diagnosis.

From a total of 431 participants, 383 provided stool samples for *S. mansoni* diagnosis using the KK technique (89% of sampling size), and 165 provided urine samples for the POC-CCA test (38% of sampling size). The number of participants who underwent the POC CCA examination was not representative of the study population. Among the study participants, 117 individuals were evaluated using both the Kato–Katz technique and the POC-CCA test (Fig 1). Urine samples were analyzed directly on site, while stool samples were transported in airtight plastic bags at ambient temperature to the laboratory of the Makenene Sub-district Health Center for *S. mansoni* diagnosis using the KK method. All analyses were performed within a maximum of 24 hours after collection. A participant was considered infected with *S. mansoni* when she/he was diagnosed positive by at least one of the diagnostic methods.

#### Detection of *Schistosoma mansoni* infection using the Kato-Katz method.

Each stool sample was examined using a single thick smear prepared according to the KK technique, with a standardized template of 41.7 mg [25]. After homogenization, a portion of the stool was sieved through a 212 µm mesh screen using a spatula. The sieved material was then transferred into a single-use template, mounted on a glass slide, and covered with a rectangle of cellophane soaked in the glycerol-malachite green solution. The smear was obtained by inverting the slide onto a smooth surface and applying gentle pressure to evenly spread the sample.

The slides were examined under a light microscope within 12 h of preparation. A participant was considered positive when *S. mansoni* eggs, identifiable by their characteristic oval shape and lateral spine, were detected. Infection intensity was quantified by multiplying the total number of eggs observed per slide by a factor of 24 and expressed as a geometric mean of eggs per gram of stool (EPG). Positive samples were categorized into three groups according to infection level: light (1 – 99 EPG), moderate (100–399 EPG), and heavy (≥ 400 EPG) [1].

#### Detection of circulating cathodic antigens of *Schistosoma mansoni.*

The detection of circulating cathodic antigens (CCA) of *S. mansoni* was performed on freshly collected urine samples (within 15 min post-collection) using a point-of-care rapid diagnostic test (POC-CCA; Rapid Medical Diagnostics, Pretoria, South Africa, lot n° 211110105), administered directly at the participants’ households.

According to the manufacturer’s instructions, two drops of the urine sample were put into the circular well of the POC-CCA cassette. After complete absorption of the sample for exactly 20 min, the cassette was visually inspected. A test was considered valid if the control line turned a dark pink color. Otherwise, the sample was retested using a new cassette. A test was considered positive when, in addition to the control line, a test line was also visible.

### Statistical analysis

The collected data were exported in Excel format and then transferred to SPSS software version 27 for analysis. Qualitative variables were expressed as percentages, with confidence intervals (CI) provided for prevalence estimates. The Pearson’s χ² test was used to compare prevalence between groups. Participants were classified into three main age groups: young adulthood (18 – 39 years old), middle adulthood (40 – 59 years old), and old age (≥ 60 years old).

The normality of distributions for quantitative variables was assessed using the Shapiro–Wilk and Kolmogorov–Smirnov tests. Regarding parasite burden, results were expressed as the geometric mean associated with its 95% confidence interval. All other quantitative variables (including demographic variables and AUDIT scores) were expressed as the median and interquartile range (IQR). Mann–Whitney and Kruskal–Wallis tests were applied for comparisons involving two groups and more than two groups, respectively.

A binary logistic regression analysis was also performed using a stepwise backward elimination method to assess associations between variables. Odds ratios were estimated along with their 95% confidence intervals. Initially, a univariate analysis was conducted to examine the association between each independent variable and the outcomes of interest (schistosomiasis and alcohol abuse). Variables with a *p*-value < 0.05 and a defined OR at this stage were selected as potential risk factors for schistosomiasis and alcohol abuse comorbidity and subsequently included in the binary logistic regression model to identify factors independently associated with *S. mansoni* infection, alcohol abuse, as well as schistosomiasis and alcohol abuse comorbidity. For all analyses, the level of statistical significance was set at 0.05.

## Conclusion

This study provides, for the first time, documented evidence of comorbidity between *Schistosoma mansoni* infection and alcohol abuse in a semi-rural area of Cameroon. The findings highlight a moderate prevalence of schistosomiasis among adults, coupled with harmful alcohol use affecting more than half of the participants. The analysis of sociodemographic and behavioral factors reveals that regular consumption of palm wine and beer, along with factors associated with agricultural activities and frequent human-contaminated water sources, exacerbates this dual health burden.

The identification of this interaction underscores the need to integrate alcohol abuse prevention into schistosomiasis control strategies to enhance the effectiveness of public health interventions aimed at eliminating schistosomiasis.

## Supporting information

S1 Table Questionnaire. (DOCX)

S2 Table Distribution of the four groups for the entire cohort. S.m-/Alc-: *S. mansoni*-negative and non-alcohol-abusing participants. S.m-/Alc + : *S. mansoni*-negative and alcohol-abusing participants. S.m + /Alc-: *S. mansoni*-positive and non-alcohol-abusing participants. S.m + /Alc + : *S. mansoni*-positive and alcohol-abusing participants. n: number of participants; %: prevalence. EPG: Egg per gram of stool; AUDIT: Alcohol Use Disorder Identification Test score (represents the median score of the participants in each group); IQR: interquartile range.
(DOCX)

S1 File Schistosomiasis and alcohol use status. POC-CCA: point-of-care circulating cathodic antigen; ADS: alcohol dependence score.
(XLSX)
