# Supplementary material for: Schistosomiasis mansoni and alcohol abuse comorbidity: Prevalence and risk factors among adults in Makenene, Cameroon
Source: PLoS Negl Trop Dis. 2026 Jul 6;20(7):e0013687. doi: 10.1371/journal.pntd.0013687 (PMC13349307; doi:10.1371/journal.pntd.0013687)
Supplement: S1 Table — (DOCX) [file pntd.0013687.s001.docx]

| **Code** | **Age** | **Gender** | **Community** | **Level of study** | **Occupation** |
| --- | --- | --- | --- | --- | --- |
|  |  |  |  |  |  |

**S1 Table. Questionnaire**

**PART I. ALCOHOL CONSUMPTION**

1. Not counting small sips, at what age did you approximately start drinking alcoholic beverages more regularly?

a) 10 - 15 years
b) 16 - 20 years
c) 21 - 25 years
d) over 26 years

2. How often do you have a drink containing alcohol?
a) Never
b) Monthly or less
c) 2 to 4 times per month
d) 2 to 3 times per week
e) 4 or more times per week

3. How many drinks containing alcohol do you have on a typical day when drinking?
a) 1 or 2
b) 3 or 4
c) 5 or 6
d) 7, 8, or 9
e) 10 or more

4. How often do you have six or more drinks on one occasion?
a) Never
b) Less than monthly
c) Monthly
d) Weekly
e) Daily or almost daily

5. How often during the last year have you found that you were not able to stop drinking once you had started?
a) Never
b) Less than monthly
c) Monthly
d) Weekly
e) Daily or almost daily

6. How often during the last year have you failed to do what was normally expected from you because of drinking?
a) Never
b) Less than monthly
c) Monthly
d) Weekly
e) Daily or almost daily

7. How often during the last year have you needed a first drink in the morning to get yourself going after a heavy drinking session?
a) Never
b) Less than monthly
c) Monthly
d) Weekly
e) Daily or almost daily

8. How often during the last year have you had a feeling of guilt or remorse after drinking?
a) Never
b) Less than monthly
c) Monthly
d) Weekly
e) Daily or almost daily

9. How often during the last year have you been unable to remember what happened the night before because you had been drinking?
a) Never
b) Less than monthly
c) Monthly
d) Weekly
e) Daily or almost daily

10. Have you or someone else been injured due to your drinking?
a) No
b) Yes, but not in the last year
c) Yes, during the last year

11. Has a relative or friend or a doctor or another health worker been concerned about your drinking or suggested you cut down?
a) No
b) Yes, but not in the last year
c) Yes, during the last year

12. At which frequency do you consume the following alcoholic beverages?

|  | Never | Once a month or less | 2 times a month to 1 time a week | 2 to 3 times  a week | 4 to 5 times a week | Almost every day |
| --- | --- | --- | --- | --- | --- | --- |
| Beer |  |  |  |  |  |  |
| Wine |  |  |  |  |  |  |
| Spirit |  |  |  |  |  |  |
| Adulterated whisky |  |  |  |  |  |  |
| Palm wine |  |  |  |  |  |  |
| « Odontol » |  |  |  |  |  |  |
| « Cha » |  |  |  |  |  |  |

13. Why do you drink alcoholic beverages?

|  | Never/ almost never | Sometimes | Half the time | Often | Almost always/ always |
| --- | --- | --- | --- | --- | --- |
| It helps me to enjoy the evening |  |  |  |  |  |
| It makes social activities more fun |  |  |  |  |  |
| It helps me when I feel depressed or nervous |  |  |  |  |  |
| This goes up the moral when I'm in a bad mood |  |  |  |  |  |
| To forget my worries |  |  |  |  |  |
| To better integrate me into my attendance |  |  |  |  |  |
| To be appreciated |  |  |  |  |  |
| Because I love the feeling of drunkenness |  |  |  |  |  |
| Other (to be specified) |  |  |  |  |  |

**PART II: INTESTINAL SCHISTOSOMIASIS**

**I. Sociodemographic data and knowledge of schistosomiasis**

1. Do you know intestinal and hepatic schistosomiasis?
a) Yes
b) No

2. How far are you from the nearest transmission site?
a) Less than 0.5 km
b) Between 0.5 and 1 km
c) More than 1 km

3. Did you take praziquantel during deworming campaigns?
a) Never
b) Sometimes
c) Regularly

4. If yes, when was the last shot taken?

**II. Risky behaviors**

1. Do you do the dishes/laundry at the transmission site?
a) Daily
b) Weekly

c) Monthly
d) Never

2. Are you fishing at the transmission site?
a) Daily
b) Weekly

c) Monthly
d) Never

**III. Probable signs of infestation**

1. Do you have itching on the exposed parts after contact with water from the transmission site?
a) Never
b) Sometimes
c) Regularly

2. Do you have rashes after contact with the transmission site?
a) Never
b) Sometimes
c) Regularly
