# Supplementary material for: Schistosomiasis mansoni and alcohol abuse comorbidity: Prevalence and risk factors among adults in Makenene, Cameroon
Source: PLoS Negl Trop Dis. 2026 Jul 6;20(7):e0013687. doi: 10.1371/journal.pntd.0013687 (PMC13349307; doi:10.1371/journal.pntd.0013687)
Supplement: S2 Table — S.m-/Alc-: S. mansoni-negative and non-alcohol-abusing participants. S.m-/Alc + : S. mansoni-negative and alcohol-abusing participants. S.m + /Alc-: S. mansoni-positive and non-alcohol-abusing participants. S.m + /Alc + : S. mansoni-positive and alcohol-abusing participants. n: number of participants; %: prevalence. EPG: Egg per gram of stool; AUDIT: Alcohol Use Disorder Identification Test score (represents the median score of the participants in each group); IQR: interquartile range. (DOCX) [file pntd.0013687.s002.docx]

**S2 Table: Distribution of the four groups for the entire cohort**

| **Groups** | | **S.m-/Alc-** | **S.m-/Alc+** | **S.m+/Alc-** | **S.m+/Alc+** |
| --- | --- | --- | --- | --- | --- |
| **Prevalence** | **n (%)** | 123 (28.5) | 159 (36.9) | 74 (17.2) | 75 (17.4) |
|  | **95% CI** | 22.4 - 32.7 | 32.7 - 41.8 | 13.7 - 21.1 | 13.7 - 21.3 |
| **EPG** | **Mean (95% CI)** | 0 | 0 | 106.0 (74.4 – 150.9) | 95.4 (66.8 – 136.4) |
| **AUDIT** | **Median (IQR)** | 2.0 (1.0 – 4.0) | 12.0 (10.0 – 18.0) | 2.5 (1.0 – 4.0) | 12.0 (9.0 – 16.0) |

S.m-/Alc-: *S. mansoni*-negative and non-alcohol-abusing participants

S.m-/Alc+: *S. mansoni*-negative and alcohol-abusing participants

S.m+/Alc-: *S. mansoni*-positive and non-alcohol-abusing participants

S.m+/Alc+: *S. mansoni*-positive and alcohol-abusing participants

N: number of participants; %: prevalence

EPG: Egg per gram of stool; AUDIT: Alcohol Use Disorder Identification Test score (represents the median score of the participants in each group); IQR: interquartile range.
