# Supplementary material for: Schistosomiasis mansoni and alcohol abuse comorbidity: Prevalence and risk factors among adults in Makenene, Cameroon
Source: PLoS Negl Trop Dis. 2026 Jul 6;20(7):e0013687. doi: 10.1371/journal.pntd.0013687 (PMC13349307; doi:10.1371/journal.pntd.0013687)
Supplement: Attachment — Submitted filename: manuscript review.docx [file pntd.0013687.s004.docx]

The manuscript *“Schistosomiasis mansoni–*alcoholism comorbidity: prevalence and risk factors among adults in Makenene, Cameroon*”* by Yimgoua *et al.* provides an update on the distribution and risk factors of intestinal schistosomiasis in several communities of a health district that is a traditional focus of schistosomiasis transmission in Cameroon. The authors also present valuable information on alcohol abuse prevalence and associated risk factors in the study area. Finally, they assess co‑infection patterns across communities and analyze them according to socio‑demographic characteristics.

Overall, the manuscript is well written and provides important insights that could inform both schistosomiasis control and alcohol‑abuse prevention.

However, several clarifications are needed to improve the quality of the manuscript:

1. **Lines 31–33:** The comparison between Kato-Katz and POC-CCA, while scientifically interesting, appears outside the scope of the current paper.
2. **Lines 38–40:** For clarity, please provide odds ratios with their corresponding 95% confidence intervals.
3. **Lines 56–57:** While this point is relevant, the next logical step would be to investigate how one condition influences the other.
4. **Lines 58–59:** Please clarify how this finding could inform the development of targeted interventions.
5. **Lines 172–174:** Remove the commas before and after “as well as.”
6. **Lines 198–199:** Specify the exact transportation conditions. Were samples stored in a cooler with ice, or kept at ambient temperature?
7. **Lines 201–205:** Clarify the rationale for using both Kato-Katz and POC-CCA. Since Kato-Katz is considered the gold standard, why was POC-CCA also included? If there is a specific justification, please state it clearly.
8. **Lines 239–244:** Although interesting, this section is not central to the study’s objectives and may distract the reader. Numerous studies already compare POC-CCA and Kato-Katz. Unless this comparison adds novel value, I suggest removing it or presenting it separately as a short report.
9. **Lines 260–261:** Was a normality test performed to assess whether the data were normally distributed? If not, demographic variables should be presented using median and interquartile range.
10. **Table 1:** Please clarify what is included in the category “Other.”
11. **Table 2:** This table appears unnecessary, as all information is already described in the text.
12. **Table 3:** Consider presenting these data using a scatter plot for better visualization.
13. **Lines 288–313:** As previously mentioned, this section does not contribute to the study’s main objectives and could be removed.
14. **Table 5:** A graphical representation may be more effective (e.g., a histogram for prevalence and a scatter plot for infection intensity).
15. **General comment:** Were normality tests applied to all variables? Many variables are presented as mean ± SD, which assumes normal distribution.
16. **Lines 409–415:** This section does not provide new information, as community comparisons were already presented earlier. A more informative analysis would compare *S. mansoni* infection intensity between alcohol abusers and non‑abusers, or compare AUDIT scores between infected and uninfected participants.
17. **Lines 420–424:** This appears similar to previous comparisons. See comment 16.
18. **Lines 430–434:** See comment 16.
19. **Lines 438–443 (and all risk‑factor sections):** Consider replacing chi‑square values with odds ratios and 95% CIs, which provide more meaningful information.
20. **Lines 492–496:** Can the authors propose explanations for this finding? For example, differences in urbanization, alcohol availability, or affordability across communities?
21. **Table 12:** It is unclear which community was used as the reference category in the regression model. Since all communities show significant associations with alcohol abuse, clarification of the reference group is essential for proper interpretation of the odds ratios.
22. **Lines 606–607:** Please use more up‑to‑date references.
23. **Discussion:** The manuscript would benefit from a paragraph discussing how this comorbidity may influence pathogenesis (schistosomiasis or liver function), and how excessive alcohol consumption may affect schistosomiasis MDA outcomes (adverse events, drug efficacy). This would strengthen the public‑health relevance of the findings.
24. **Lines 728–729:** Consider adding “or overestimation.”
25. **Conclusion:** It is unclear how controlling alcohol consumption would advance schistosomiasis control, especially since the manuscript does not demonstrate a direct relationship between alcohol use and schistosomiasis risk. I recommend revising this section for clarity.
