# Supplementary material for: Schistosomiasis mansoni and alcohol abuse comorbidity: Prevalence and risk factors among adults in Makenene, Cameroon
Source: PLoS Negl Trop Dis. 2026 Jul 6;20(7):e0013687. doi: 10.1371/journal.pntd.0013687 (PMC13349307; doi:10.1371/journal.pntd.0013687)
Supplement: Attachment — Submitted filename: Response to reviewers.docx [file pntd.0013687.s005.docx]

**ANSWERS TO REVIEWERS’ COMMENTS**

**Manuscript PNTD-D-26-00185**

**Title: Schistosomiasis mansoni and alcohol abuse comorbidity: prevalence and risk factors among adults in Makenene, Cameroon**

**I- Methods**
-Are the objectives of the study clearly articulated with a clear testable hypothesis stated?
-Is the study design appropriate to address the stated objectives?
-Is the population clearly described and appropriate for the hypothesis being tested?
-Is the sample size sufficient to ensure adequate power to address the hypothesis being tested?
-Were correct statistical analysis used to support conclusions?
-Are there concerns about ethical or regulatory requirements being met?

**Reviewer #1**:

The manuscript investigates the prevalence and risk of Schistosomiasis mansoni infection and alcoholism among adults in Makenene in Cameroon. The objectives of the study are clearly stated, together with the justification of the study. As a cross-sectional study, the method is justified with adequate sample size selection and power to enable the correct statistical analysis of the. The study design and methods applied in estimating the prevalence of schistosomiasis and alcoholism are standard and acceptable.

Thank you for your appreciation

**Reviewer #2**:

The following are the points of concern to be addressed in method section:

1. Sample Size Calculation (Lines 143-148): The calculation uses a prevalence (p=41%) from 2010. Please justify why this older estimate was used and discuss whether this might affect the precision of your current estimates. Indicate whether a design effect was considered in the community-based sampling.

Tchuem Tchuente et al. (2012) and Mawamba et al. (2021) reported practically the same prevalence in the locality of Makenene (41% and 40.7%), resulting in the same sample size: 371 participants. However, in order to update estimates, we incorporated Mawamba et al. (2021) into the revised manuscript **(lines 150 and 173: reference 8)**.

2. Diagnostic Approach (Lines 201-205, Fig 1): The algorithm for defining an overall infection status (positive by KK or POC-CCA) is acceptable given POC-CCA's higher sensitivity. However, this leads to a situation where the "overall" prevalence (34.6%) is lower than the POC-CCA prevalence in its sub-sample (66.1%). This needs careful explanation in the results (Lines 267-273) to avoid confusion. Clearly state that the POC-CCA sub-sample (n=165) is not representative of the entire population and has a different demographic profile, hence the different prevalence.

This clarification has been added to the **Materials and Methods** section, where sample sizes and percentages of each subsample according to the diagnostic method relative to the total population were specified (89% for the Kato–Katz method and 38% for the POC-CCA method) **(lines 231 - 234).**	Moreover, we commented on these prevalences for better clarity in the **Results** section **(lines 304 - 312)**. Similarly, in the **Discussion** section, we emphasized that the disparity in prevalence observed between the overall population (34.6%) and the POC-CCA subsample (66.1%) may be related to distinct demographic profiles, a lack of representativeness of the latter compared with the entire cohort, or to the higher sensitivity of the POC-CCA method compared with the Kato–Katz method **(lines 630 - 636).**

3. Statistical Analysis: The use of a binary logistic regression model is appropriate. For Table 11 and Table 16, please indicate which category served as the reference for each variable in the multivariate model (e.g., Age: ≥60 years as ref; Community: perhaps Mock Sud or the largest group?).

The reference categories for each variable have been specified in both the univariate analyses **(Tables 9 and 12)** and multivariate analyses **(Tables 11, 16, and 17).**

**Reviewer #3:**

1. Lines 31–33: The comparison between Kato-Katz and POC-CCA, although scientifically relevant, appears outside the scope of the current manuscript.

Although the comparison between the Kato–Katz and POC-CCA diagnostic methods is scientifically relevant, we acknowledge that it falls outside the main scope of the present manuscript. Therefore, in order to maintain better consistency with the study objectives, this section has been removed from the revised version.

2. Lines 198–199: Please specify the exact transportation conditions for the samples. Were they stored in a cooler with ice or kept at ambient temperature?

Stool samples were transported in airtight plastic bags at ambient temperature to the laboratory of the Makenene Sub-district Health Center for analysis using the Kato-Katz method. This information was added to the **Material and Methods** section **(lines 235 - 238).**

3. Lines 201–205: Clarify the rationale for using both Kato-Katz and POC-CCA. Since Kato-Katz is considered the gold standard, why was POC-CCA also included? If there is a specific justification, please state it clearly.

The sensitivity of the Kato-Katz method decreases in cases of low-intensity infections or in settings of moderate to low transmission. The inclusion of the POC-CCA aimed to capture light *S. mansoni* infection that may be missed by the Kato-Katz method, especially in an adult population.

4. Lines 239–244: This comparison between diagnostic methods is not central to the study’s objectives and may distract the reader. Unless it adds novel value, consider removing it or presenting it separately.

Comparison between diagnostic methods has been removed from the revised manuscript.

5. Lines 260–261: Was a normality test performed to assess whether the data were normally distributed? If not, demographic variables should be presented using median and interquartile range.
6. General: Were normality tests applied to all variables? Many variables are presented as mean ± SD, which assumes normal distribution.

Normality tests (Shapiro–Wilk and Kolmogorov–Smirnov) were systematically applied to all quantitative variables, and they were not normally distributed. Consequently, we revised the statistical analyses: parasite burdens were expressed using the geometric mean (95% CI), and other quantitative variables (demographic variables and AUDIT scores) were presented as median (IQR) **(lines 272 - 277).**

**Reviewer #4:**

1- The objective of the study is not clear enough. My suggestion is “determine the prevalence of schistosomiasis-alcoholism or alcohol abuse among adults in intestinal schistosomiasis endemic area of Makenene in Cameroon as well as the risk factors associated with this comorbidity;

We have taken your suggestion into account in the new formulation of the objective within the revised manuscript. The objective has thus been reformulated as follows: " to determine the prevalence of schistosomiasis and alcohol abuse comorbidity among adults in the intestinal schistosomiasis endemic area of Makenene in Cameroon, as well as the risk factors associated with this comorbidity" **(lines 117 - 119).**

2- The section on the study design is not well presented and must be rewritten as well as the description of the study site. This description targeted schistosomiasis without any information linked to alcohol consumption in this endemic area;

The study protocol section has been reorganized to better distinguish the study type and design, the target population, and the data collection procedures **(lines 165 - 178)**. Furthermore, the description of the study site has been enriched to include, in addition to the epidemiological characteristics related to schistosomiasis, relevant contextual elements regarding alcohol consumption **(lines 156 - 161).** It is, however, important to mention that information linked to alcohol consumption in Makenene is not documented.

3- For the calculation of the sample size, the schistosomiasis prevalence was used. However, the authors have forgotten that they are dealing with schistosomiasis and alcoholism. Moreover, the disease prevalence used was too old.

The sample size calculation was based solely on schistosomiasis due to the absence of data on alcoholism in Makenene. This clarification was added to the revised manuscript **(lines 169 – 174)**.	The sample size calculation is correct as the prevalence remained practically the same over the years: 41% in 2010 (Tchuem Tchuenté et al., 2012) and 40.7% in 2019 (Mewamba et al., 2021). We have, however, updated the information, using the most recent prevalence of schistosomiasis from Mewamba et al. (2021) **(line 173).**

4- In the MM, the sections treating risk behaviors to schistosomiasis and factors associated with alcohol abuse must be part of the questionnaire

We confirm that the sections addressing schistosomiasis risk behaviors and factors associated with alcohol abuse are indeed included in the questionnaire. The questionnaire has been uploaded as Supplementary Material **(S1 Table)**.

**II- Results**
-Does the analysis presented match the analysis plan?
-Are the results clearly and completely presented?
-Are the figures (Tables, Images) of sufficient quality for clarity?

**Reviewer #1:**

The results, as presented, and the analysis of results conducted match the plan and are quite easy to follow. I am, however, a bit constrained in my understanding of some of the statistical analyses conducted. The tables are, clear, though some of the tables are loaded with too much information and could be simplified.

Thank you for your overall appreciation of the results section.

Although the tables contain a lot of information, the comprehensiveness of the data presented is important for this epidemiological study to allow for a complete interpretation of the observed interactions.

**Reviewer #2:**

In the result section of this manuscript, the following should be addressed accordingly:

1. Table 2 & Corresponding Text: As above, the discrepancy between the high POC-CCA sub-sample prevalence and the lower combined prevalence requires a clear explanatory sentence.

Clarifications explaining this discrepancy in prevalence have been added to the revised manuscript to remove any ambiguity regarding Table 2 (**Material and methods**: **lines 231– 234** and **Discussion**: **lines 630 – 636**).

2. Table 4 (Diagnostic Concordance): The calculations and presentation are good. It would be helpful to briefly state in the text that the 100% specificity for KK is due to the absence of KK+/POC-CCA- cases.

We thank you for this comment. However, to adhere to the study’s primary objective, which focuses on the prevalence of comorbidity and associated risk factors, the comparative analysis of schistosomiasis diagnostic methods has been removed from the revised manuscript.

3. Table 7 & Text on Alcohol Abuse by Age: The text (Lines 387-390) states prevalence was higher in 40-59-year old (p=0.032), but the AUDIT score did not differ (p=0.115). This is an interesting nuance. Ensure the discussion comments on why intensity (score) might be similar across ages, but the prevalence of "abuse" (AUDIT≥7) peaks in middle age.

We thank you for this analysis highlighting the nuance between the prevalence of alcohol abuse and the intensity of alcohol intake (AUDIT score).

After revising the statistical analyses as suggested by other reviewers, the AUDIT score, expressed as the median (IQR), was 9.0 (4.0 – 13.0) for 40 to 59-year-olds, compared to 6 for other age groups (p = 0.056) **(Table 6)**.

Comments on why the alcohol consumption score might be similar across ages, but the prevalence of "abuse" peaks in middle age are included in the **Discussion** section **(lines 738 – 743).**

4. Comorbidity (Section 3.4): The analysis is precise. Consider adding a simple cross-tabulation in the supplement showing the distribution of the four groups (S.m+/Alc+, S.m+/Alc-, S.m-/Alc+, S.m-/Alc-) for the whole cohort.

A simple cross-tabulation showing the distribution of the four groups (S.m+/Alc+, S.m+/Alc-, S.m-/Alc+, S.m-/Alc-) for the entire cohort has been added to the supplementary material **(S2 Table).**

**Reviewer #3:**

1. Lines 38–40: Provide odds ratios with their 95% confidence intervals for clarity.

Odds ratios (ORs) along with their 95% confidence intervals (95% CIs) have been added to all relevant results.

2. Lines 56–57: While relevant, the next step should be to investigate how one condition influences the other.

3. Lines 58–59: Clarify how this finding could inform targeted interventions.

We thank you for these observations. We have updated the abstract to emphasize that exploring the reciprocal influence between schistosomiasis and alcohol abuse constitutes a key step for future research perspectives. Furthermore, we have specified the nature of the targeted interventions by proposing the integration of screening and awareness-raising within existing mass treatment campaigns **(lines 53 - 58).**

4. Lines 172–174: Remove the commas before and after “as well as.”

Thank you for this correction. The commas before and after 'as well as' have been removed.

5. Table 1: Clarify what is included in the category “Other.”

A footnote has been added to Table 1 to specify the occupations included in the 'Other' category **(lines 299 - 300)**.

6. Table 2: This table appears unnecessary, as all information is already described in the text.

We acknowledge the reviewer's comment about the redundancy between the text and Table 2. However, this table provides a synthetic visualization and a direct comparison of the data, which would be harder to understand within a dense paragraph. The table serves as a quick reference for the reader, complementing the textual description.

7. Table 3: Consider using a scatter plot for better visualization.

Using a scatter plot allows better visualization, but does not show results from statistical analysis. Table 3 reports the comparison of infection intensity levels using a synthetic indicator that enables direct and comparative interpretation.

8. Lines 288–313: This section does not contribute to the study’s main objectives and could be removed.

This section has been removed from the revised manuscript.

9. Table 5: A graphical representation may be more effective (histogram for prevalence, scatter plot for intensity).

Presenting prevalence and parasite burden together with their 95% confidence intervals on the same row in the table facilitates comparisons across categories of each sociodemographic variable and improves the readability of the intensity associated with each prevalence.

10. Lines 409–415: This section does not provide new information. A more informative analysis would compare *S. mansoni* infection intensity between alcohol abusers and non-abusers, or compare AUDIT scores between infected and uninfected participants.

This section, which presents the intensity of alcohol consumption among participants with schistosomiasis mansoni and alcohol abuse comorbidity according to sociodemographic characteristics, is consistent with the objective of the study.

However, in the revised manuscript, we added a table and a comment on the comparison of *S. mansoni* infection intensity between alcohol abusers and non-abusers, and a comparison of the AUDIT scores between infected and uninfected participants **(lines 436 – 441; Table 8).**

11. Lines 420–424: Similar to previous comparisons; see comment 16.
12. Lines 430–434: See comment 16

Comment 16 is about improving the quality of Figure 1. It has been improved.

13. Lines 438–443: Consider replacing chi square values with odds ratios and 95% CIs for more meaningful interpretation.

Thank you for this suggestion; it has been implemented in the revised manuscript.

14. Lines 492–496: Provide possible explanations for this finding. For example, differences in urbanization, alcohol availability, or affordability across communities.

We have discussed the disparities observed between communities in the **Discussion** section by the proliferation of drinking outlets and the lower cost of artisanal beverages in the Carrière and Mock-Sud neighborhoods than in Baloua and Mock-Centre **(lines 734 - 737).**

15. Table 12: It is unclear which community was used as the reference category in the regression model. Since all communities show significant associations with alcohol abuse, clarification is essential for proper interpretation.

The reference categories for each variable, including the community of residence, have been specified in the footnotes of Table 12 **(lines 500 – 502).**

16. Improve Figure 1’s quality

The quality of Figure 1 has been improved.

**Reviewer #4:**

The focus of the study was the comorbidity between schistosomiasis and alcoholism. However, the section treating this comorbidity is not enough. The comparison between KK and POC-CCA is not important because several studies have already compared these two tests, even in the same endemic area (see Mewamba et al. 2022). Moreover, the sample size does enable to have reliable results on the performance (sensitivity, specificity, and accuracy) of each diagnostic test. The goal standard test was not provided. Risk factors associated with schistosomiasis and alcoholism are well known; but risk factors associated with comorbidity are of great interest. The result section must be rewritten, synthesized and focused on the main objective that treats the comorbidity.

Thank you for your comments and suggestions.

In accordance with your suggestions, the section dedicated to comorbidity has been enriched with an analysis of the interactions between parasite load and AUDIT scores **(lines 436 – 441; Table 8)**. Moreover, analysis of the risk factors associated with the comorbidity has been added **(Abstract: lines 33 – 39; Results: lines 594 – 610; Table 17)**. Finally, the comparison between Kato-Katz and POC-CCA tests has been removed from the revised manuscript.

**III- Conclusions**
-Are the conclusions supported by the data presented?
-Are the limitations of analysis clearly described?
-Do the authors discuss how these data can be helpful to advance our understanding of the topic under study?
-Is public health relevance addressed?

**Reviewer #1:**

The conclusions directly support the data presented, and limitations are clearly noted in the discussions. The conclusions and recommendations raise questions for further research.

Thank you for your appreciation.

**Reviewer #2:**

The conclusion section indicates that the authors identify two key limitations: (1) diagnostic heterogeneity (use of two tests) and (2) reliance on self-reported data subject to bias. This is good, but it should be expanded.
1. The diagnostic limitation should explicitly state that the composite "gold standard" (either test positive) is imperfect, as POC-CCA may yield false positives, potentially inflating the "true" comorbidity rate.

We thank the reviewer for this comment. The limitation you pointed out has been clarified in the revised version **(lines 846 - 851).**

2. A major limitation not discussed is the cross-sectional design, which precludes determining causality or the temporal relationship between risk factors and outcomes. It cannot establish if alcohol abuse increases the risk of infection or vice versa.

Our analyses of the interactions between parasite load and AUDIT scores (Table 8), together with the assessment of risk factors (Table 10), indicate that heavy alcohol consumption is not a risk factor for *S. mansoni* infection, and that the infection doesn’t predispose individuals to alcohol abuse. Given that these two pathologies have different etiologies, a direct causal relationship in either direction seems unlikely. Nevertheless, from a physiopathological perspective, because both alcoholism and *S. mansoni* infection affect the liver, a deleterious synergy may, over time, exacerbate the hepatic damage associated with either condition. Their potential contribution to liver injury has been discussed in the **Discussion** section **(lines 825 – 833)**.

3. The potential for residual confounding caused by unmeasured variables (e.g., detailed socioeconomic status, access to healthcare) should be recognized.

We acknowledge the potential for residual confounding caused by unmeasured variables. We pointed it out in the Discussion section of the revised manuscript **(lines 856 – 859).**

4. The generalizability of findings beyond similar semi-rural, endemic foci could be briefly mentioned.

We have specified in the revised version (Discussion section) that the extrapolation of our conclusions to other geographical, socio-cultural, and economic contexts should be made with caution **(lines 854 - 859)**.

**Reviewer #3:**

1. Lines 728–729: Consider adding “or overestimation.”

We thank the reviewer for this suggestion. We have reformulated the relevant passage to account for a possible overestimation of the prevalence **(lines 844 - 846)**.

2. Conclusion section: It is unclear how controlling alcohol consumption would advance schistosomiasis control, especially since the manuscript does not demonstrate a direct relationship between alcohol use and schistosomiasis risk. This section should be revised for clarity.

Although alcohol abuse is not a risk factor for *S. mansoni* infection, or vice versa, the prevalence of their co-occurrence (17.4%) highlights the potential for a deleterious synergy in terms of liver damage. In addition, alcohol may alter drug bioavailability, potentially affecting the efficacy of praziquantel. Therefore, reducing harmful alcohol consumption in endemic areas could help limit the severity of liver pathology and optimize therapeutic response among comorbid individuals. These clarifications have been incorporated into the discussion section **(lines 834 - 841).**

**Reviewer #4:**

- The discussion must be rewritten. It must be related to the road map objective of elimination schistosomiasis as public health. The authors must discuss the high prevalence of schistosomiasis in adults in relationship with the current control strategies.

We thank the reviewer for this recommendation. The discussion has been revised to better align with the WHO NTD Roadmap (2021–2030). Our findings highlight the need for greater inclusion of adult populations in mass drug administration programs, as their relatively high prevalence of infection may contribute substantially to the persistence of the parasite reservoir. Furthermore, we clarified how considering alcohol abuse-related comorbidity could help strengthen the effectiveness of these public health interventions **(lines 641 – 647; 838 – 841)**.

- How data on comorbidity could help to improve disease control in neighborhoods. The relationship between comorbidity and liver disease was not discussed.

The potential impact of a deleterious synergy between alcoholism and S. mansoni infection on hepatic pathogenesis has been incorporated into the discussion. We also highlight that harmful alcohol consumption may alter the pharmacokinetics of praziquantel, potentially compromising its therapeutic efficacy. Consequently, integrating strategies aimed at reducing harmful alcohol use in endemic areas could not only limit the worsening of liver damage but also optimize the therapeutic effectiveness of praziquantel in patients with this comorbidity **(lines 825 – 841).**

**IV- Editorial and Data Presentation Modifications?**

Use this section for editorial suggestions as well as relatively minor modifications of existing data that would enhance clarity. If the only modifications needed are minor and/or editorial, you may wish to recommend “Minor Revision” or “Accept”.

**Reviewer #1:**

The paper is well-written in standard English and would require no modifications in my opinion.

Thank you for your appreciation.

**Reviewer #2:**

Recommendation: Minor Revision
The following suggestions aim to improve clarity, consistency, and conformity to journal formatting standards without changing the study's core methodology or findings.
1. Formatting & Journal Compliance
• Manuscript File: Prepare the main text as an editable document (e.g., .doc) with continuous line numbering, not as a PDF from the submission system.
• Headings: Ensure consistent formatting of headings (e.g., bold for main sections like Introduction and Methods).
• Financial Disclosure: Incorporate the full, correctly formatted funding statement into the main manuscript under a "Funding" section, including grant numbers and funder URLs.
• Data Availability: Replace "All relevant data are within the manuscript" with a statement compliant with PLOS policy. Example: "The data underlying the results presented in this study are available from [Repository Name, e.g., Dryad] at [DOI or URL]."

Thank you for pointing it out. The data underlying the results presented in this study are contained within the manuscript and its supporting information files.

2. Terminology & Consistency
• Location Spelling: Standardize to one spelling for the study site throughout the manuscript (check abstract, title, and text). The PDF uses both "Makehene" and "Makenene".

Thank you, the correction has been made.

• Diagnostic Tests: Consistently format test names: "Kato-Katz (KK)" and "point-of-care circulating cathodic antigen (POC-CCA)" on first use, then "KK" and "POC-CCA."

The nomenclature of diagnostic tests has been reformatted and harmonized throughout the revised manuscript.

• Statistical Notation: Use proper superscripts (e.g., t² instead of t[2] in the sample size formula). Ensure consistent use of italics for Schistosoma mansoni.

Corrections have been made.

3. Table & Figure Clarity
• Table 1: The table is clear. Ensure the final version avoids awkward page breaks.
• Table 2 & Text: Add a footnote to Table 2 or a sentence in the text explaining: "The overall prevalence was calculated for all participants (N=431), considering a participant positive if either diagnostic test was positive. The POC-CCA sub-sample (n=165) is not representative of the full cohort."

For better clarity, the suggested sentence has been added to the text related to Table 2 **(lines 309 – 312; 630 – 636)**

• Table 4 (Diagnostic Concordance): The table is excellent. Consider adding a brief parenthetical interpretation of the kappa value in the caption (e.g., "κ = 0.458, indicating moderate agreement").

The section related to the performance of diagnostic methods has been removed from the revised manuscript.

• Figure 1: Include a clear yet brief legend within the main manuscript file (e.g., "Flowchart of participant enrolment and sample collection for Schistosoma mansoni diagnosis").

The title of Figure 1 has been modified according to your suggestion.

4. Language & Flow Enhancements
• Abstract, Line 23: Rephrase for clarity: "Considering that both schistosomiasis mansoni and alcoholism can induce liver pathology..."

The sentence has been rewritten according to your recommendation.

• Methods, Sample Size (Lines 143-148): Improve sentence flow: "The minimum sample size was calculated using the formula N = t² * p * (1-p) / m², where *t* is the coefficient for a 95% confidence interval (1.96), *p* is the historical prevalence of intestinal schistosomiasis in Makehene (41%, from 2010), and *m* is the margin of error (5%). This yielded a minimum of 372 participants."

Thank you, the section has been rewritten **(lines 169 – 174).**

• Results, Section 3.1 (Lines 267-273): Add a transitional sentence: "Prevalence estimates varied by diagnostic method and sample. Among those providing urine... In contrast, the KK method applied to a larger stool sample group yielded... To generate an overall estimate for the entire cohort (N=431), a participant was considered infected if either test was positive, resulting in a prevalence of 34.6%."

Thank you, the section has been rewritten according to your recommendations **(lines 304 – 312)**.

• Discussion: Break up some long paragraphs (e.g., the paragraph spanning lines 620-670) into 2-3 focused paragraphs for easier reading.

Paragraphs have been broken up. In the revised manuscript, we ensured that the paragraphs remain concise and well-structured for optimal readability.

5. Minor Corrections

• Author Summary, Line 49: Correct spelling to match chosen standard ("Makehene" or "Makenene").

The correction has been made.

• References: Perform a final check to ensure all in-text citations have a corresponding reference and that journal name abbreviations are consistent with PLOS style.

Thank you, we performed the check-up.

Finally, Implementing these modifications will significantly improve the manuscript's presentation, professionalism, and readability, ensuring it aligns with journal standards and effectively communicates its important findings.

**Reviewer #3:** Minor Revision

**Reviewer #4:** (No Response)

**V- Summary and General Comments**
Use this section to provide overall comments, discuss strengths/weaknesses of the study, novelty, significance, general execution and scholarship. You may also include additional comments for the author, including concerns about dual publication, research ethics, or publication ethics. If requesting major revision, please articulate the new experiments that are needed.

**Reviewer #1:**

The topic of this manuscript is novel. I initially found it challenging to relate schistosomiasis infection and alcoholism but was fully convinced of the convergence after reading the background and study justification. I have no ethical concerns with the manuscript and readily recommend it for publication.

**Reviewer #2:**

Summary and General Comments
Overall Assessment:
This manuscript presents a novel, important, and well-executed study that addresses a significant gap in public health research at the intersection of infectious and non-communicable diseases. The investigation of comorbidity between *Schistosoma mansoni* infection and alcohol use disorders in a semi-rural Cameroonian setting is both timely and relevant. The study is suitable for publication in PLOS Neglected Tropical Diseases following major revisions that primarily concern methodological clarification, enhanced interpretation, and strict adherence to the journal's formatting and data policies.
Strengths:
• High Novelty & Significance: This is the first documented study of this specific comorbidity in Cameroon (and likely in similar endemic settings). It moves beyond siloed disease perspectives, highlighting a critical syndemic that exacerbates liver disease burden and challenges existing control programs.
• Strong Methodology: The community-based, cross-sectional design is appropriate. The use of standardized diagnostic tools (KK, POC-CCA, AUDIT) is commendable. The sample size is adequate, and the statistical analyses (chi-square, logistic regression, and kappa statistics) are generally applied correctly.
• Clear Public Health Relevance: The findings have direct implications for integrated disease control strategies. The identification of shared socio-ecological risk factors and distinct behavioral drivers provides a concrete evidence base for designing multifaceted interventions.
• Rigorous Ethical Conduct: The study details robust ethical approvals, informed consent processes, provision of treatment, and post-study health education, meeting high ethical standards.
Weaknesses & Required Revisions:
The weaknesses are not fatal to the study's validity but require careful revision to ensure scientific rigor, clarity, and compliance.
1. Hypothesis & Objectives: While the objectives are clear, the introduction should be refined to include an explicit, testable primary hypothesis (e.g., concerning the expected prevalence of comorbidity and its key drivers).

We thank the reviewer for this suggestion. An explicit primary hypothesis has been inserted in the introduction. We hypothesize that, given the high level of alcohol consumption in Cameroon, there is a significant prevalence of co-occurrence with *S. mansoni* infection, driven by local sociodemographic factors and risk behaviors. **(lines 94 - 97).**

2. Methodological Transparency:
a. Sample Size: Justify the sample size calculation more robustly, acknowledging its use for a comorbidity analysis and multivariate modeling.

Due to the lack of prior statistical data on alcohol consumption prevalence in Makenene, the sample size calculation was based on the prevalence of schistosomiasis. The study population (431) was superior to the minimum threshold of 371 participants; in order to improve the performance of statistical analyses regarding comorbidity and to ensure the reliability of the multivariate modeling.

b. Diagnostic Algorithm: Provide a much more straightforward explanation in the Results section for the discrepancy between the POC-CCA sub-sample prevalence and the final composite prevalence. Acknowledge the inherent limitations of the composite reference standard.

Thank you, it has been done.

c. Statistical Reporting: Clearly state the reference categories for all variables in the multivariate logistic regression models (Tables 11, 16).

Thank you, it has been done.

3. Discussion & Limitations:
a. Expand the Limitations section to explicitly discuss the constraints of the cross-sectional design (no causality), the potential for residual confounding, and the implications of the diagnostic approach.
b. Strengthen the discussion by briefly speculating on the potential synergistic clinical impact (e.g., on liver fibrosis progression) of the comorbidity.

c. Make the call for "integrated interventions" more tangible by suggesting 1-2 concrete, feasible examples (e.g., integrating AUDIT screening into schistosomiasis community outreach).

All these remarks and suggestions have been considered, and corrections have been made in the revised manuscript.

4. Journal Compliance (Critical): a. Data Availability: The current statement is unacceptable. Data must be deposited in a public repository (e.g., Dryad, Figshare) with a DOI, and the statement must be updated to reflect this. This is a mandatory requirement for publication in PLOS journals.

The data underlying the results presented in this study are contained within the manuscript and its supporting information files.

b. Financial Disclosure: The funding statement must be completed in full within the manuscript text, following the journal's prescribed format.

The funding information has been included in the manuscript. However, no award number was assigned for the grant provided by the RSTMH Small Grant 2021 to Emmanuelle S. Yimgoua (ESY).

c. Formatting: The manuscript must be reformatted to meet PLOS NTD style: include continuous line numbers, correct heading styles, and ensure figures are submitted separately with legends in the main text. General Execution and Scholarship: The scholarship is sound. The literature review is comprehensive, the data presentation is logical, and the writing is generally clear. The authors demonstrate a strong command of the subject matter. The required revisions are largely editorial and explanatory, aimed at elevating the manuscript's precision, transparency, and impact.

Ethics: No concerns regarding dual publication, research ethics, or publication ethics were identified. The ethical oversight and procedures described are thorough and appropriate. Conclusion:
This study makes a valuable and original contribution to the field. The requested revisions are essential to solidify the methodological narrative, provide a more critical self-assessment, and fulfill non-negotiable publishing criteria. I recommend "Major Revision" with the expectation that the authors can address these points through textual modifications, clarifications, and compliance updates without the need for new experiments or data re-analysis.

**Reviewer #3:**

The manuscript “Schistosomiasis mansoni–alcoholism comorbidity: prevalence and risk factors among adults in Makenene, Cameroon” by Yimgoua et al. provides an update on the distribution and risk factors of intestinal schistosomiasis in several communities of a health district that is a traditional focus of schistosomiasis transmission in Cameroon. The authors also present valuable information on alcohol abuse prevalence and associated risk factors in the study area. Finally, they assess co infection patterns across communities and analyze them according to socio demographic characteristics.
the discussion would benefit from a paragraph on how this comorbidity may influence pathogenesis (schistosomiasis or liver function) and how excessive alcohol consumption may affect MDA outcomes (adverse events, drug efficacy). This would strengthen the public health relevance of the findings

All these remarks and suggestions have been considered, and corrections have been made in the revised manuscript.

**Reviewer #4:**

Comments
This manuscript PNTD-D-25-01931 of Yimgoua et al entitled "Schistosomiasis mansoni-alcoholism comorbidity: prevalence and risk factors among adults in Makenene, Cameroon" treat an interesting topic related to intestinal schistosomiasis and alcoholism as well as their comorbidity in schistosomiasis endemic area of Makenene in Cameroon.

Major comment
The manuscript addressed two public health problems notably one of the most important neglected tropical disease and alcoholism in adults living intestinal schistosomiasis endemic area of Cameroon. Although the comorbidity between the two public health problem will be of great concern especially for their potential of inducing liver diseases, this study has a lot of short-comes.

1- The introduction is not well written. It does not provide the state of the problem, previous studies on this comorbidity, the state of alcoholism in other schistosomiasis endemic areas, data on liver diseases related to schistosomiasis or alcoholism, the knowledge gaps related to this comorbidity;

The introduction of the revised manuscript has been reformulated to address each of these concerns. It clearly presents the state of the problem, previous studies on this comorbidity, and provides data on alcohol consumption in other schistosomiasis-endemic areas. Furthermore, the manuscript provides specific details regarding liver diseases associated with both conditions. Finally, we have clearly outlined the existing knowledge gaps, thereby emphasizing the relevance of the present study.

2- The objective of the study is not clear enough. My suggestion is “determine the prevalence of schistosomiasis-alcoholism or alcohol abuse among adults in intestinal schistosomiasis endemic area of Makenene in Cameroon as well as the risk factors associated with this comorbidity;

This suggestion has been taken into account when reformulating the objective.

3- The section on the study design is not well presented and must be rewritten as well as the description of the study site. This description targeted schistosomiasis without any information linked to alcohol consumption in this endemic area;

These sections have been rewritten according to your instructions.

4- The focus of the study was the comorbidity between schistosomiasis and alcoholism. However, the section treating this comorbidity is not enough.

The section treating comorbidity has been expanded.

The comparison between KK and POC-CCA is not important because several studies have already compared these two tests, even in the same endemic area (see Mewamba et al. 2022). Moreover, the sample size does enable to have reliable results on the performance (sensitivity, specificity, and accuracy) of each diagnostic test. The goal standard test was not provided.

The comparison between diagnostic methods has been removed from the revised manuscript.

Risk factors associated with schistosomiasis and alcoholism are well known; but risk factors associated with comorbidity are of great interest.

Risk factors associated with schistosomiasis and alcohol abuse have been added to the results.

5- For the calculation of the sample size, the schistosomiasis prevalence was used. However, the authors have forgotten that they are dealing with schistosomiasis and alcoholism. Moreover, the disease prevalence used was too old.

Details related to this concern have been provided previously, and adjustments have been made in the revised manuscript.

6- In the MM, the sections treating risk behaviors to schistosomiasis and factors associated with alcohol abuse must be part of the questionnaire

Effectively, risk behaviors to schistosomiasis and factors associated with alcohol abuse are part of the questionnaire. The section “Questionnaire” is the level 2 heading, whereas “Socio-environmental characteristics”, “Assessment of alcohol consumption level”, “Determination of risk behaviors for *Schistosoma mansoni* infection”, and “Determination of factors associated with alcohol abuse” are level 3 headings (sub-sections).

7- The result section must be rewritten, synthesized, and focused on the main objective that treats the comorbidity.

Your remarks have been considered, and adjustments have been made in the revised manuscript.

8- The discussion must be rewritten. It must be related to the road map objective of elimination schistosomiasis as public health. The authors must discuss the high prevalence of schistosomiasis in adults in relationship with the current control strategies.

Your suggestions have been considered, and corrections have been made in the revised manuscript.

9- How data on comorbidity could help to improve disease control in neighborhoods. The relationship between comorbidity and liver disease was not discussed.

These points have been discussed in the revised manuscript.

10- The limitations are not related to week points of the study. For instance, without investigations on the liver diseases to know its prevalence, it will be difficult to infer the impact of schistosomiasis or alcoholism and their comorbidity on liver disease.

We thank you for this insightful comment.

Our study was primarily designed to investigate the association between *S. mansoni* infection and alcohol consumption patterns, not to determine the prevalence of liver diseases. Further studies, including clinical, physiological, and imaging assessments of liver diseases, would be necessary to evaluate the impact of schistosomiasis and/or alcoholism and their comorbidity on liver diseases.

Minor comments
- There is a need to clarify the terms “alcoholism” and “alcohol abuse”. Are these term similar in the context of this study?

We have retained the expression "alcohol abuse" and have harmonized it throughout the revised manuscript.

References giving the schistosomiasis prevalence in this area are too old (>25 years). Recent data on this prevalence have been generated (see Mewamba et al., 2022).

Data in lines 201-203 must be moved to the result section.

Details related to this concern have been provided above, and corrections have been made in the revised manuscript.

The number of tables must be reduced;

We acknowledge the reviewer's suggestion regarding the number of tables. However, all the tables are important to ensure a better understanding of the complex interactions between clinical and behavioral variables.

Table 10: How did the authors identify the transmission sites? Did they have data reporting the schistosomiasis transmission sites in different neighborhoods? With clear study highlighting such sites, mentioning the transmission site is not justified. In the same table, itching and skin rash were also mentioned as variables. Are these variables some clinical signs of schistosomiasis and/or alcoholism?

Several previous projects conducted by our research team have taken place in these neighborhoods, and we are therefore very familiar with the transmission sites. The itching and skin rash reported in Table 10 are clinical manifestations of schistosomiasis.

- Results contained in lines 438-443 have been repeated in lines 471 – 476.

We thank the reviewer for this observation. However, we would like to clarify that the results presented in lines 438-443 and 471-476 do not constitute a repetition. The first section (lines 438-443) reports the findings of the univariate analysis (Table 9), whereas the second section (lines 471-476) details the multivariate binary logistic regression analysis (Table 11) of all significant variables (p < 0.05) from the univariate analysis. This presentation is necessary to identify independent risk factors.

- How did the authors distinguish adulterated whisky from other type of whisky? Was it not possible to have also adulterated wine or beer?

In the context of this study, the expression “adulterated whisky” refers to all whiskies packaged in plastic sachets and marketed in Cameroon. This classification is based on the fact that these products have been subject to multiple sales bans by competent authorities due to their uncontrolled composition and proven toxicity. Regarding beer and wine, the products consumed in our study area primarily originated from regulated industrial distribution channels.

Overall, this manuscript contains a lot of short-come and it can be accepted for publication in Plos NTD
