# Supplementary material for: Schistosomiasis mansoni and alcohol abuse comorbidity: Prevalence and risk factors among adults in Makenene, Cameroon
Source: PLoS Negl Trop Dis. 2026 Jul 6;20(7):e0013687. doi: 10.1371/journal.pntd.0013687 (PMC13349307; doi:10.1371/journal.pntd.0013687)
Supplement: Attachment — Submitted filename: review report.docx [file pntd.0013687.s006.docx]

**Abstract:**

1. The conclusion and significance section would benefit from revision. While the need for interventions addressing both schistosomiasis and alcohol abuse is understandable, the priority should be to conduct additional studies that clarify the causal pathways and quantify the influence of each condition on the other. Without this evidence, it is difficult to justify integrated interventions as the primary recommendation. Moreover, the feasibility of such integrated approaches is unclear. Schistosomiasis control programs typically target specific high‑risk groups, and the authors’ own findings indicate that the highest likelihood of comorbidity occurs among individuals aged 18–39, an age group that may not align with the main targets of schistosomiasis control activities. Given these considerations, the conclusion and significance should be revised to reflect the need for stronger evidence and to address the practical challenges of implementing integrated interventions.
2. Key words: it is recommended not to have a key word longer made from more than 3 words. The last key word is almost a “key sentence”.

**Author Summary:**

1. Lines 47–49: The term co‑occurrence is more appropriate than comorbidity throughout the manuscript. Because the study does not investigate whether schistosomiasis and alcohol use are associated or whether one condition influences the other, referring to them as a comorbidity overstates the evidence. I recommend replacing comorbidity with co‑occurrence to more accurately reflect the descriptive nature of the findings.
2. Lines 55–58: The feasibility of integrating schistosomiasis control with alcohol‑related interventions is questionable. Mass drug administration for schistosomiasis is typically directed toward well‑defined high‑risk groups, which may not overlap with the populations most affected by alcohol misuse

**Introduction:**

1. Lines 94-97: As suggested above, the term co-occurrence should be used throughout the manuscript

**Material and Methods:**

1. Lines 151–155: It is unclear why a single neighborhood within a community/village was analyzed separately. This approach is confusing and may introduce bias. The study should clearly define the evaluation unit and apply it consistently across all sites. If the intended unit is the community/village, then all three villages described in lines 151–152 should be treated as such. If the intended unit is the neighborhood, then all villages should be subdivided into neighborhoods and analyzed accordingly. Mixing units of analysis without justification undermines comparability.
2. Lines 235-240: repetition of lines 228-230.
3. Lines 231–235: What explains the discrepancy between the number of stool and urine samples? In addition, given the objective of the study, what was the rationale for using two schistosomiasis diagnostic methods?
4. While it was a good idea to provide a flow chart (Figure 1), the one provided it was not so informative, I suggest revising it to make it clearer. It might be important to include the number of individuals that were considered for risk factors analysis.
5. Lines 272-273: why were both Shapiro wilk and Kolmogorov-Smirnov used to assess normality?

**Results:**

1. Table 1: in the methods, Baloua is considered a neighborhood of Mock Centre, be here it is presented as community. Please clarify this and be consistent with your unit of evaluation.
2. I suggest adding an asterisk to ‘Other’ to indicate that an explanation is provided below
3. Line 337-338: “However, no significant difference was observed at the community level (p = 0.608)”, do you mind no significant difference was observed between communities?
4. In lines 436–441, the term “interaction” may not be appropriate. Demonstrating a true interaction requires a specific study design and statistical analysis. Using “association” or “relationship” would be more accurate and better aligned with the analyses presented.

**Discussion:**

1. Lines 637–641: Comparing prevalence in adults with that observed in school‑aged children is not appropriate. Rather than reflecting the impact of school‑based MDA, the high prevalence among adults suggests that untreated adults may undermine elimination efforts. This highlights the need to extend MDA coverage to adults.

**Conclusion:**

1. See the previous comment in the abstract section
2. Line 868: There was no interaction assessment. As noted above, the authors analyzed associations/relationships rather than interactions. In this specific sentence, the term ‘co‑existence’ may be more appropriate.
