# Supplementary material for: Schistosomiasis mansoni and alcohol abuse comorbidity: Prevalence and risk factors among adults in Makenene, Cameroon
Source: PLoS Negl Trop Dis. 2026 Jul 6;20(7):e0013687. doi: 10.1371/journal.pntd.0013687 (PMC13349307; doi:10.1371/journal.pntd.0013687)
Supplement: Attachment — Submitted filename: Response_to_reviewers_auresp_2.docx [file pntd.0013687.s007.docx]

**ANSWERS TO REVIEWERS’ COMMENTS**

**Manuscript PNTD-D-26-00185**

**Title: Schistosomiasis mansoni and alcohol abuse comorbidity: prevalence and risk factors among adults in Makenene, Cameroon**

**I- Methods**
-Are the objectives of the study clearly articulated with a clear testable hypothesis stated?
-Is the study design appropriate to address the stated objectives?
-Is the population clearly described and appropriate for the hypothesis being tested?
-Is the sample size sufficient to ensure adequate power to address the hypothesis being tested?
-Were correct statistical analysis used to support conclusions?
-Are there concerns about ethical or regulatory requirements being met?

**Reviewer #1**:

The study is novel and addresses an important and underexplored intersection between two significant public health problems intestinal schistosomiasis and alcohol abuse. The objectives are clearly articulated, specifically aiming to investigate the prevalence and risk factors associated with *Schistosoma mansoni* infection and alcohol abuse comorbidity in Makenene, Cameroon.

The justification provided adequately supports the chosen research design, and the sample size appears sufficient to ensure appropriate statistical power to address the study objectives.

The study demonstrates adherence to ethical and regulatory standards, which strengthens its credibility. While the analytical approach is comprehensive, I have clearly indicated my limitations in undertaking a detailed and extensive statistical review of the analyses conducted.

Thank you for your appreciation

**Reviewer #2**:

The manuscript meets all key review criteria for acceptance. The authors have thoroughly addressed all reviewer critiques, including adding new analyses (comorbidity risk factors in Table 17), correcting statistical methods (median/IQR, normality testing), expanding limitations, and strengthening the discussion of clinical and public health implications.

Thank you for your appreciation

**Reviewer #3:** (No Response)

**Reviewer #4:** (No Response)

**II- Results**
-Does the analysis presented match the analysis plan?
-Are the results clearly and completely presented?
-Are the figures (Tables, Images) of sufficient quality for clarity?

**Reviewer #1:**

The results are presented clearly and effectively, particularly through well-structured tables that are concise, not overloaded, and easy to interpret. This enhances readability and allows for straightforward understanding of key findings. The quality of the tables is commendable and supports the overall clarity of the manuscript.

Thank you for this positive assessment of our results presentation.

**Reviewer #2:**

The results section meets PLOS NTD standards for acceptance. The authors have:

• Presented all findings systematically

• Included appropriate statistical measures (OR, AOR, 95% CI, p-values)

• Added the requested interaction analysis (Table 8)

• Added the requested comorbidity risk factor analysis (Table 17)

• Removed off-topic diagnostic comparison sections

• Ensured all tables are clear and properly labeled

Therefore, no further revisions to the results section are required.

We thank you for this positive evaluation of the results section and for acknowledging the revisions made to the manuscript.

**Reviewer #3: (No Response)**

**Reviewer #4: (No Response)**

**III- Conclusions**
-Are the conclusions supported by the data presented?
-Are the limitations of analysis clearly described?
-Do the authors discuss how these data can be helpful to advance our understanding of the topic under study?
-Is public health relevance addressed?

**Reviewer #1:**

The findings convincingly highlight the concomitance of intestinal schistosomiasis and alcohol abuse, underscoring the compounded risk these conditions pose, particularly in relation to liver morbidity. The results contribute valuable evidence to an area that has received limited attention and provide a strong basis for the discussion and conclusions drawn.

The study fully demonstrates that the coexistence of intestinal schistosomiasis and alcohol abuse represents a significant and potentially underrecognized public health concern. The paper effectively highlights the risk of exacerbated liver complications resulting from this comorbidity, particularly in endemic settings such as Cameroon.

By drawing attention to the dual burden of infectious and behavioral risk factors, the study makes an important contribution to the scientific literature and underscores the need for integrated approaches to disease control and health promotion.

The manuscript makes a compelling case for integrating alcohol abuse prevention strategies into schistosomiasis control programs. This integrated approach has the potential to enhance the effectiveness of public health interventions aimed at reducing disease burden and preventing complications.

Given its novelty, relevance, and overall quality, the study should receive attention within the scientific and public health communities.

We are grateful for this positive and encouraging evaluation of our work.

**Reviewer #2:**

The conclusions section meets all PLOS NTD requirements for acceptance. The authors have:

1. Drawn conclusions directly supported by their data. All conclusions were derived directly from the presented results (Tables 2, 5, 7, 17).

2. Provided a thorough limitations section addressing key methodological concerns. Example: a comprehensive limitations section (lines 844-859) covering diagnostic, self-report, generalizability, and unmeasured confounders.

3. Clearly articulated how their findings advance understanding of schistosomiasis-alcohol comorbidity through novel findings on community-specific dynamics, synergistic pathology, and praziquantel metabolism concerns.

4. Connected their findings to specific public health interventions and global elimination targets. This was through specific recommendations for integrated interventions, adult inclusion in MDA, and alignment with the WHO roadmap.

Recommendation: Accept as is, with optional minor clarification regarding adulterated whisky in the conclusion.

Thank you for this positive assessment and recommendation. The suggested correction regarding adulterated whisky has been considered in the revised manuscript. We removed “adulterated whisky” in the conclusion (line 1035)

**Reviewer #3: (No Response)**

**Reviewer #4: (No Response)**

**IV- Editorial and Data Presentation Modifications?**

Use this section for editorial suggestions as well as relatively minor modifications of existing data that would enhance clarity. If the only modifications needed are minor and/or editorial, you may wish to recommend “Minor Revision” or “Accept”.

**Reviewer #1:**

Accept.

Thank you for your positive evaluation.

**Reviewer #2:**

The manuscript has been thoroughly revised, and all substantive reviewer concerns have been addressed. Only minor editorial issues remain, none of which affect scientific validity or clarity. Therefore, the paper is suitable for publication in PLOS Neglected Tropical Diseases.

Minor Editorial Suggestions (Optional, Not Required for Acceptance)

The following suggestions would further enhance clarity and consistency, but do not necessitate another round of review.

1. Standardize decimal separator

Inconsistent use of comma and period as decimal separator (e.g., "40,7%" in line 173 vs. "40.7%" elsewhere). Please check line 173 (sample size calculation). It states, "estimated at 40,7% in 2019 [8]". I suggest it as "estimated at 40.7% in 2019 [8]".

Thank you. The correction has been done (line 913).

2. Clarify the adulterated whisky mentioned in the conclusion

The conclusion states that regular consumption of adulterated whisky exacerbates the dual health burden, but the discussion clarifies that this beverage was not significantly associated with comorbidity (only with alcohol abuse). See conclusion, lines 864-866. You indicated that "regular consumption of adulterated whisky, palm wine, and beer... exacerbates this dual health burden".

Ideally, it should be: "regular consumption of palm wine and beer, along with factors associated with agricultural activities and frequent human-contaminated water sources, exacerbates this dual health burden" (Remove "adulterated whisky" from the conclusion since it was not a significant comorbidity predictor; keep it in the discussion where the nuanced finding is explained.).

Note: this improves accuracy and consistency between the conclusion and discussion.

Thank you for this remark. We adopted your suggestion (line 1035).

3. Standardize "Kato-Katz" hyphenation

In some instances, use "Kato Katz" without hyphen. See Table 8 footnote: "diagnosed by the Kato Katz". I suggest you change to "Kato-Katz" throughout.

Thank you. “Kato-Katz” has been harmonized throughout the manuscript according to your recommendation (lines 443 and 631).

4. Table formatting for production

Several tables (e.g., Tables 7, 9, 10) contain line breaks within cells that may need adjustment during typesetting. This is normal and will be handled by the production team. No action needed from authors.

Overall Recommendation: Accept.

Thank you, for your overall appreciation.

**Reviewer #3: (No Response)**

**Reviewer #4: (No Response)**

**V- Summary and General Comments**
Use this section to provide overall comments, discuss strengths/weaknesses of the study, novelty, significance, general execution and scholarship. You may also include additional comments for the author, including concerns about dual publication, research ethics, or publication ethics. If requesting major revision, please articulate the new experiments that are needed.

**Reviewer #1:**

The study fully demonstrates that the coexistence of intestinal schistosomiasis and alcohol abuse represents a significant and potentially underrecognized public health concern. The paper effectively highlights the risk of exacerbated liver complications resulting from this comorbidity, particularly in endemic settings such as Cameroon.

By drawing attention to the dual burden of infectious and behavioral risk factors, the study makes an important contribution to the scientific literature and underscores the need for integrated approaches to disease control and health promotion.

The manuscript makes a compelling case for integrating alcohol abuse prevention strategies into schistosomiasis control programs. This integrated approach has the potential to enhance the effectiveness of public health interventions aimed at reducing disease burden and preventing complications.

Given its novelty, relevance, and overall quality, the study should receive attention within the scientific and public health communities.

**Reviewer #2:**

1. This manuscript by Yimgoua and colleagues presents a novel, well-executed, and methodologically sound investigation of the comorbidity between Schistosoma mansoni infection and alcohol abuse among adults in Makenene, Cameroon. The study addresses a significant gap in the literature by examining the intersection of an infectious disease (schistosomiasis) and a behavioral risk factor (alcohol misuse) that share a common target organ, the liver, yet have rarely been studied together in endemic populations.

2. The authors have responded comprehensively to all four reviewers' critiques, resulting in a substantially improved manuscript. Key revisions include the following: (1) addition of an explicit testable hypothesis; (2) removal of extraneous diagnostic test comparisons to focus on comorbidity; (3) correction of statistical methods (median/IQR for non-normal data; geometric mean for EPG); (4) new multivariate analysis of factors associated with comorbidity (Table 17); (5) interaction analysis between parasite burden and alcohol use (Table 8); (6) expanded limitations section; and (7) strengthened discussion of clinical and public health implications, including potential synergistic hepatic damage and effects on praziquantel metabolism.

**Reviewer #3: (No Response)**

**Reviewer #4: (No Response)**
