# Supplementary material for: Schistosomiasis mansoni and alcohol abuse comorbidity: Prevalence and risk factors among adults in Makenene, Cameroon
Source: PLoS Negl Trop Dis. 2026 Jul 6;20(7):e0013687. doi: 10.1371/journal.pntd.0013687 (PMC13349307; doi:10.1371/journal.pntd.0013687)
Supplement: Attachment — Submitted filename: Responses to Reviewer #4.docx [file pntd.0013687.s008.docx]

**ANSWERS TO REVIEWERS’ COMMENTS**

**Manuscript PNTD-D-26-00185**

**Title: Schistosomiasis mansoni and alcohol abuse comorbidity: prevalence and risk factors among adults in Makenene, Cameroon**

**Reviewer #4**

This manuscript PNTD-D-25-01931 of Yimgoua et al entitled "Schistosomiasis mansoni-alcoholism comorbidity: prevalence and risk factors among adults in Makenene, Cameroon" treat an interesting topic related to intestinal schistosomiasis and alcoholism as well as their comorbitity in schistosomiasis endemic area of Makenene in Cameroon.

We thank the reviewer #4 for their valuable comments and suggestions.

We would like to clarify that the manuscript version we will use to implement the requested corrections is the most recent version we submitted, which already incorporates the comments and suggestions provided by the other reviewers and the editor.

Major comment

The manuscript has been considerably improved. However, there is still some corrections to made notably the tables. The manuscripts contains 17 tables. This is too much and there is a need to reduced considerably the number of tables.

We thank the reviewer for this observation.

In response to your recommendation and to streamline the presentation, we have carefully re-evaluated all data and removed two tables from the most recent version (**Table 2 and Table 8**).

Regarding the remaining tables, this study integrates three distinct dimensions:

- A baseline epidemiological data regarding intestinal schistosomiasis,
- A comprehensive behavioral data on alcohol abuse patterns,
- A detailed cross-analysis of the comorbidity itself.

While this exhaustive approach displays substantial data, each remaining table provides essential information for a complete understanding of this epidemiological profile. We have therefore retained this presentation to preserve the clarity, scientific completeness, and reproducibility of our findings.

Minor points

1- Lines 29 to 31: The prevalence was given for the two techniques used, but it is not clear how the final prevalence was obtained

We thank the reviewer for this observation.

The precision has been added to the abstract of the revised manuscript: participants were considered *S. mansoni*-positive if either diagnostic test (Kato-Katz or POC-CCA) was positive (**lines 27 - 28**).

2- Line 151 -153: is there a difference between village and neighborhood? Did the authors consider Mock sud and Mock centre as villages or neighbordhood? Ii Baloua a village or a neighbordhood?

We thank the reviewer for this observation.

Administratively, Mock Centre, Mock Sud, and Carrière are classified as distinct villages, whereas Baloua is a neighborhood nested within the village of Mock Centre.

However, from a methodological standpoint, Baloua was treated as an independent study site at the same level as the three main villages. Because of its immediate geographical proximity to the Mock River and its historically documented hyper-endemicity, Baloua faced distinct transmission dynamics that justified isolating its data. Consequently, the community-based component of this study included four distinct study sites: Mock Centre, Mock Sud, Carrière, and Baloua.

To avoid any ambiguity, we rephrased this section in the revised manuscript. (**lines 756 – 761** in the revised manuscript).

3- Lines 232-235; Must move to the result section

We appreciate the reviewer's guidance.

We understand that sample counts can often be perceived as results. However, these lines were intended to define the procedural flow of sample collection and to establish the technical basis of the study cohort, as illustrated by the flow chart in this section (Figure 1). Keeping this breakdown within the Methodology section ensures that the reader can immediately grasp the sampling dynamics. (**lines 835 – 837** in the revised manuscript).

4- Lines 235- 238: Repetition (see lines 228 - 230)

We thank the reviewer for identifying this redundancy.

The redundant sentence has been removed. (**lines 834 – 836** in the most recent version).

5- Lines 234-235: sentence to be rephrased

We thank the reviewer for this recommendation.

The sentence has been rephrased in the revised manuscript to improve clarity. (**lines 838 –839** in the revised manuscript)

6- Line 248: What do the authors mean by “Kato solution”?

We thank the reviewer for this observation.

To ensure full methodological clarity, the expression "Kato solution" has been replaced with "glycerol-malachite green solution" in the revised manuscript. (**line 852** in the revised manuscript)

7- Line 271: “middle adulthood (50 – 59 years old)”: it must be (40-59 years old)

We thank the reviewer for this remark.

The age range has been changed to "40–59 years old" in the revised manuscript. (**line 875** in the revised manuscript)

8- Lines 310-312: The is a need of some clarifications: 109 POCCAA+ + 75 KK+ = 184; will be good to clarify how many were positive for both tests. Table 2 can be deleted.

We thank the reviewer for this observation.

In accordance with your recommendation, Table 2 has been deleted. Moreover, the number of participants positive for both diagnostic methods has been added to this section in the revised manuscript (**lines 142 – 143** in the most recent version).

9- Lines 328 to 329 must be deleted

We thank the reviewer for this recommendation.

These lines (**lines 160 – 161** in the most recent version) have been deleted from the revised manuscript.

10- line 331: The first prevalence must be deleted; Moreove, “rate” which is in front of the second prevalence must be deleted

We thank the reviewer for this observation.

These corrections have been applied to the revised manuscript. (**line 161** in the most recent version)

11- Line 346: The “to” which is in front of “21.9% (95% CI: 12.9–31.4)” must be deleted

We thank the reviewer for this remark.

The word "to" has been successfully deleted from this sentence in the revised manuscript. (**line 176** in the most recent version)

12- Lines 425 to 427 must be deleted

We thank the reviewer for this recommendation.

These lines (**lines 222 – 224** in the most recent version) have been deleted from the revised manuscript.

13- Lines 467 to 468 must be deleted

We thank the reviewer for this recommendation.

These lines (**lines 250 – 251** in the most recent version) have been deleted from the revised manuscript.

14- Table 8 must be deleted

We understand the reviewer's recommendation.

Table 8 has been deleted. However, we reformulate the text to explain that in this study, alcohol consumption level was not associated with *S. mansoni* infection burden, and vice versa. (**Lines 298 – 304**).

This section was added in the previous version of the manuscript to provide answers to the reviewers' comments (Reviewer #2: "It cannot establish if alcohol abuse increases the risk of infection or vice versa." Reviewer #4: "The focus of the study was the comorbidity between schistosomiasis and alcoholism. However, the section treating this comorbidity is not enough." ).

15- Table 11: Data not available; This table can be deleted

We understand the reviewer's suggestion.

However, we respectfully request that Table 11 (**Table 9** in the revised manuscript) be maintained, as it represents the final output of the multivariate analysis.

We also want to clarify that the empty cells in the Adjusted Odds Ratio (AOR) column do not indicate missing or unavailable data. Rather, these blanks are the direct result of the stepwise backward elimination procedure applied. In this approach, the model starts with all potential variables and systematically removes them one by one based on their overall contribution to the model's fit. This process is specifically designed to control for confounding factors and eliminate multicollinearity. Table 11 (**Table 9** in the revised manuscript) accurately reflects this methodological process, showing which variables remained as the true independent predictors of *S. mansoni* infection in the study population.

16- Line 533 must be deleted

We thank the reviewer for this recommendation.

These lines (**lines 398 – 399** in the most recent version) have been deleted from the revised manuscript.

17 - Line 562 must be deleted

We thank the reviewer for this recommendation.

These lines (**line 426** in the most recent version) have been deleted from the revised manuscript.

18- Lines 568-570: Repetition of lines 508-510

We understand how these two passages might appear similar at first glance, as they both discuss the risk associated with different types of alcoholic beverages.

However, we would like to respectfully clarify that they report two entirely distinct statistical steps in the study protocol: lines 508 –510 (**lines 373 – 374** in the revised manuscript) describe the results of the univariate analysis, presenting the crude odds ratio (OR = 37.93 for adulterated whisky). Lines 568 –570 (**lines 431 – 433** in the revised manuscript) describe the results of the multivariate analysis, presenting the adjusted odds ratio (AOR = 26.84 for adulterated whisky) after controlling for confounding variables through the logistic regression model.

19- Lines 596-598: must be deleted

We thank the reviewer for this recommendation.

These lines (**lines 459 – 460** in the most recent version) have been deleted from the revised manuscript.

20- Line 632- 634: not clear what the authors want to highlight

We thank the reviewer for this observation.

The inclusion of this point was initially recommended during the first round of revision (“Reviewer #2: Clearly state that the POC-CCA sub-sample (n=165) is not representative of the entire population and has a different demographic profile, hence the different prevalence.”)

The sentence has been rephrased in the revised manuscript as follows: "The lower concordance between the overall prevalence and that of the POC-CCA sub-sample may reflect the fact that the POC-CCA sub-sample was not fully representative of the entire study population." (**lines 494 – 497** in the revised manuscript)

21- Line 637 641: Comparing the disease prevalence in adults and school-aged children is not relevant. The two groups of inhabitants are not subjected to level of exposition and slo to the same control measures.

We thank the reviewer for this comment.

Accordingly, we revised the manuscript to remove any direct comparison between the prevalence observed in adults and that previously reported among school-aged children.

The revised text now emphasizes that the present study provides the first estimate of *S. mansoni* prevalence among adults in Makenene. The prevalence data previously reported in school-aged children are now presented solely as historical epidemiological information documenting the long-standing endemicity of schistosomiasis in the locality, rather than as a basis for direct comparison with the adult population. (lines **499 – 505** in the revised manuscript)
