# Supplementary material for: Schistosomiasis mansoni and alcohol abuse comorbidity: Prevalence and risk factors among adults in Makenene, Cameroon
Source: PLoS Negl Trop Dis. 2026 Jul 6;20(7):e0013687. doi: 10.1371/journal.pntd.0013687 (PMC13349307; doi:10.1371/journal.pntd.0013687)
Supplement: Peer review history

# Peer review: Decision Letter 0

**DOI:** 10.1371/journal.pntd.0013687.r001

**Authors:** Hugues C. Nana Djeunga, Lucienne Tritten

13 Jan 2026

PNTD-D-25-01931

Schistosomiasis mansoni-alcoholism comorbidity: prevalence and risk factors among adults in Makenene, Cameroon

PLOS Neglected Tropical Diseases

Dear Dr. Boukeng Jatsa,

Thank you for submitting your manuscript to PLOS Neglected Tropical Diseases. After careful consideration, we feel that it has merit but does not fully meet PLOS Neglected Tropical Diseases's publication criteria as it currently stands. Therefore, we invite you to submit a revised version of the manuscript that addresses the points raised during the review process.

Please submit your revised manuscript within by March 13th 2026. If you will need more time than this to complete your revisions, please reply to this message or contact the journal office at plosntds@plos.org. When you're ready to submit your revision, log on to https://www.editorialmanager.com/pntd/ and select the 'Submissions Needing Revision' folder to locate your manuscript file.

Please include the following items when submitting your revised manuscript:

* A letter that responds to each point raised by the editor and reviewer(s). You should upload this letter as a separate file labeled 'Response to Reviewers'. This file does not need to include responses to any formatting updates and technical items listed in the 'Journal Requirements' section below.

* A marked-up copy of your manuscript that highlights changes made to the original version. You should upload this as a separate file labeled 'Revised Manuscript with Track Changes'.

* An unmarked version of your revised paper without tracked changes. You should upload this as a separate file labeled 'Manuscript'.

If you would like to make changes to your financial disclosure, competing interests statement, or data availability statement, please make these updates within the submission form at the time of resubmission. Guidelines for resubmitting your figure files are available below the reviewer comments at the end of this letter.

We look forward to receiving your revised manuscript.

Kind regards,

Hugues C. Nana Djeunga, PhD

Academic Editor

PLOS Neglected Tropical Diseases

Lucienne Tritten

Section Editor

PLOS Neglected Tropical Diseases

Shaden Kamhawi

co-Editor-in-Chief

PLOS Neglected Tropical Diseases

orcid.org/0000-0003-4304-636XX

Paul Brindley

co-Editor-in-Chief

PLOS Neglected Tropical Diseases

orcid.org/0000-0003-1765-0002

**Additional Editor Comments:**

The manuscript was evaluated by four reviewers and will require substantial revisions throughout. In particular, the study objectives and overall design need to be more clearly defined, and the justification of the sample size requires improvement. Several aspects of the Results and Discussion sections also need to be strengthened.

**Journal Requirements:**

If the reviewer comments include a recommendation to cite specific previously published works, please review and evaluate these publications to determine whether they are relevant and should be cited. There is no requirement to cite these works unless the editor has indicated otherwise.

1) We note that your Data Availability Statement is currently as follows: "N/A". Please confirm at this time whether or not your submission contains all raw data required to replicate the results of your study. Authors must share the “minimal data set” for their submission. PLOS defines the minimal data set to consist of the data required to replicate all study findings reported in the article, as well as related metadata and methods (https://journals.plos.org/plosone/s/data-availability#loc-minimal-data-set-definition).

For example, authors should submit the following data:

1) The values behind the means, standard deviations and other measures reported;

2) The values used to build graphs;

3) The points extracted from images for analysis..

Authors do not need to submit their entire data set if only a portion of the data was used in the reported study.

If your submission does not contain these data, please either upload them as Supporting Information files or deposit them to a stable, public repository and provide us with the relevant URLs, DOIs, or accession numbers. For a list of recommended repositories, please see https://journals.plos.org/plosone/s/recommended-repositories.

If there are ethical or legal restrictions on sharing a de-identified data set, please explain them in detail (e.g., data contain potentially sensitive information, data are owned by a third-party organization, etc.) and who has imposed them (e.g., an ethics committee). Please also provide contact information for a data access committee, ethics committee, or other institutional body to which data requests may be sent. If data are owned by a third party, please indicate how others may request data access.

**Reviewers' Comments:**

Reviewer's Responses to Questions

**Key Review Criteria Required for Acceptance?**

As you describe the new analyses required for acceptance, please consider the following:

**Methods**

-Are the objectives of the study clearly articulated with a clear testable hypothesis stated?

-Is the study design appropriate to address the stated objectives?

-Is the population clearly described and appropriate for the hypothesis being tested?

-Is the sample size sufficient to ensure adequate power to address the hypothesis being tested?

-Were correct statistical analysis used to support conclusions?

-Are there concerns about ethical or regulatory requirements being met?

Reviewer #1: The manuscript investigates the prevalence and risk of Schistosomiasis mansoni infection and alcoholism among adults in Makenene in Cameroon. The objectives of the study is clearly stated together with the justification of the study. As a cross-sectional study the method is justified with adequate sample size selection and power to enable the correct statistical anaylsis of the. The study design and methods applied in estimating the prevalence of schistosomiasis and alcoholism are standard and acceptable.

Reviewer #2: The following are the points of concern to be addressed in method section:

1. Sample Size Calculation (Lines 143-148): The calculation uses a prevalence (p=41%) from 2010. Please justify why this older estimate was used and discuss whether this might affect the precision of your current estimates. Indicate whether a design effect was considered in the community-based sampling.

2. Diagnostic Approach (Lines 201-205, Fig 1): The algorithm for defining an overall infection status (positive by KK or POC-CCA) is acceptable given POC-CCA's higher sensitivity. However, this leads to a situation where the "overall" prevalence (34.6%) is lower than the POC-CCA prevalence in its sub-sample (66.1%). This needs careful explanation in the results (Lines 267-273) to avoid confusion. Clearly state that the POC-CCA sub-sample (n=165) is not representative of the entire population and has a different demographic profile, hence the different prevalence.

3. Statistical Analysis: The use of a binary logistic regression model is appropriate. For Table 11 and Table 16, please indicate which category served as the reference for each variable in the multivariate model (e.g., Age: ≥60 years as ref; Community: perhaps Mock Sud or the largest group?).

Reviewer #3: 1. Lines 31–33: The comparison between Kato-Katz and POC-CCA, although scientifically relevant, appears outside the scope of the current manuscript.

2. Lines 198–199: Please specify the exact transportation conditions for the samples. Were they stored in a cooler with ice or kept at ambient temperature?

3. Lines 201–205: Clarify the rationale for using both Kato-Katz and POC-CCA. Since Kato-Katz is considered the gold standard, why was POC-CCA also included? If there is a specific justification, please state it clearly.

4. Lines 239–244: This comparison between diagnostic methods is not central to the study’s objectives and may distract the reader. Unless it adds novel value, consider removing it or presenting it separately.

5. Lines 260–261: Was a normality test performed to assess whether the data were normally distributed? If not, demographic variables should be presented using median and interquartile range.

6. General: Were normality tests applied to all variables? Many variables are presented as mean ± SD, which assumes normal distribution.

Reviewer #4: 1- The objective of the study is not clear enough. My suggestion is “determine the prevalence of schistosomiasis-alcoholism or alcohol abuse among adults in intestinal schistosomiasis endemic area of Makenene in Cameroon as well as the risk factors associated with this comorbidity;

2- The section on the study design is not well presented and must be rewritten as well as the description of the study site. This description targeted schistosomiasis without any information linked to alcohol consumption in this endemic area;

3- For the calculation of the sample size, the schistosomiasis prevalence was used. However, the authors have forgotten that they are dealing with schistosomiasis and alcoholism. Moreover, the disease prevalence used was too old.

4- In the MM, the sections treating risk behaviors to schistosomiasis and factors associated with alcohol abuse must be part of the questionnaire

**********

**Results**

-Does the analysis presented match the analysis plan?

-Are the results clearly and completely presented?

-Are the figures (Tables, Images) of sufficient quality for clarity?

Reviewer #1: The results, as presented, and the analysis of results conducted match the plan and are quite easy to follow. I am, however, a bit constrained in my understanding of some of the statistical analyses conducted. The tables are, clear, though some of the tables are loaded with too much information and could be simplified.

Reviewer #2: In the result section of this manuscript, the following should be addressed accordingly:

1. Table 2 & Corresponding Text: As above, the discrepancy between the high POC-CCA sub-sample prevalence and the lower combined prevalence requires a clear explanatory sentence.

2. Table 4 (Diagnostic Concordance): The calculations and presentation are good. It would be helpful to briefly state in the text that the 100% specificity for KK is due to the absence of KK+/POC-CCA- cases.

3. Table 7 & Text on Alcohol Abuse by Age: The text (Lines 387-390) states prevalence was higher in 40-59 year olds (p=0.032), but the AUDIT score did not differ (p=0.115). This is an interesting nuance. Ensure the discussion comments on why intensity (score) might be similar across ages, but the prevalence of "abuse" (AUDIT≥7) peaks in middle age.

4. Comorbidity (Section 3.4): The analysis is precise. Consider adding a simple cross-tabulation in the supplement showing the distribution of the four groups (S.m+/Alc+, S.m+/Alc-, S.m-/Alc+, S.m-/Alc-) for the whole cohort.

Reviewer #3: 1. Lines 38–40: Provide odds ratios with their 95% confidence intervals for clarity.

2. Lines 56–57: While relevant, the next step should be to investigate how one condition influences the other.

3. Lines 58–59: Clarify how this finding could inform targeted interventions.

4. Lines 172–174: Remove the commas before and after “as well as.”

5. Table 1: Clarify what is included in the category “Other.”

6. Table 2: This table appears unnecessary, as all information is already described in the text.

7. Table 3: Consider using a scatter plot for better visualization.

8. Lines 288–313: This section does not contribute to the study’s main objectives and could be removed.

9. Table 5: A graphical representation may be more effective (histogram for prevalence, scatter plot for intensity).

10. Lines 409–415: This section does not provide new information. A more informative analysis would compare S. mansoni infection intensity between alcohol abusers and non-abusers, or compare AUDIT scores between infected and uninfected participants.

11. Lines 420–424: Similar to previous comparisons; see comment 16.

12. Lines 430–434: See comment 16.

13. Lines 438–443: Consider replacing chi square values with odds ratios and 95% CIs for more meaningful interpretation.

14. Lines 492–496: Provide possible explanations for this finding. For example, differences in urbanization, alcohol availability, or affordability across communities.

15. Table 12: It is unclear which community was used as the reference category in the regression model. Since all communities show significant associations with alcohol abuse, clarification is essential for proper interpretation.

16. Improve Figure 1’s quality

Reviewer #4: The focus of the study was the comorbidity between schistosomiasis and alcoholism. However, the section treating this comorbidity is not enough. The comparison between KK and POC-CCA is not important because several studies have already compared these two tests, even in the same endemic area (see Mewamba et al. 2022). Moreover, the sample size does enable to have reliable results on the performance (sensitivity, specificity, and accuracy) of each diagnostic test. The goal standard test was not provided. Risk factors associated with schistosomiasis and alcoholism are well known; but risk factors associated with comorbidity are of great interest.

The result section must be rewritten, synthesized and focused on the main objective that treats the comorbidity.

**********

**Conclusions**

-Are the conclusions supported by the data presented?

-Are the limitations of analysis clearly described?

-Do the authors discuss how these data can be helpful to advance our understanding of the topic under study?

-Is public health relevance addressed?

Reviewer #1: The conclusions directly support the data presented, and limitations are clearly noted in the discussions. The conclusions and recommendations raise questions for further research.

Reviewer #2: The conclusion section indicates that the authors identify two key limitations: (1) diagnostic heterogeneity (use of two tests) and (2) reliance on self-reported data subject to bias. This is good, but it should be expanded.

1. The diagnostic limitation should explicitly state that the composite "gold standard" (either test positive) is imperfect, as POC-CCA may yield false positives, potentially inflating the "true" comorbidity rate.

2. A major limitation not discussed is the cross-sectional design, which precludes determining causality or the temporal relationship between risk factors and outcomes. It cannot establish if alcohol abuse increases the risk of infection or vice versa.

3. The potential for residual confounding caused by unmeasured variables (e.g., detailed socioeconomic status, access to healthcare) should be recognized.

4. The generalizability of findings beyond similar semi-rural, endemic foci could be briefly mentioned.

Reviewer #3: 1. Lines 728–729: Consider adding “or overestimation.”

2. Conclusion section: It is unclear how controlling alcohol consumption would advance schistosomiasis control, especially since the manuscript does not demonstrate a direct relationship between alcohol use and schistosomiasis risk. This section should be revised for clarity.

Reviewer #4: - The discussion must be rewritten. It must be related to the road map objective of elimination schistosomiasis as public health. The authors must discuss the high prevalence of schistosomiasis in adults in relationship with the current control strategies.

- How data on comorbidity could help to improve disease control in neighborhoods. The relationship between comorbidity and liver disease was not discussed.

**********

**Editorial and Data Presentation Modifications?**

Use this section for editorial suggestions as well as relatively minor modifications of existing data that would enhance clarity. If the only modifications needed are minor and/or editorial, you may wish to recommend “Minor Revision” or “Accept”.

Reviewer #1: The paper is well-written in standard English and would require no modifications in my opinion.

Reviewer #2: Recommendation: Minor Revision

The following suggestions aim to improve clarity, consistency, and conformity to journal formatting standards without changing the study's core methodology or findings.

1. Formatting & Journal Compliance

• Manuscript File: Prepare the main text as an editable document (e.g., .doc) with continuous line numbering, not as a PDF from the submission system.

• Headings: Ensure consistent formatting of headings (e.g., bold for main sections like Introduction and Methods).

• Financial Disclosure: Incorporate the full, correctly formatted funding statement into the main manuscript under a "Funding" section, including grant numbers and funder URLs.

• Data Availability: Replace "All relevant data are within the manuscript" with a statement compliant with PLOS policy. Example: "The data underlying the results presented in this study are available from [Repository Name, e.g., Dryad] at [DOI or URL]."

2. Terminology & Consistency

• Location Spelling: Standardize to one spelling for the study site throughout the manuscript (check abstract, title, and text). The PDF uses both "Makehene" and "Makenene".

• Diagnostic Tests: Consistently format test names: "Kato-Katz (KK)" and "point-of-care circulating cathodic antigen (POC-CCA)" on first use, then "KK" and "POC-CCA."

• Statistical Notation: Use proper superscripts (e.g., t² instead of t[2] in the sample size formula). Ensure consistent use of italics for Schistosoma mansoni.

3. Table & Figure Clarity

• Table 1: The table is clear. Ensure the final version avoids awkward page breaks.

• Table 2 & Text: Add a footnote to Table 2 or a sentence in the text explaining: "The overall prevalence was calculated for all participants (N=431), considering a participant positive if either diagnostic test was positive. The POC-CCA sub-sample (n=165) is not representative of the full cohort."

• Table 4 (Diagnostic Concordance): The table is excellent. Consider adding a brief parenthetical interpretation of the kappa value in the caption (e.g., "κ = 0.458, indicating moderate agreement").

• Figure 1: Include a clear yet brief legend within the main manuscript file (e.g., "Flowchart of participant enrolment and sample collection for Schistosoma mansoni diagnosis").

4. Language & Flow Enhancements

• Abstract, Line 23: Rephrase for clarity: "Considering that both schistosomiasis mansoni and alcoholism can induce liver pathology..."

• Methods, Sample Size (Lines 143-148): Improve sentence flow: "The minimum sample size was calculated using the formula N = t² * p * (1-p) / m², where *t* is the coefficient for a 95% confidence interval (1.96), *p* is the historical prevalence of intestinal schistosomiasis in Makehene (41%, from 2010), and *m* is the margin of error (5%). This yielded a minimum of 372 participants."

• Results, Section 3.1 (Lines 267-273): Add a transitional sentence: "Prevalence estimates varied by diagnostic method and sample. Among those providing urine... In contrast, the KK method applied to a larger stool sample group yielded... To generate an overall estimate for the entire cohort (N=431), a participant was considered infected if either test was positive, resulting in a prevalence of 34.6%."

• Discussion: Break up some long paragraphs (e.g., the paragraph spanning lines 620-670) into 2-3 focused paragraphs for easier reading.

5. Minor Corrections

• Author Summary, Line 49: Correct spelling to match chosen standard ("Makehene" or "Makenene").

• References: Perform a final check to ensure all in-text citations have a corresponding reference and that journal name abbreviations are consistent with PLOS style.

Finally,

Implementing these modifications will significantly improve the manuscript's presentation, professionalism, and readability, ensuring it aligns with journal standards and effectively communicates its important findings.

Reviewer #3: Minor Revision

Reviewer #4: (No Response)

**********

**Summary and General Comments**

Use this section to provide overall comments, discuss strengths/weaknesses of the study, novelty, significance, general execution and scholarship. You may also include additional comments for the author, including concerns about dual publication, research ethics, or publication ethics. If requesting major revision, please articulate the new experiments that are needed.

Reviewer #1: The topic of this manuscript is novel. I initially found it challenging to relate schistosomiasis infection and alcoholism but was fully convinced of the convergence after reading the background and study justification. I have no ethical concerns with the manuscript and readily recommend it for publication.

Reviewer #2: Summary and General Comments

Overall Assessment:

This manuscript presents a novel, important, and well-executed study that addresses a significant gap in public health research at the intersection of infectious and non-communicable diseases. The investigation of comorbidity between Schistosoma mansoni infection and alcohol use disorders in a semi-rural Cameroonian setting is both timely and relevant. The study is suitable for publication in PLOS Neglected Tropical Diseases following major revisions that primarily concern methodological clarification, enhanced interpretation, and strict adherence to the journal's formatting and data policies.

Strengths:

• High Novelty & Significance: This is the first documented study of this specific comorbidity in Cameroon (and likely in similar endemic settings). It moves beyond siloed disease perspectives, highlighting a critical syndemic that exacerbates liver disease burden and challenges existing control programs.

• Strong Methodology: The community-based, cross-sectional design is appropriate. The use of standardized diagnostic tools (KK, POC-CCA, AUDIT) is commendable. The sample size is adequate, and the statistical analyses (chi-square, logistic regression, and kappa statistics) are generally applied correctly.

• Clear Public Health Relevance: The findings have direct implications for integrated disease control strategies. The identification of shared socio-ecological risk factors and distinct behavioral drivers provides a concrete evidence base for designing multifaceted interventions.

• Rigorous Ethical Conduct: The study details robust ethical approvals, informed consent processes, provision of treatment, and post-study health education, meeting high ethical standards.

Weaknesses & Required Revisions:

The weaknesses are not fatal to the study's validity but require careful revision to ensure scientific rigor, clarity, and compliance.

1. Hypothesis & Objectives: While the objectives are clear, the introduction should be refined to include an explicit, testable primary hypothesis (e.g., concerning the expected prevalence of comorbidity and its key drivers).

2. Methodological Transparency:

a. Sample Size: Justify the sample size calculation more robustly, acknowledging its use for a comorbidity analysis and multivariate modeling.

b. Diagnostic Algorithm: Provide a much more straightforward explanation in the Results section for the discrepancy between the POC-CCA sub-sample prevalence and the final composite prevalence. Acknowledge the inherent limitations of the composite reference standard.

c. Statistical Reporting: Clearly state the reference categories for all variables in the multivariate logistic regression models (Tables 11, 16).

3. Discussion & Limitations:

a. Expand the Limitations section to explicitly discuss the constraints of the cross-sectional design (no causality), the potential for residual confounding, and the implications of the diagnostic approach.

b. Strengthen the discussion by briefly speculating on the potential synergistic clinical impact (e.g., on liver fibrosis progression) of the comorbidity.

c. Make the call for "integrated interventions" more tangible by suggesting 1-2 concrete, feasible examples (e.g., integrating AUDIT screening into schistosomiasis community outreach).

4. Journal Compliance (Critical):

a. Data Availability: The current statement is unacceptable. Data must be deposited in a public repository (e.g., Dryad, Figshare) with a DOI, and the statement must be updated to reflect this. This is a mandatory requirement for publication in PLOS journals.

b. Financial Disclosure: The funding statement must be completed in full within the manuscript text, following the journal's prescribed format.

c. Formatting: The manuscript must be reformatted to meet PLOS NTD style: include continuous line numbers, correct heading styles, and ensure figures are submitted separately with legends in the main text.

General Execution and Scholarship:

The scholarship is sound. The literature review is comprehensive, the data presentation is logical, and the writing is generally clear. The authors demonstrate a strong command of the subject matter. The required revisions are largely editorial and explanatory, aimed at elevating the manuscript's precision, transparency, and impact.

Ethics:

No concerns regarding dual publication, research ethics, or publication ethics were identified. The ethical oversight and procedures described are thorough and appropriate.

Conclusion:

This study makes a valuable and original contribution to the field. The requested revisions are essential to solidify the methodological narrative, provide a more critical self-assessment, and fulfill non-negotiable publishing criteria. I recommend "Major Revision" with the expectation that the authors can address these points through textual modifications, clarifications, and compliance updates without the need for new experiments or data re-analysis.

Reviewer #3: The manuscript “Schistosomiasis mansoni–alcoholism comorbidity: prevalence and risk factors among adults in Makenene, Cameroon” by Yimgoua et al. provides an update on the distribution and risk factors of intestinal schistosomiasis in several communities of a health district that is a traditional focus of schistosomiasis transmission in Cameroon. The authors also present valuable information on alcohol abuse prevalence and associated risk factors in the study area. Finally, they assess co infection patterns across communities and analyze them according to socio demographic characteristics.

the discussion would benefit from a paragraph on how this comorbidity may influence pathogenesis (schistosomiasis or liver function) and how excessive alcohol consumption may affect MDA outcomes (adverse events, drug efficacy). This would strengthen the public health relevance of the findings

Reviewer #4: Comments

This manuscript PNTD-D-25-01931 of Yimgoua et al entitled "Schistosomiasis mansoni-alcoholism comorbidity: prevalence and risk factors among adults in Makenene, Cameroon" treat an interesting topic related to intestinal schistosomiasis and alcoholism as well as their comorbidity in schistosomiasis endemic area of Makenene in Cameroon.

Major comment

The manuscript addressed two public health problems notably one of the most important neglected tropical disease and alcoholism in adults living intestinal schistosomiasis endemic area of Cameroon. Although the comorbidity between the two public health problem will be of great concern especially for the their potential of inducing liver diseases, this study has a lot of short-comes.

1- The introduction is not well written. It does not provide the state of the problem, previous studies on this comorbidity, the state of alcoholism in other schistosomiasis endemic areas, data on liver diseases related to schistosomiasis or alcoholism, the knowledge gaps related to this comorbidity;

2- The objective of the study is not clear enough. My suggestion is “determine the prevalence of schistosomiasis-alcoholism or alcohol abuse among adults in intestinal schistosomiasis endemic area of Makenene in Cameroon as well as the risk factors associated with this comorbidity;

3- The section on the study design is not well presented and must be rewritten as well as the description of the study site. This description targeted schistosomiasis without any information linked to alcohol consumption in this endemic area;

4- The focus of the study was the comorbidity between schistosomiasis and alcoholism. However, the section treating this comorbidity is not enough. The comparison between KK and POC-CCA is not important because several studies have already compared these two tests, even in the same endemic area (see Mewamba et al. 2022). Moreover, the sample size does enable to have reliable results on the performance (sensitivity, specificity, and accuracy) of each diagnostic test. The goal standard test was not provided. Risk factors associated with schistosomiasis and alcoholism are well known; but risk factors associated with comorbidity are of great interest.

5- For the calculation of the sample size, the schistosomiasis prevalence was used. However, the authors have forgotten that they are dealing with schistosomiasis and alcoholism. Moreover, the disease prevalence used was too old.

6- In the MM, the sections treating risk behaviors to schistosomiasis and factors associated with alcohol abuse must be part of the questionnaire

7- The result section must be rewritten, synthesized and focused on the main objective that treats the comorbidity.

8- The discussion must be rewritten. It must be related to the road map objective of elimination schistosomiasis as public health. The authors must discuss the high prevalence of schistosomiasis in adults in relationship with the current control strategies.

9- How data on comorbidity could help to improve disease control in neighborhoods. The relationship between comorbidity and liver disease was not discussed.

10- The limitations are not related to week points of the study. For instance, without investigations on the liver diseases to know its prevalence, it will be difficult to infer the impact of schistosomiasis or alcoholism and their comorbidity on liver disease.

Minor comments

- There is a need to clarify the terms “alcoholism” and “alcohol abuse”. Are these term similar in the context of this study?

- References giving the schistosomiasis prevalence in this area are too old (>25 years). Recent data on this prevalence have been generated (see Mewamba et al., 2022).

- Data in lines 201-203 must be moved to the result section.

- The number of tables must be reduced;

- Table 10: How did the authors identify the transmission sites? Did they have data reporting the schistosomiasis transmission sites in different neighborhoods? With clear study highlighting such sites, mentioning the transmission site is not justified. In the same table, itching and skin rash were also mentioned as variables. Are these variables some clinical signs of schistosomiasis and/or alcoholism?

- Results contained in lines 438-443 have been repeated in lines 471 – 476.

- How did the authors distinguish adulterated whisky from other type of whisky? Was it not possible to have also adulterated wine or beer?

Overall, this manuscript contains a lot of short-come and it can be accepted for publication in Plos NTD

**********

PLOS authors have the option to publish the peer review history of their article (what does this mean?). If published, this will include your full peer review and any attached files.

If you choose “no”, your identity will remain anonymous but your review may still be made public.

**Do you want your identity to be public for this peer review?**  For information about this choice, including consent withdrawal, please see our Privacy Policy.

Reviewer #1: **Yes:** Nana Kwadwo Biritwum

Reviewer #2: No

Reviewer #3: No

Reviewer #4: No

[NOTE: If reviewer comments were submitted as an attachment file, they will be attached to this email and accessible via the submission site. Please log into your account, locate the manuscript record, and check for the action link "View Attachments". If this link does not appear, there are no attachment files.]

**Figure resubmission:**

While revising your submission, we strongly recommend that you use PLOS’s NAAS tool (https://ngplosjournals.pagemajik.ai/artanalysis) to test your figure files. NAAS can convert your figure files to the TIFF file type and meet basic requirements (such as print size, resolution), or provide you with a report on issues that do not meet our requirements and that NAAS cannot fix.

After uploading your figures to PLOS’s NAAS tool - https://ngplosjournals.pagemajik.ai/artanalysis, NAAS will process the files provided and display the results in the "Uploaded Files" section of the page as the processing is complete. If the uploaded figures meet our requirements (or NAAS is able to fix the files to meet our requirements), the figure will be marked as "fixed" above. If NAAS is unable to fix the files, a red "failed" label will appear above. When NAAS has confirmed that the figure files meet our requirements, please download the file via the download option, and include these NAAS processed figure files when submitting your revised manuscript.

**Reproducibility:**

To enhance the reproducibility of your results, we recommend that authors of applicable studies deposit laboratory protocols in protocols.io, where a protocol can be assigned its own identifier (DOI) such that it can be cited independently in the future. Additionally, PLOS ONE offers an option to publish peer-reviewed clinical study protocols. Read more information on sharing protocols at https://plos.org/protocols?utm_medium=editorial-email&utm_source=authorletters&utm_campaign=protocols

Attachment Submitted filename: manuscript review.docx

# Author comment: Author response to Decision Letter 1

**DOI:** 10.1371/journal.pntd.0013687.r002

12 Mar 2026

Attachment Submitted filename: Response to reviewers.docx

# Peer review: Decision Letter 1

**DOI:** 10.1371/journal.pntd.0013687.r003

**Authors:** Hugues C. Nana Djeunga, Lucienne Tritten

7 May 2026

PNTD-D-25-01931R1

Schistosomiasis mansoni and alcohol abuse comorbidity: prevalence and risk factors among adults in Makenene, Cameroon

PLOS Neglected Tropical Diseases

Dear Dr. Boukeng Jatsa,

Thank you for submitting your manuscript to PLOS Neglected Tropical Diseases. After careful consideration, we feel that it has merit but does not fully meet PLOS Neglected Tropical Diseases's publication criteria as it currently stands. Therefore, we invite you to submit a revised version of the manuscript that addresses the points raised during the review process.

Please submit your revised manuscript by May 15. If you will need more time than this to complete your revisions, please reply to this message or contact the journal office at plosntds@plos.org. When you're ready to submit your revision, log on to https://www.editorialmanager.com/pntd/ and select the 'Submissions Needing Revision' folder to locate your manuscript file.

Please include the following items when submitting your revised manuscript:

* A letter that responds to each point raised by the editor and reviewer(s). You should upload this letter as a separate file labeled 'Response to Reviewers'. This file does not need to include responses to any formatting updates and technical items listed in the 'Journal Requirements' section below.

* A marked-up copy of your manuscript that highlights changes made to the original version. You should upload this as a separate file labeled 'Revised Manuscript with Track Changes'.

* An unmarked version of your revised paper without tracked changes. You should upload this as a separate file labeled 'Manuscript'.

If you would like to make changes to your financial disclosure, competing interests statement, or data availability statement, please make these updates within the submission form at the time of resubmission. Guidelines for resubmitting your figure files are available below the reviewer comments at the end of this letter.

As the corresponding author, your ORCID iD is verified in the submission system and will appear in the published article. PLOS supports the use of ORCID, and we encourage all coauthors to register for an ORCID iD and use it as well. Please encourage your coauthors to verify their ORCID iD within the submission system before final acceptance, as unverified ORCID iDs will not appear in the published article. Only the individual author can complete the verification step; PLOS staff cannot verify ORCID iDs on behalf of authors.

We look forward to receiving your revised manuscript.

Kind regards,

Hugues C. Nana Djeunga, PhD

Academic Editor

PLOS Neglected Tropical Diseases

Lucienne Tritten

Section Editor

PLOS Neglected Tropical Diseases

Shaden Kamhawi

co-Editor-in-Chief

PLOS Neglected Tropical Diseases

orcid.org/0000-0003-4304-636XX

Paul Brindley

co-Editor-in-Chief

PLOS Neglected Tropical Diseases

orcid.org/0000-0003-1765-0002

**Additional Editor Comments:**

Dear Professor Hermine Boukeng Jatsa,

Thank you for revising your manuscript entitled “Schistosomiasis mansoni and alcohol abuse comorbidity: prevalence and risk factors among adults in Makenene, Cameroon” (PNTD-D-25-01931R1). Your revised submission, along with your responses to the reviewers’ comments, has been carefully evaluated by the Reviewers and members of the editorial board.

The reviewers commend the revisions, which have significantly improved the quality and relevance of the manuscript. However, they have identified a few minor issues that should be addressed before the manuscript can be formally accepted for publication in PLOS Neglected Tropical Diseases.

Yours sincerely,

Hugues C. Nana Djeunga,

Academic Editor.

**Journal Requirements:**

If the reviewer comments include a recommendation to cite specific previously published works, please review and evaluate these publications to determine whether they are relevant and should be cited. There is no requirement to cite these works unless the editor has indicated otherwise.

1) Your manuscript's sections are not in the correct order.  Please amend to the following order: Abstract, Introduction, Results, Discussion, and Methods

**Reviewers' comments:**

Reviewer's Responses to Questions

**Key Review Criteria Required for Acceptance?**

As you describe the new analyses required for acceptance, please consider the following:

**Methods**

-Are the objectives of the study clearly articulated with a clear testable hypothesis stated?

-Is the study design appropriate to address the stated objectives?

-Is the population clearly described and appropriate for the hypothesis being tested?

-Is the sample size sufficient to ensure adequate power to address the hypothesis being tested?

-Were correct statistical analysis used to support conclusions?

-Are there concerns about ethical or regulatory requirements being met?

Reviewer #1: The study is novel and addresses an important and underexplored intersection between two significant public health problems intestinal schistosomiasis and alcohol abuse. The objectives are clearly articulated, specifically aiming to investigate the prevalence and risk factors associated with Schistosoma mansoni infection and alcohol abuse comorbidity in Makenene, Cameroon.

The justification provided adequately supports the chosen research design, and the sample size appears sufficient to ensure appropriate statistical power to address the study objectives.

The study demonstrates adherence to ethical and regulatory standards, which strengthens its credibility. While the analytical approach is comprehensive, I have clearly indicated my limitations in undertaking a detailed and extensive statistical review of the analyses conducted.

Reviewer #2: The manuscript meets all key review criteria for acceptance. The authors have thoroughly addressed all reviewer critiques, including adding new analyses (comorbidity risk factors in Table 17), correcting statistical methods (median/IQR, normality testing), expanding limitations, and strengthening the discussion of clinical and public health implications.

Reviewer #3: (No Response)

Reviewer #4: (No Response)

**********

**Results**

-Does the analysis presented match the analysis plan?

-Are the results clearly and completely presented?

-Are the figures (Tables, Images) of sufficient quality for clarity?

Reviewer #1: The results are presented clearly and effectively, particularly through well-structured tables that are concise, not overloaded, and easy to interpret. This enhances readability and allows for straightforward understanding of key findings. The quality of the tables is commendable and supports the overall clarity of the manuscript.

Reviewer #2: The results section meets PLOS NTD standards for acceptance. The authors have:

• Presented all findings systematically

• Included appropriate statistical measures (OR, AOR, 95% CI, p-values)

• Added the requested interaction analysis (Table 8)

• Added the requested comorbidity risk factor analysis (Table 17)

• Removed off-topic diagnostic comparison sections

• Ensured all tables are clear and properly labeled

Therefore, no further revisions to the results section are required.

Reviewer #3: (No Response)

Reviewer #4: (No Response)

**********

**Conclusions**

-Are the conclusions supported by the data presented?

-Are the limitations of analysis clearly described?

-Do the authors discuss how these data can be helpful to advance our understanding of the topic under study?

-Is public health relevance addressed?

Reviewer #1: The findings convincingly highlight the concomitance of intestinal schistosomiasis and alcohol abuse, underscoring the compounded risk these conditions pose, particularly in relation to liver morbidity. The results contribute valuable evidence to an area that has received limited attention and provide a strong basis for the discussion and conclusions drawn.

The study fully demonstrates that the coexistence of intestinal schistosomiasis and alcohol abuse represents a significant and potentially underrecognized public health concern. The paper effectively highlights the risk of exacerbated liver complications resulting from this comorbidity, particularly in endemic settings such as Cameroon.

By drawing attention to the dual burden of infectious and behavioral risk factors, the study makes an important contribution to the scientific literature and underscores the need for integrated approaches to disease control and health promotion.

The manuscript makes a compelling case for integrating alcohol abuse prevention strategies into schistosomiasis control programs. This integrated approach has the potential to enhance the effectiveness of public health interventions aimed at reducing disease burden and preventing complications.

Given its novelty, relevance, and overall quality, the study should receive attention within the scientific and public health communities.

Reviewer #2: The conclusions section meets all PLOS NTD requirements for acceptance. The authors have:

1. Drawn conclusions directly supported by their data. All conclusions were derived directly from the presented results (Tables 2, 5, 7, 17).

2. Provided a thorough limitations section addressing key methodological concerns. Example: a comprehensive limitations section (lines 844-859) covering diagnostic, self-report, generalizability, and unmeasured confounders.

3. Clearly articulated how their findings advance understanding of schistosomiasis-alcohol comorbidity through novel findings on community-specific dynamics, synergistic pathology, and praziquantel metabolism concerns.

4. Connected their findings to specific public health interventions and global elimination targets. This was through specific recommendations for integrated interventions, adult inclusion in MDA, and alignment with the WHO roadmap.

Recommendation: Accept as is, with optional minor clarification regarding adulterated whisky in the conclusion.

Reviewer #3: (No Response)

Reviewer #4: (No Response)

**********

**Editorial and Data Presentation Modifications?**

Use this section for editorial suggestions as well as relatively minor modifications of existing data that would enhance clarity. If the only modifications needed are minor and/or editorial, you may wish to recommend “Minor Revision” or “Accept”.

Reviewer #1: Accept

Reviewer #2: The manuscript has been thoroughly revised, and all substantive reviewer concerns have been addressed. Only minor editorial issues remain, none of which affect scientific validity or clarity. Therefore, the paper is suitable for publication in PLOS Neglected Tropical Diseases.

Minor Editorial Suggestions (Optional, Not Required for Acceptance)

The following suggestions would further enhance clarity and consistency, but do not necessitate another round of review.

1. Standardize decimal separator

Inconsistent use of comma and period as decimal separator (e.g., "40,7%" in line 173 vs. "40.7%" elsewhere). Please check line 173 (sample size calculation). It states, "estimated at 40,7% in 2019 [8]". I suggest it as "estimated at 40.7% in 2019 [8]".

2. Clarify the adulterated whisky mentioned in the conclusion

The conclusion states that regular consumption of adulterated whisky exacerbates the dual health burden, but the discussion clarifies that this beverage was not significantly associated with comorbidity (only with alcohol abuse). See conclusion, lines 864-866. You indicated that "regular consumption of adulterated whisky, palm wine, and beer... exacerbates this dual health burden".

Ideally, it should be: "regular consumption of palm wine and beer, along with factors associated with agricultural activities and frequent human-contaminated water sources, exacerbates this dual health burden" (Remove "adulterated whisky" from the conclusion since it was not a significant comorbidity predictor; keep it in the discussion where the nuanced finding is explained.).

Note: this improves accuracy and consistency between the conclusion and discussion.

3. Standardize "Kato-Katz" hyphenation

In some instances, use "Kato Katz" without hyphen. See Table 8 footnote: "diagnosed by the Kato Katz". I suggest you change to "Kato-Katz" throughout.

4. Table formatting for production

Several tables (e.g., Tables 7, 9, 10) contain line breaks within cells that may need adjustment during typesetting. This is normal and will be handled by the production team. No action needed from authors.

Overall Recommendation: Accept

Reviewer #3: (No Response)

Reviewer #4: (No Response)

**********

**Summary and General Comments**

Use this section to provide overall comments, discuss strengths/weaknesses of the study, novelty, significance, general execution and scholarship. You may also include additional comments for the author, including concerns about dual publication, research ethics, or publication ethics. If requesting major revision, please articulate the new experiments that are needed.

Reviewer #1: The study fully demonstrates that the coexistence of intestinal schistosomiasis and alcohol abuse represents a significant and potentially underrecognized public health concern. The paper effectively highlights the risk of exacerbated liver complications resulting from this comorbidity, particularly in endemic settings such as Cameroon.

By drawing attention to the dual burden of infectious and behavioral risk factors, the study makes an important contribution to the scientific literature and underscores the need for integrated approaches to disease control and health promotion.

The manuscript makes a compelling case for integrating alcohol abuse prevention strategies into schistosomiasis control programs. This integrated approach has the potential to enhance the effectiveness of public health interventions aimed at reducing disease burden and preventing complications.

Given its novelty, relevance, and overall quality, the study should receive attention within the scientific and public health communities.

Reviewer #2: Overall Assessment

1. This manuscript by Yimgoua and colleagues presents a novel, well-executed, and methodologically sound investigation of the comorbidity between Schistosoma mansoni infection and alcohol abuse among adults in Makenene, Cameroon. The study addresses a significant gap in the literature by examining the intersection of an infectious disease (schistosomiasis) and a behavioral risk factor (alcohol misuse) that share a common target organ, the liver, yet have rarely been studied together in endemic populations.

2. The authors have responded comprehensively to all four reviewers' critiques, resulting in a substantially improved manuscript. Key revisions include the following: (1) addition of an explicit testable hypothesis; (2) removal of extraneous diagnostic test comparisons to focus on comorbidity; (3) correction of statistical methods (median/IQR for non-normal data; geometric mean for EPG); (4) new multivariate analysis of factors associated with comorbidity (Table 17); (5) interaction analysis between parasite burden and alcohol use (Table 8); (6) expanded limitations section; and (7) strengthened discussion of clinical and public health implications, including potential synergistic hepatic damage and effects on praziquantel metabolism.

Reviewer #3: (No Response)

Reviewer #4: (No Response)

**********

PLOS authors have the option to publish the peer review history of their article (what does this mean?). If published, this will include your full peer review and any attached files.

If you choose “no”, your identity will remain anonymous but your review may still be made public.

**Do you want your identity to be public for this peer review?**  For information about this choice, including consent withdrawal, please see our Privacy Policy.

Reviewer #1: **Yes:** Nana KWadwo Biritwum

Reviewer #2: No

Reviewer #3: **Yes:** Linda Djune Yemeli (PhD)

Reviewer #4: No

[NOTE: If reviewer comments were submitted as an attachment file, they will be attached to this email and accessible via the submission site. Please log into your account, locate the manuscript record, and check for the action link "View Attachments". If this link does not appear, there are no attachment files.]

**Figure resubmission:**

While revising your submission, we strongly recommend that you use PLOS’s NAAS tool (https://ngplosjournals.pagemajik.ai/artanalysis) to test your figure files. NAAS can convert your figure files to the TIFF file type and meet basic requirements (such as print size, resolution), or provide you with a report on issues that do not meet our requirements and that NAAS cannot fix.

After uploading your figures to PLOS’s NAAS tool - https://ngplosjournals.pagemajik.ai/artanalysis, NAAS will process the files provided and display the results in the "Uploaded Files" section of the page as the processing is complete. If the uploaded figures meet our requirements (or NAAS is able to fix the files to meet our requirements), the figure will be marked as "fixed" above. If NAAS is unable to fix the files, a red "failed" label will appear above. When NAAS has confirmed that the figure files meet our requirements, please download the file via the download option, and include these NAAS processed figure files when submitting your revised manuscript.

**Reproducibility:**

To enhance the reproducibility of your results, we recommend that authors of applicable studies deposit laboratory protocols in protocols.io, where a protocol can be assigned its own identifier (DOI) such that it can be cited independently in the future. Additionally, PLOS ONE offers an option to publish peer-reviewed clinical study protocols. Read more information on sharing protocols at https://plos.org/protocols?utm_medium=editorial-email&utm_source=authorletters&utm_campaign=protocols

Attachment Submitted filename: review report.docx

# Author comment: Author response to Decision Letter 2

**DOI:** 10.1371/journal.pntd.0013687.r004

8 May 2026

Attachment Submitted filename: Response_to_reviewers_auresp_2.docx

# Editor report: Decision Letter 2

**DOI:** 10.1371/journal.pntd.0013687.r005

**Authors:** Hugues C. Nana Djeunga, Lucienne Tritten

19 Jun 2026

PNTD-D-25-01931R2Schistosomiasis mansoni and alcohol abuse comorbidity: prevalence and risk factors among adults in Makenene, CameroonPLOS Neglected Tropical Diseases Dear Dr. Jatsa, Thank you for submitting your manuscript to PLOS Neglected Tropical Diseases. After careful consideration, we feel that it has merit but does not fully meet PLOS Neglected Tropical Diseases's publication criteria as it currently stands. Therefore, we invite you to submit a revised version of the manuscript that addresses the points raised during the review process. Please submit your revised manuscript by July 18, 2026. If you will need more time than this to complete your revisions, please reply to this message or contact the journal office at plosntds@plos.org. When you're ready to submit your revision, log on to https://www.editorialmanager.com/pntd/ and select the 'Submissions Needing Revision' folder to locate your manuscript file. Please include the following items when submitting your revised manuscript:* A letter that responds to each point raised by the editor and reviewer(s). You should upload this letter as a separate file labeled 'Response to Reviewers'. This file does not need to include responses to any formatting updates and technical items listed in the 'Journal Requirements' section below.* A marked-up copy of your manuscript that highlights changes made to the original version. You should upload this as a separate file labeled 'Revised Manuscript with Track Changes'.* An unmarked version of your revised paper without tracked changes. You should upload this as a separate file labeled 'Manuscript'. If you would like to make changes to your financial disclosure, competing interests statement, or data availability statement, please make these updates within the submission form at the time of resubmission. Guidelines for resubmitting your figure files are available below the reviewer comments at the end of this letter. As the corresponding author, your ORCID iD is verified in the submission system and will appear in the published article. PLOS supports the use of ORCID, and we encourage all coauthors to register for an ORCID iD and use it as well. Please encourage your coauthors to verify their ORCID iD within the submission system before final acceptance, as unverified ORCID iDs will not appear in the published article. Only the individual author can complete the verification step; PLOS staff cannot verify ORCID iDs on behalf of authors.We look forward to receiving your revised manuscript. Kind regards, Hugues C. Nana Djeunga, PhDAcademic EditorPLOS Neglected Tropical Diseases Lucienne TrittenSection EditorPLOS Neglected Tropical Diseases

Shaden Kamhawi

co-Editor-in-Chief

PLOS Neglected Tropical Diseases

orcid.org/0000-0003-4304-636XX

Paul Brindley

co-Editor-in-Chief

PLOS Neglected Tropical Diseases

orcid.org/0000-0003-1765-0002

**Additional Editor Comments (if provided):**  Dear Professor Hermine Boukeng Jatsa,

Thank you for submitting the second revised version of your manuscript entitled “Schistosomiasis mansoni and alcohol abuse comorbidity: prevalence and risk factors among adults in Makenene, Cameroon” (PNTD-D-25-01931R2). Your revised submission, together with your responses to the reviewers’ comments, has been carefully evaluated by the Editorial Board.

We are pleased to note that the reviewers’ comments have been satisfactorily addressed. However, during our final checks, we realized that the comments from Reviewer #4 were inadvertently not forwarded to you. We have now included them below for your attention before the manuscript can be formally accepted for publication in PLOS Neglected Tropical Diseases. We sincerely apologize for this oversight and any inconvenience it may have caused.

Please revise and resubmit your manuscript by June 25, 2026.

Yours sincerely,

Hugues C. Nana Djeunga,

Handling Editor.

----------------------------

Commnents Reviewer #4

This manuscript PNTD-D-25-01931 of Yimgoua et al entitled "Schistosomiasis mansoni-alcoholism comorbidity: prevalence and risk factors among adults in Makenene, Cameroon" treat an interesting topic related to intestinal schistosomiasis and alcoholism as well as their comorbitity in schistosomiasis endemic area of Makenene in Cameroon.

Major comment

The manuscript has been considerably improved. However, there is still some corrections to made notably the tables. The manuscripts contains 17 tables. This is too much and there is a need to reduced considerably the number of tables.

Minor points

1- Lines 29 to 31: The prevalence was given for the two techniques used, but it is not clear how the final prevalence was obtained

2- Line 151 -153: is there a difference between village and neighborhood? Did the authors consider Mock sud and Mock centre as villages or neighbordhood? Ii Baloua a village or a neighbordhood?

3- Lines 232-235; Must move to the result section

4- Lines 235- 238: Repetition (see lines 228 - 230)

5- Lines 234-235: sentence to be rephrased

6- Line 248: What do the authors mean by “Kato solution”?

7- Line 271: “middle adulthood (50 – 59 years old)”: it must be (40-59 years old)

8- Lines 310-312: The is a need of some clarifications: 109 POCCAA+ + 75 KK+ = 184; will be good to clarify how many were positive for both tests. Table 2 can be deleted.

9- Lines 328 to 329 must be deleted

10- line 331: The first prevalence must be deleted; Moreove, “rate” which is in front of the second prevalence must be deleted

11- Line 346: The “to” which is in front of “21.9% (95% CI: 12.9–31.4)” must be deleted

12- Lines 425 to 427 must be deleted

13- Lines 467 to 468 must be deleted

14- Table 8 must be deleted

15- Table 11: Data not available; This table can be deleted

16- Line 533 must be deleted

17 - Line 562 must be deleted

18- Lines 568-570: Repetition of lines 508-510

19- Lines 596-598: must be deleted

20- Line 632- 634: not clear what the authors want to highlight

21- Line 637 641: Comparing the disease prevalence in adults and school-aged children is not relevant. The two groups of inhabitants are not subjected to level of exposition and slo to the same control measures.

Overall, this manuscript still needs some revision before been accepted for publication in Plos NTD    [Note: HTML markup is below. Please do not edit.] **Reviewers' comments:**    [NOTE: If reviewer comments were submitted as an attachment file, they will be attached to this email and accessible via the submission site. Please log into your account, locate the manuscript record, and check for the action link "View Attachments". If this link does not appear, there are no attachment files.] **Figure resubmission:**   While revising your submission, we strongly recommend that you use PLOS’s NAAS tool (https://ngplosjournals.pagemajik.ai/artanalysis) to test your figure files. NAAS can convert your figure files to the TIFF file type and meet basic requirements (such as print size, resolution), or provide you with a report on issues that do not meet our requirements and that NAAS cannot fix.

After uploading your figures to PLOS’s NAAS tool - https://ngplosjournals.pagemajik.ai/artanalysis, NAAS will process the files provided and display the results in the "Uploaded Files" section of the page as the processing is complete. If the uploaded figures meet our requirements (or NAAS is able to fix the files to meet our requirements), the figure will be marked as "fixed" above. If NAAS is unable to fix the files, a red "failed" label will appear above. When NAAS has confirmed that the figure files meet our requirements, please download the file via the download option, and include these NAAS processed figure files when submitting your revised manuscript. **Reproducibility:**  To enhance the reproducibility of your results, we recommend that authors of applicable studies deposit laboratory protocols in protocols.io, where a protocol can be assigned its own identifier (DOI) such that it can be cited independently in the future. Additionally, PLOS ONE offers an option to publish peer-reviewed clinical study protocols. Read more information on sharing protocols at https://plos.org/protocols?utm_medium=editorial-email&utm_source=authorletters&utm_campaign=protocols

# Author comment: Author response to Decision Letter 3

**DOI:** 10.1371/journal.pntd.0013687.r006

23 Jun 2026

Attachment Submitted filename: Responses to Reviewer #4.docx

# Editor report: Decision Letter 3

**DOI:** 10.1371/journal.pntd.0013687.r007

**Authors:** Hugues C. Nana Djeunga, Lucienne Tritten

24 Jun 2026

Dear Dr. Jatsa,

We are pleased to inform you that your manuscript 'Schistosomiasis mansoni and alcohol abuse comorbidity: prevalence and risk factors among adults in Makenene, Cameroon' has been provisionally accepted for publication in PLOS Neglected Tropical Diseases.

Before your manuscript can be formally accepted you will need to complete some formatting changes, which you will receive in a follow up email. A member of our team will be in touch with a set of requests.

Please note that your manuscript will not be scheduled for publication until you have made the required changes, so a swift response is appreciated.

IMPORTANT: The editorial review process is now complete. PLOS will only permit corrections to spelling, formatting or significant scientific errors from this point onwards. Requests for major changes, or any which affect the scientific understanding of your work, will cause delays to the publication date of your manuscript.

Should you, your institution's press office or the journal office choose to press release your paper, you will automatically be opted out of early publication. We ask that you notify us now if you or your institution is planning to press release the article. All press must be co-ordinated with PLOS.

Thank you again for supporting Open Access publishing; we are looking forward to publishing your work in PLOS Neglected Tropical Diseases.

Best regards,

Hugues C. Nana Djeunga, PhD

Academic Editor

PLOS Neglected Tropical Diseases

Lucienne Tritten

Section Editor

PLOS Neglected Tropical Diseases

Shaden Kamhawi

co-Editor-in-Chief

PLOS Neglected Tropical Diseases

orcid.org/0000-0003-4304-636XX

Paul Brindley

co-Editor-in-Chief

PLOS Neglected Tropical Diseases

orcid.org/0000-0003-1765-0002

***********************************************************

# Editor report: Acceptance letter

**DOI:** 10.1371/journal.pntd.0013687.r008

**Authors:** Hugues C. Nana Djeunga, Lucienne Tritten

Dear Pr Boukeng Jatsa,

We are delighted to inform you that your manuscript, "Schistosomiasis mansoni and alcohol abuse comorbidity: prevalence and risk factors among adults in Makenene, Cameroon," has been formally accepted for publication in PLOS Neglected Tropical Diseases.

We have now passed your article onto the PLOS Production Department who will complete the rest of the publication process. All authors will receive a confirmation email upon publication.

The corresponding author will soon be receiving a typeset proof for review, to ensure errors have not been introduced during production. Please review the PDF proof of your manuscript carefully, as this is the last chance to correct any scientific or type-setting errors. Please note that major changes, or those which affect the scientific understanding of the work, will likely cause delays to the publication date of your manuscript. Note: Proofs for Front Matter articles (Editorial, Viewpoint, Symposium, Review, etc...) are generated on a different schedule and may not be made available as quickly.

Soon after your final files are uploaded, the early version of your manuscript will be published online unless you opted out of this process. The date of the early version will be your article's publication date. The final article will be published to the same URL, and all versions of the paper will be accessible to readers.

For Research Articles, you will receive an invoice from PLOS for your publication fee after your manuscript has reached the completed accept phase. If you receive an email requesting payment before acceptance or for any other service, this may be a phishing scheme. Learn how to identify phishing emails and protect your accounts at https://explore.plos.org/phishing.

Thank you again for supporting open-access publishing; we are looking forward to publishing your work in PLOS Neglected Tropical Diseases.

Best regards,

Shaden Kamhawi

co-Editor-in-Chief

PLOS Neglected Tropical Diseases

Paul Brindley

co-Editor-in-Chief

PLOS Neglected Tropical Diseases
